# A highly diversified NLR cluster in melon contains homologs that confer powdery mildew and aphid resistance

**DOI:** 10.1093/hr/uhad256

**Published:** 2023-12-13

**Authors:** Nathalie Boissot, Veronique Chovelon, Vincent Rittener-Ruff, Nathalie Giovinazzo, Pascale Mistral, Michel Pitrat, Myriam Charpentier, Christelle Troadec, Abdelhafid Bendahmane, Catherine Dogimont

**Affiliations:** INRAE, GAFL, 84143 Montfavet, France; INRAE, GAFL, 84143 Montfavet, France; INRAE, GAFL, 84143 Montfavet, France; INRAE, GAFL, 84143 Montfavet, France; INRAE, GAFL, 84143 Montfavet, France; INRAE, GAFL, 84143 Montfavet, France; INRAE, IPS2, 91190 Gif-sur-Yvette, France; John Innes Centre, Department Cell & Developmental Biology, Colney Lane, Norwich NR4 7UH, UK; INRAE, IPS2, 91190 Gif-sur-Yvette, France; INRAE, IPS2, 91190 Gif-sur-Yvette, France; INRAE, GAFL, 84143 Montfavet, France

## Abstract

*Podosphaera xanthii* is the main causal agent of powdery mildew (PM) on Cucurbitaceae. In *Cucumis melo*, the *Pm*-*w* resistance gene, which confers resistance to *P*. *xanthii,* is located on chromosome 5 in a cluster of nucleotide-binding leucine-rich repeat receptors (NLRs). We used positional cloning and transgenesis, to isolate the *Pm-w^WMR 29^* gene encoding a coiled-coil NLR (CC-NLR). *Pm-w^WMR 29^* conferred high level of resistance to race 1 of PM and intermediate level of resistance to race 3 of PM. *Pm-w^WMR 29^* turned out to be a homolog of the *Aphis gossypii* resistance gene *Vat-1^PI 161375^*. We confirmed that *Pm-w^WMR 29^* did not confer resistance to aphids, while *Vat-1^PI 161375^* did not confer resistance to PM*.* We showed that both homologs were included in a highly diversified cluster of NLRs, the *Vat* cluster. Specific *Vat-1^PI 161375^* and *Pm-w^WMR 29^* markers were present in 10% to 13% of 678 accessions representative of wild and cultivated melon types worldwide. Phylogenic reconstruction of 34 protein homologs of Vat-1^PI 161375^ and Pm-w^WMR *29*^ identified in 24 melon accessions revealed an ancestor with four R65aa—a specific motif in the LRR domain, evolved towards aphid and virus resistance, while an ancestor with five R65aa evolved towards PM resistance. The complexity of the cluster comprising the *Vat/Pm-w* genes and its diversity in melon suggest that *Vat* homologs may contribute to the recognition of a broad range of yet to be identified pests and pathogens.

## Introduction

Melon (*Cucumis melo* L.) belonging to the Cucurbitaceae family is a major economically important horticultural crop worldwide. Melon is highly appreciated for its edible produce that is widely consumed both as a fruit and a vegetable. Open fields and/or greenhouses grown melons can be affected by a plethora of pests and diseases such as powdery mildew (PM). The latter is caused by a range of biotrophic fungi (*Erysiphales*) that may infect leaves, stems, flowers, and fruits, thereby substantially impacting the fruit quality and quantity [26]. PMs establish highly integrated relationships with their hosts through the development of a specialized parasitism-related structure, or so-called haustorium, that may induce compatible or incompatible interactions [5]. *Podosphaera xanthii* is the main causal agent of cucurbit PM and a total of 21 *P. xanthii* races have been identified on melon [15], based on their pathogenicity or virulence on known resistant lines [30]. Sources of resistance to PMs have been identified and the PMR 45, PMR 5, PMR 6, and WMR 29 accessions (*Cantalupensis* group) as well as MR-1, PI 124111, and PI 414723 accessions (*Momordica* group), PI 313970 (90625), and PI 482420 (TGR 1551) accessions (*Acidulus* group) and the PI 124112 accession are considered as resistant germplasm [33, 37]. To date, 25 dominant genes, two recessive genes and seven QTLs have been identified in melon from a number of accessions [15, 47]. The dominant loci are mostly clustered on chromosomes 2, 5, and 12, thus indicating a potential allelic relationship of those in the same cluster, but no dominant typical resistance genes have been cloned so far. The *Pm-w* locus involved in resistance to *P. xanthii* was located at less than 2.5 cM from *Vat* [35], a nucleotide-binding leucine rich-repeat (NLR) resistance gene located on chromosome 5, in a telomeric region containing the highest density of NLR genes found in the melon genome [22].

Plant NLRs are thought to have co-evolved with the pathogenicity factors of the challenging pests and pathogens. As such, they tend to be among the most polymorphic genes in plant genomes, both in terms of sequence diversity and copy-number variation [44]). NLR genes code for proteins recognizing pathogen-secreted effectors within cells and give rise to effector-triggered immunity (ETI) which, combined with basal immunity (pattern-triggered immunity [PTI]), induce robust resistance responses [38, 46]. NLR proteins are thought to function as a sensor, a molecular “switch” between an inactive and an active conformation, and a signaling activator, following the recognition of a pathogen-derived elicitor [14]. In the distant past, NLRs diverged into three classes characterized by the presence of a Toll-interleukin-1 receptor (TIR) domain, a coiled-coil (CC) domain, or a RPW8 domain in the N-terminus [41, 43]**.** Additional unusual domains have been identified, which may contribute to the flexibility of NLRs in perceiving pathogen effectors [25].

In a recent study on several cucurbits, we deciphered the CC-NLR cluster spanning the *Vat* gene [13] and we revealed a unique way of *Vat* genes duplication in the species *C. melo*. Phylogenic studies among melon lines have highlighted the gain and loss of leucine-rich-repeats of 65 amino acids (R65aa) during diversification. In the present paper, we cloned *Vat-1* in the PM-resistant WMR 29 accession. We demonstrated, using transgenic plants, that *Vat-1^WMR29^* triggered resistance to two races of *P. xanthii*, then (*Vat-1^WMR 29^* = *Pm-w*) and did not trigger resistance to viruses when inoculated by *Aphis gossypii,* while the *Vat-1^PI 161375^* gene conferred the reverse phenotype. We investigated the frequency of *Pm-w^WMR 29^*/*Vat-1^PI 161375^* in melon diversity and we identified lines containing both *Pm-w^WMR 29^* and *Vat-1^PI 161375^.* These loci differed for their number of leucine-rich repeat sequences of 65 amino acids (R65aa) in the LRR domain and phylogenetic studies suggested that the R65aa number was a key factor in the resistance specificity, but SNPs switched resistance to susceptibility.

## Results

### Positional cloning of *Pm-w*^*WMR 29*^

**Figure 1:**
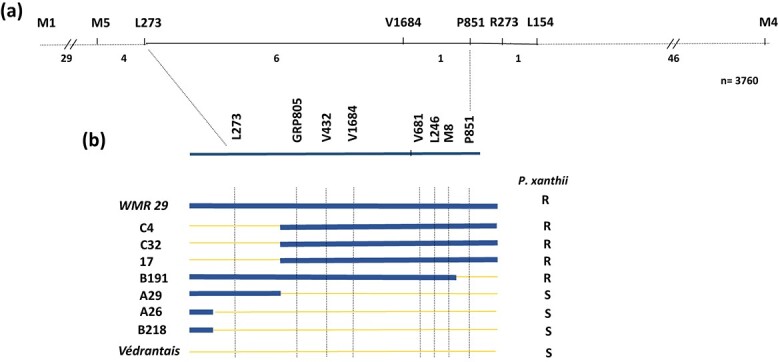
High resolution map of the *Pm-w* locus from WMR 29. (a) Genetic map of the region containing the *Pm-w* locus. The molecular markers are indicated on the top and the number of recombinant plants is indicated between markers. (b) Recombination events in 7 out of 87 recombinant plants between PM-susceptible Védrantais and PM-resistant WMR 29. Thick green and thin orange lanes represent DNA fragments from WMR 29 and Védrantais, respectively. R and S correspond to resistance and susceptibility to *Podosphaera xanthii* race3 infection.

We developed a positional cloning approach to isolate the *Pm-w* locus using a cross between the PM-susceptible Védrantais cultivar and the PM-resistant WMR 29 cultivar. Ninety-five plants of a back-cross population [(Védrantais x WMR 29) x Védrantais] were assessed for *P. xanthii* resistance race 3 (00Sm39 isolate) using a leaf disk test. Considering the tight genetic link between *Vat* and *Pm-w*, we screened these plants with M1 and M4 PCR-based markers previously demonstrated to encompass the *Vat* locus [17]. We identified one recombinant plant and mapped *Pm-w* in the interval delimited by both markers. Extending the study to 3760 back-cross plants, we identified 87 plants with crossovers between M1 and M4, and we obtained, by selfing from this 87 BC1 plants, progenies called BC_1_I_1_. Powdery mildew resistance was recorded for each recombinant individual plant and for their corresponding BC_1_I_1_ progeny. Recombinants were genotyped using additional molecular markers and the *Pm-w* locus was located between the L273 and P851 markers (Figure 1; Table S1a).

**Figure 2:**
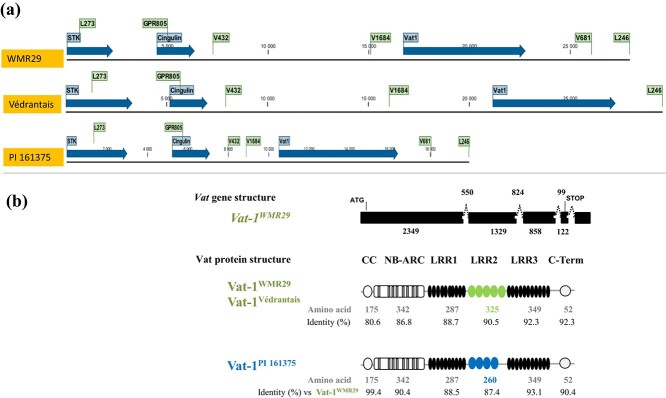
Annotation of WMR 29, Védrantais and PI 161375 sequences. (a) Genomic sequences from L273 to L246 markers on chromosome 5 (Védrantais sequence from [32]) and PI 161375 sequence from [13]). Gene in blue, Primer binding site in green. **(b)***Vat* homolog gene and protein structures**.** Gene structure: thick lines indicate exons; broken lines indicate introns; exon and intron sizes are indicated in bp. Schematic diagrams of Vat-1 proteins R65aa: highly conserved leucine-rich repeat sequences of 65 amino acids in the LRR2 domain

To physically delineate the *Pm-w* locus, a bacterial artificial chromosome (BAC) library was constructed from the nuclear DNA of WMR 29. The L273 and P851 markers were used to screen this BAC library and six BAC clones encompassing both markers were isolated. A 33-kb subclone was sequenced and annotated. Predicted gene sequences were compared to the corresponding *Vat* cluster sequence in Védrantais and PI 161375 (Figure 2a). The first predicted gene after the L273 marker encoded a cingulin protein and the second predicted gene encoded a *Vat* homolog, which is most probably the first of a series of *Vat*- homologs and, according to the nomenclature for the *Vat* cluster [13], we named it *Vat-1^WMR 29^*. The complete coding sequence was obtained by RT-PCR; it encoded a predicted protein of 1532 amino acids (Figure 2b, Data S1) as Vat-1^Vedrantais^, and they shared very similar primary structure: a CC domain, an NB-ARC domain, an LRR domain with three remarkable parts called LRR1, LRR2, LRR3, and a C-Term domain. Nevertheless, Vat-1^WMR 29^ shared only 87.96% identity with Vat-1^Vedrantais^. We compared *Vat-1^WMR 29^* to *Vat-1^PI 161375^*, the only *Vat* homolog conferring a known phenotype, e.g. resistance to aphids. They encoded proteins sharing 91.34%. identity. The LRR domain was the most variable between the two alleles. It included two series of 10 and 11 imperfect LRR motifs (LRR1 and LRR3) separated by highly conserved repeats whose number differed (Figure 2b). The Vat-1^WMR 29^ protein had five highly homologous leucine-rich repeat sequences of 65 amino acids (R65aa) in the LRR2 domain, whereas the Vat-1^PI 161375^ protein had only four.

### *Vat-1* ^*WMR 29*^ gene is responsible for powdery mildew resistance

**Figure 3:**
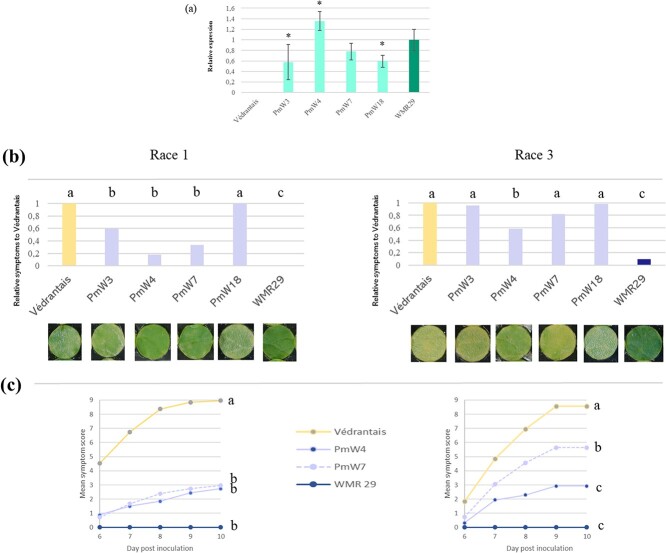
Characterization of four melon transgenic lines (PmW3, PmW4, PmW7, PmW18). (a) Constitutive expression of *Vat-1^WMR 29^* relative to expression in WMR 29. Relative expression (mean of five biological replicates) normalized with three reference genes (*TIP41*, *actin, TUA*). Error bars indicate standard deviations. Asterisks indicate significant differences between transgenic plants and the PM resistant WMR 29 wild type (Student’s t-test). (b-c) Powdery mildew resistance tests. *P. xanthii* race 1 (Sm3isolate) and *P. xanthii* race 3 (00Sm39 isolate) were inoculated to leaf disks; [mycelium colonization-sporulation] was scored from 0 to 9. (b) Four experiments were carried out with each PM race (one by PmW transgenic lines). Bars showed relative scores to Védrantais score ten days post inoculation. Four Steel-Dwass-Critchlow-Fligner / bilatéral tests were carried out on scores allowing to define groups for each set [Védrantais, transgenic line and WMR29]. Letters indicate significant statistical groups of scores (p < 0.05). (c) Two experiments were carried out (one by PM race). Points showed mean score from 6 to 10 days. Letters indicate significant statistical groups for the areas under the curve (ANOVA and Bonferoni pair test (p < 0.05)).

Of the two genes in the interval L273–L246, the *Vat-1^WMR 29^* was the best candidate to confer resistance to PM. Then, we conducted complementation experiments with an 11-kb DNA fragment harboring the *Vat-1^WMR 29^* gene and its 5′-3′-UTR regions. This fragment was introduced into the Védrantais PM-susceptible melon line via *Agrobacterium*-mediated transformation. Four independent diploid *Vat-1^WMR 29^*-Védrantais transgenic plants (T0) harboring a single T-DNA insertion were produced and (T1) and (T2) progenies were obtained by self-pollination and homozygous (T2) were selected for further characterization. We carried out quantitative real-time polymerase chain reaction (Q-RT-PCR) of *Vat-1^WMR 29^* transgene from no-infected plants of Védrantais, WMR29 and the four transgenic lines called PmW3, PmW4 PmW7 and PmW18. As expected, *Vat-1^WMR 29^* was not expressed in Védrantais but expressed in WMR29. The *Vat-1^WMR 29^* expression in the four transgenic lines suggested that insert events resulted in different expression levels. Two transgenic lines expressed *Vat-1^WMR 29^* at a similar (in PmW7) or higher (in PmW4) level than WMR 29 while *Vat-1^WMR 29^* was more weakly expressed in the two other transgenic lines (PmW3 and PmW18) (Figure 3a).

Thee*Vat-1^WMR29^*-Védrantais transgenic lines were assessed for resistance 10 days after inoculation by two *P. xanthii* isolates, i.e. race 1 (Sm3) and race 3 (00Sm39), using a leaf disk procedure. WMR29 exhibited total resistance to race 1 (no symptoms, score 0) while Védrantais scored from 7.5 to 8.5. The transgenic PmW18 was as susceptible as Védrantais, while PmW4, PmW7 and PmW3 had respectively 82%, 67%, and 40% less symptoms than Védrantais but less resistant than WMR29 (Figure 3b, left). After inoculation by race 3, some WMR29 disks exhibited symptoms (mean score 0.8), while all Védrantais disks scored 9 revealing a higher aggressiveness of *P. xanthii* race 3 compared to race 1. The only transgenic line less susceptible to race 3 than Védrantais was PmW4 with 42% less symptoms but PmW4 was less resistant than WMR29 (Figure 3b, right). We studied more in details the PM resistance in PmW4 and PmW7 following symptoms from 6 to 10 days after PM inoculation. PmW4 and PmW7 exhibited a high level of resistance to race 1 (Figure 3c, left) while PmW4 was significantly more resistance to race 3 than PmW7 (Figure 3c, right).

Therefore, PmW3 and PmW18 that less expressed *Vat-1^WMR 29^* than WMR 29 exhibited no resistance or weak resistance to *P. xanthii* races 1 and 3, while PmW4 and PmW7 that expressed *Vat-1^WMR 29^* at least as WMR29 exhibited high or intermediate resistance to PM races 1 and 3. Actually, high level of constitutive expression of *Vat-1^WMR 29^*, like in PmW4 or WMR 29, might be requested for high level of resistance to PM race 3, suggesting that a frequent recognition was necessary to activate enough early resistance response against the race 3 that was more aggressive than race 1. Moreover, the total resistance to PM races 1 and 3 in WMR 29 suggested that complementary resistance loci are present in WMR 29 and absent in the transgenic plants. To conclude, *Vat-1^WMR 29^* conferred high and intermediate resistance to races 1 and 3 of *P. xanthii*.

Furthermore, we tested PmW4 plants for aphid colonization (*A. gossypii* NM1 clone) and cucumber mosaic virus (CMV) resistance when inoculated by this vector, both phenotypes controlled by *Vat-1^PI 161375^*. As expected PmW4 plants were colonized by *A. gossypii* (Figure S1) and susceptible to CMV. Moreover, we verified that two *Vat-1^PI 161375^-*Védrantais transgenic lines previously obtained [17] did not exhibit PM resistance (Figure S2).

Therefore, *Vat-1^WMR 29^* was shown to confer specific resistance to powdery mildew either high or partial according to the PM race, while its *Vat-1^PI 161375^* allele conferred specific resistance to *A. gossypii.* Therefore*, Vat-1^WMR29^* is called *Pm-w^WMR 29^* hereafter in this paper.

### Four functional *Vat* homologs were sequenced in WMR29

In melon, the number of CC-NLR homolog genes present in the *Vat* cluster and the number of R65aa they contain is a major form of genetic diversity [13]. As several *Vat* homologs were expected in the *Vat* cluster in WMR 29, we used three primer pairs ((Z649F/R; Z6097F/Z6095R; Z5474F/R, Table S1b) to infer the number of homologs. We revealed four additional *Vat* homologs in the WMR 29 cluster. Complete nucleic sequences were obtained for each of them using different primer pairs for long-range PCR, and we confirmed their expression by RT-PCR Table S1b). Complete functional protein sequences were translated from the predicted CDS, except one due to the presence of SNPs that induced early STOP codons (Data S1). Finally, we demonstrated the presence of three functional *Vat* homologs in addition to *Pm-w^WMR29^* in WMR 29. Nevertheless, we did not map them in the genome and we called them *Vat-x^WMR29^, Vat-y^WMR 29^*, and *Vat-z^WMR 29^.* Those *Vat* homologs were manually annotated and contained four, three or one conserved 65 amino-acid leucine-rich repeats (R65aa) in their LRR2 domain. We compared the four *Vat* homologs of WMR 29 to 17 *Vat* homologs containing the same number of R65aa we previously annotated in seven melon lines of diverse origins (PI 161375, Anso 77, Doublon, Payzawat, HS, DHL92, and Harukei-3) for which the whole *Vat* cluster sequence is available [13]. Pairwise comparisons of the Vat proteins are shown in (Table 1).

**Table 1:** Protein sequence identities of the four functional *Vat* homologs in WMR 29 compared to 17 *Vat* homologs obtained from 7 melon genotypes retrieved from Chovelon et al. [13].

***Vat* homologs**		***Pm-w*** ^***WMR 29***^	***Vat-x*** ^***WMR 29***^	***Vat-y*** ^***WMR 29***^	***Vat-z*** ^***WMR 29***^
	**R65aa**	**5**	**4**	**3**	**1**
***Vat-3*** ^***AN***^	**5**	100.00	87.66	90.50	74.80
***Vat-1*** ^***HS***^		88.13	93.09	89.99	75.33
***Vat-3*** ^***DH***^	**4**	87.66	100.00	86.32	75.76
***Vat-1*** ^***AN***^		91.82	89.12	90.88	75.49
***Vat-1*** ^***PI***^		91.34	86.30	94.01	75.77
***Vat-4*** ^***AN***^	**3**	90.57	86.39	99.79	77.40
***Vat-2*** ^***PI***^		90.57	86.54	98.93	77.32
***Vat-2*** ^***HS***^		90.93	86.77	96.37	76.93
***Vat-2*** ^***AN***^		91.73	86.56	95.79	77.55
***Vat-3*** ^***HS***^		92.08	87.95	95.58	78.40
***Vat-1*** ^***DB***^		92.60	87.01	91.08	76.69
***Vat-1*** ^***DH***^		92.60	87.16	91.08	76.84
***Vat-1*** ^***HRK***^		92.60	87.16	91.08	76.84
***Vat-3*** ^***DB***^	**1**	74.80	75.76	77.32	100.00
***Vat-3*** ^***PZ***^		74.80	75.76	77.32	100.00
***Vat-5*** ^***AN***^		74.62	75.43	77.12	99.72
***Vat-2*** ^***HRK***^		73.78	75.54	78.75	99.56

PI 161375 (PI); Anso 77 (AN); Doublon (DB); DHL92 (DH); Payzawat (PZ); Harukei-3 (HRK); HS (HS). R65aa: number of leucine-rich repeat sequences of 65 amino acids in the LRR2 domain of the different *Vat* homologs. *Vat*: *n* (from 1 to 5) indicate the position in the *Vat* cluster

For proteins with five R65aa, Pm-w^WMR 29^ was completely identical to Vat-3^Anso 77^, while it shared 88% identity with Vat-1^HS^. For proteins with four R65aa, Vat-x^WMR 29^ was completely identical to Vat-3^DHL92^ but it shared less than 90% with Vat-1^PI 161375^ and Vat-1^Anso 77^. Concerning proteins with three R65aa, Vat-y^WMR 29^ shared 98.9% and 99.7% identity with Vat-2^PI 161375^ and Vat-4^Anso 77^ and 91% to 96.3% with six other proteins with three R65aa. The four proteins with a single R65aa shared over 99.5% identity.

Therefore, according to the number of R65aa contained in each homolog, 100% identity was observed with Anso 77, DHL92 (a double haploid line containing the *Vat* cluster of Piel de Sapo [13], Doublon, and Payzawat.

### Relationship between powdery mildew and aphid triggered resistance and *Vat* homologs in melon natural diversity

**Figure 4:**
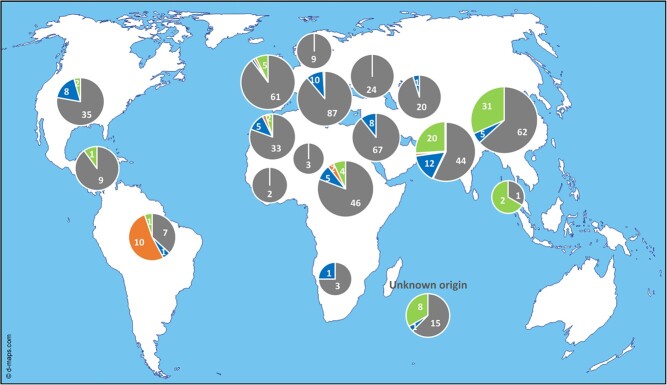
Worldwide frequency of the *Pm-w^WMR 29^* and *Vat-1^PI 161375^* allele markers in melon. Screening of a collection of 678 melon accessions of worldwide origin [39] using specific PCR markers (Table S1b). In blue: number of accessions amplifying *Pm-w^WMR 29^* specific primers (Z5071F/Z5069R). In green: number of accessions amplifying *Vat-1^PI 161375^* specific primers (Z1431F&R). In orange: number of accessions amplifying both markers. In grey: number of accessions amplifying no markers

We investigated the frequency of both *Pm-w^WMR 29^* and *Vat-1^PI 161375^* alleles in 678 melon accessions of worldwide origin (Figure 4) using PCR screening with specific markers (Table S1b).

The *Pm-w^WMR 29^* marker was present in 10.7% of the accessions (including four wild accessions) from almost all geographical regions (Table S2). Among them, we tested 20 accessions for powdery mildew resistance, and 18 were totally or partially resistant to *P. xanthii* (race 1 and/or race 3), while two were susceptible (Table S2). The *Vat-1^PI 161375^* marker was present in 13.5% of the accessions (including ten wild accessions) mostly from India and the Far East. (Table S3). Among them, we tested 52 accessions for resistance to CMV inoculated by two *A. gossypii* clones (NM1 and CUC1), and 48 were resistant (completely or partially) to CMV when it was transmitted by at least one clone (NM1), while four accessions were susceptible (Table S3). Interestingly, both *Vat-1^PI 161375^* and *Pm-w^WMR 29^* markers were amplified in 15 breeding lines (i.e. 2.2%) reaching 52.6% of the tested accessions from South America, mainly from Brazil.

**Figure 5:**
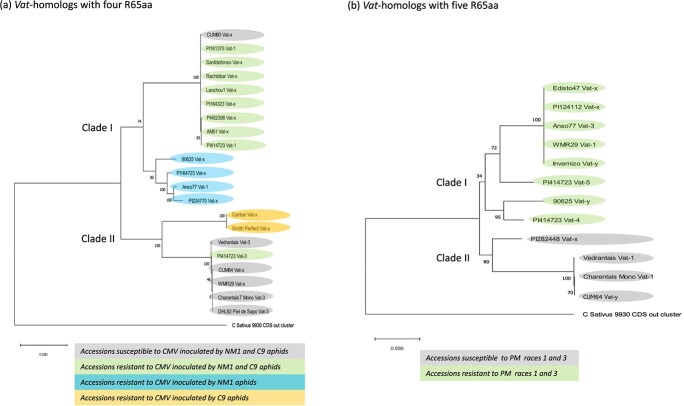
Phylogenic trees of 33 Vat homolog proteins from 26 melon lines and a Vat homolog protein from *Cucumis sativus*, cv 9930 retrieved from [13]. The trees were reconstructed by using the Maximum Likelihood method in MEGA X (Kumar et al. [27]. The percentage of trees in which the associated taxa clustered together is shown next to the branches (100 bootstrap) [27]). (a) Vat homologs with four R65aa (proteins in Data S3), the tree with the highest log likelihood (−8497) is shown, (b) Vat homologs with five R65aa (proteins in Data S4), the tree with the highest log likelihood (−8831) is shown.

We then characterized in detail *Vat* homologs with four (as in *Vat-1^PI 161375^*) and five R65aa (as in *Pm-w^WMR 29^*) in 26 melon accessions selected for their contrasted phenotypes for virus resistance triggered by two *A. gossypii* clones and resistance to two *P. xanthii* strains (Table S4). We retrieved or obtained by LR-PCR 33 complete genomic sequences, and we carried on manual annotations of CDS (Data S2). Two phylogenies were reconstructed based on protein alignments, one for Vat homologs with four R65aa and another for Vat homologs with five R65aa. The first phylogenic tree was looked at in relation with resistance to CMV triggered by aphids (Figure 5a) and the second in relation with resistance to PM (Figure 5b).

A *Vat* homolog with four R65aa was identified in the 20/26 lines tested (Table S4). The three lines without *Vat* homolog with four R65aa, Edisto 47, PI 124112, PI 282448 were susceptible to CMV inoculated by any aphid clones. The phylogenetic tree of the 21 proteins, rooted with a Vat homolog from *C. sativus*, highlighted two main clades (Figure 5a). The clade I contained the Vat homologs from all lines exhibiting resistance to CMV inoculated by NM1. This clade has evolved since 8.5 My (Figure S3). A sub-clade I contained 11 lines, which amplified the *Vat-1^PI 161375^* marker and the 11 Vat homologs shared at least 99.9% protein identity with Vat-1^PI 161375^. All lines, but CUM60, exhibited resistance to CMV when inoculated by NM1 and C9 aphids. We checked by Q-RT-PCR that *Vat-x^Cum60^* was expressed in no-infected CUM60 plants. Intriguingly, a single amino acid differentiated the Vat-x^Cum60^ from Vat-1^PI 161375^. This polymorphism was unique among the 21 other Vat homologs with four R65aa we studied. This amino acid, located in the LRR1 domain (position 605), might be involved in the recognition of the aphid effector of NM1 and C9 aphid clones. Nevertheless, the amino acid switched from glycine in PI 161375 to aspartic acid in CUM60, and its role in recognition should be checked. The second sub-clade I contained the *Vat* homologs from all lines exhibiting resistance to CMV inoculated by NM1 but susceptible to CMV inoculated by C9. The Clade II contained the Vat homologs from all lines exhibiting susceptibility to CMV inoculated by NM1 but PI 414723. Actually, PI 414723 contained two Vat homologs with four R65aa, Vat-3^PI 414 723^ in Clade II and Vat-1^PI 414723^ in Clade I. Therefore, resistance to CMV inoculated by aphids is likely related to *Vat-1* in PI 414723. Clade II exhibited two sub-clades, one gathering homologs from lines susceptible to CMV inoculated by both clones. The second sub-clade II contained two lines that exhibited resistance to CMV only when inoculated by C9 aphids.

A *Vat* homolog with five R65aa was present in the only 12/26 lines tested (Table S4). Among the lines without Vat homolog with five R65aa, some were susceptible to PM (PI 161375, PI 164323, Smith Perfect), whereas some were resistant to PM (PI 164723, PI 224770). These results illustrated that resistance to PM is complex and, aside *Pm-w* homologs, other loci are involved in the PM resistance in melon. The phylogenetic tree of the twelve proteins, rooted with a Vat homolog from *Cucumis sativus*, highlighted two main clades (Figure 5b). The clade I contained only lines resistant to PM, this clade has evolved since 8.5 My (Figure S3). Four lines shared homologs with proteins 100% identical to Pm-w*^WMR 29^* (Table S4)*,* which likely conferred the resistance to PM in Anso 77, Invernizo-8427, PI 124112, and Edisto 47. Two other lines exhibiting resistance to PM, PI 414723, and 9065, carried homologs coding for proteins at least 90% identical to Pm-w^WMR 29^ (and 92 or 94% between them).

The common ancestor to *Vat* homologs with five R65aa belonging to the clade I has evolved to confer PM resistance, while the common ancestor to *Vat* homologs with four R65aa belonging to the clade I has evolved to confer at least NM1 aphid resistance. Interestingly, seven accessions possessed at least two *Vat* homologs, one with four R65aa and another with five R65aa and four of them (PI 414723, 90 625, Anso 77, and Invernizo-8427), combined resistance to PM due to *Pm-w* and resistance to CMV when inoculated by *A. gossypii*.

## Discussion

Plants are continuously under pest and pathogen attacks. To survive, they have developed a sophisticated innate immune system capable of identifying and neutralizing aggressors, while resistance gene duplications is a common way to increase their repertoire [31]. The NLR gene number varies greatly between plant species and a high proportion of them are present in clusters. Some crops, such as hot pepper, apple, and wheat, reportedly have around 1000 NLRs [2], while the number of NLRs in the *Cucurbitaceae* family is consistently lower, ranging from 50 to 100, depending on the species ( [28];). To date, a few CC-NLR resistance genes have been isolated in melon targeting pathogens, such as *Fusarium* wilt, viruses, and aphids [1, 9, 17].

### *Vat homologs* confer resistance to powdery mildew and to *A. gossypii* aphids

**Figure 6:**
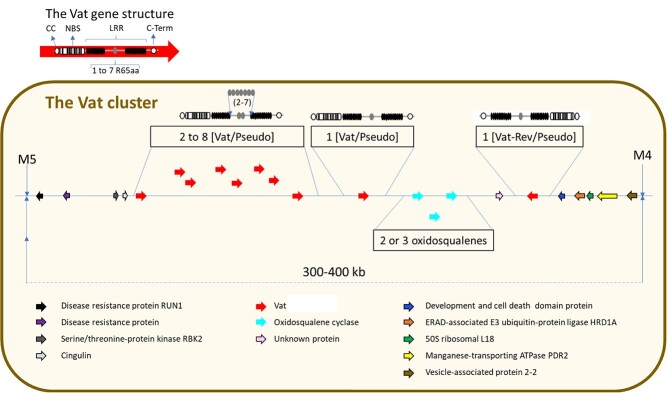
Schematic structure of the *Vat* cluster in *Cucumis melo.* The *Vat* cluster delimited by the M5-M4 markers and localized at the end of the chromosome 5 contained two to nine *Vat-x* or *Pseudo-Vat-x* and one *Vat-Rev* or *Pseudo-VatRev* and two or three oxidosqualene genes. Each *Vat homolog* contained one to seven repeats of a conserved motif of 65 amino acids (R65aa) in their LRR2 domain.

In this study, we revealed the presence of four functional CC-NLR genes, homologous to the *Vat* gene, in the powdery mildew resistant WMR 29 melon line. We isolated the *Pm-w^WMR 29^* gene using a positional cloning approach and showed, by stable complementation experiments in a susceptible melon line, that it conferred high resistance to *P. xanthii* race 1 and partial resistance to *P. xanthii* race 3*,* but did not confer neither resistance to *A. gossypii* nor resistance to CMV inoculated by this aphid. Conversely, we showed that its allele in PI 161375 (*Vat-1^PI 161375^*) did not confer resistance to PM while it has been reported conferring resistance to *A. gossypii* and resistance to CMV inoculated by this aphid [17]. Actually, R-genes diversity has been increasing in plant species and lines carrying allelic forms target different pathogen strains. For instance, in wheat, the *Pm3* gene perfectly illustrates the diversity of allelic forms targeting a large range of PM strains [6]; in flax, alleles at the L locus encode resistance to *Melampsora lini* races [19], and in *Arabidopsis thaliana*, RPP13 alleles encode resistance to *Peronospora parasitica* races [7]. Alleles of a single NLR-gene can also confer resistance to distinct pathogens. In different *A. thaliana* ecotypes, the RCY1 gene conferring resistance to the yellow cucumber mosaic virus strain is allelic to the HRT gene conferring resistance to the *Tombusviridae* turnip crinkle virus, and to the RPP8 gene conferring resistance to the *Peronospora parasitica* oomycete, responsible for downy mildew [42]. This three-allele locus coexists with clusters of R genes of the same family derived from complex duplications and rearrangements in the *Arabidopsis* genome [29]. A similar situation may exist in tomato, where it has been shown that the dominant powdery mildew resistance genes *Ol-4* and *Ol-6* are homologous of the CC-NLR *Mi-1* gene, which confers resistance to nematodes, whiteflies, and aphids, but their allelic status has yet to be clearly established [40]. Novel NLR functional diversity may occur through unequal crossover or gene conversion may occur when NLRs are clustered, with both mechanisms being facilitated by the repetitive structure of LRR coding sequences [4]. In melon, the *Vat* cluster exhibits explosive CC-NLR diversification with 60 *Vat* homologs described in thirty lines. Each line contains at least three, i.e., PI 161375, and up to five, i.e., Anso 77, functional *Vat* homologs and up to six non-functional pseudo-*Vat,* i.e., Charentais Mono, within a 300- to 400-kb region [13]. We illustrated this diversity in the canonical structure of the *Vat* cluster shown in Figure 6.

*Pm-w^WMR 29^* and *Vat-1^PI 161375^* are allelic in the PI 161375 and WMR 29 lines because they are located in the same genetic interval and share the first position in the series of *Vat* homologs within the cluster (Figure 2). Nevertheless, *Vat-3^Anso 77^*, which shares 100% identity with *Pm-w^WMR29^*, is in the third position in the homolog series in PM-resistant Anso 77 [13]. Actually, the first position in the homolog series has been shown to be shared by *Vat* homologs with three (Doublon, DHL92, Harukei-3), four (PI 161375, Anso 77), five (WMR 29, HS), and seven (Payzawat) R65aa, a conserved motif of 65 amino acids in the LRR domain, in melon diversity [13]. The absence of correlation noted between the repeat divergence and position might suggest that the repeat number variation was mainly generated by unequal crossover rather than by tandem duplication arising from a common ancient ancestor, as is the case for R gene diversity in *Solanum pimpinellifolium* [11]. Finally, rearrangements are highly frequent in the *Vat* cluster within melon diversity and the function of individual *Vat* homologs cannot be inferred from their position in the cluster.

### An ancestor of *Vat* homologs with four R65aa evolved toward aphid resistance while an ancestor with five R65aa evolved toward PM

Molecular determinants of the interaction specificity are essential to gain insight into plant resistance and pathogen virulence. We previously showed that *Vat*-mediated defense responses are effective against aphids and viruses [8, 17], and we have now extended the locus function to a fungus. It is now essential to understand how the specific recognition of organisms as different as aphids and powdery mildew takes place.

Two homologs have been cloned, *Vat-1^PI 161375^* conferring resistance to aphids and no resistance to PM, and *Pm-w^WMR 29^* conferring resistance to PM and no resistance to aphids. The most remarkable difference between these homologs was their number of R65aa in the LRR domain: four in *Vat-1^PI161375^* and five in *Pm-w^WMR 29^*, raising questions about the role of R65aa number in resistance specificity. The LRR domain has a major role in pathogen recognition [43], and in particular, the number of repeat motifs as shown in the tomato-*Cladosporium fulvum* pathosystem [45]. Remarkably, phylogeny of homologs with four R65aa repeats was congruent with resistance to CMV inoculated by aphids while phylogeny of homologs with five R65aa repeats was congruent with resistance to PM phenotype. This suggested that, in the *Vat* cluster, a homolog with four R65aa is needed for resistance to *A. gossypii* while a homolog with five R65aa is needed for resistance to PM*.* These homologs derived from ancestors about 8.6 Mya old*.* The “Vat-cluster” in *C. sativus* contains only one *Vat* homolog but an explosive diversification occurred in *C. melo* [13] concomitant to *C. sativus* and *C. melo* divergence estimated between 6.9 and 10.2 Mya (Timetree.org). Homologs with four R65aa have evolved early towards resistance to aphids (about 7 Mya, Figure S3) while susceptibility appeared later (about 3.9 Mya). In contrast homologs with five R65aa have evolved concomitantly towards resistance and susceptibility to PM (about 6.8–7.7 Mya). Interestingly, this has allowed emergence of Vat clusters containing a pair of homologs with four and five R65aa which might confer resistance to aphids and PM (see Tables S2 and S3).

The function of many *Vat* homologs remains to be deciphered. The use of CRISPR mutagenesis targeting only one homolog within the *Vat* cluster is today impossible to implement due to the strong identity between the numerous *Vat* homologs within a same line: no specific gRNA can be designed for each. Hence, positional cloning and functional complementation experiments may be requested to assign to *Vat* homolog functions, as we achieved in this paper for *Pm-w^WMR 29^* and previously for *Vat-1^PI 161375^* [17].

### Frequency of *Vat-1*^*PI 161375*^ and *Pm-w*^*WMR 29*^ in melon diversity

Specific *Vat-1^PI 161375^* and *Pm-w^WMR 29^* markers were present in 10% to 13% of 678 accessions representative of most wild and cultivated melon types worldwide. Interestingly, we identified these markers in 14 wild accessions mainly from India, suggesting they existed before domestication in the primary centre of melon diversity [23]. However, we cannot rule out that these wild resistant accessions resulted from more recent pollen fluxes or that they are feral types. The *Vat-1^PI 161375^* marker produced four false positives (SVI 0023, WM 7, Baza and CUM 60) for resistance to *A. gossypii* among 52 lines phenotyped (Table S3), while the *Pm-w^WMR 29^* marker produced onefalse positive (Casca de Carvalho) among 21 lines phenotyped for *P. xanthii* resistance (Table S2), therefore both markers inflated the resistance frequency inferences. The *Vat-1^PI 161375^* marker revealed *Vat* homologs sharing at least 99% identity between them and likely conferring virus resistance triggered by NM1 and C9 aphid clones (Table S4). Nevertheless, as suggested above, other *Vat* homologs might confer resistance triggered by *A. gossypii* and therefore the *Vat-1^PI 161375^* marker would have underestimated the frequency of *Vat-*resistance in melon diversity. The *Pm-w^WMR 29^* marker revealed homologous genes with 100% identity (Table S4). In melon, several other loci are known to be involved in PM resistance such as those located on chromosomes 2, 8, 12 [33, 34, 47] and on chromosomes 10 and 12 [16] which may explain the *P. xanthi* resistance, in accessions for which no *Vat* homolog with five R65aa was detected. Moreover, some of these QTLs are probably present in WMR 29, allowing to this accession to reach a complete resistance to races 1 and 3.

## Conclusion

The *Vat/Pm-w* locus constitutes a valuable model of multi-homolog genes for research on plant recognition and mechanisms of interaction with very different pathogens, such as aphids and fungi. Here, we demonstrated that two homologs of a CC-NLR gene triggered resistance to specific aphid clones and also to specific powdery mildew races. The complexity of the large cluster comprising the *Vat/Pm-w* genes and its diversity in several melon genotypes suggested that *Vat* homologs may contribute to the recognition of a broad range of pests and pathogens that remains to be discovered.

### Experimental procedures

#### Fine mapping of the *Pm-w*^*WMR 29*^ locus and sequencing

High resolution genetic mapping of *Pm-w*^***WMR 29***^ was conducted in 3760 BC individuals of a cross between the PM-susceptible cultivar Védrantais (Vilmorin release) and the PM-resistant cultivar WMR 29 [F1 (Védrantais x WMR 29) x Védrantais]. The markers used have been previously described [17] and are listed in Table S1a.

A BAC genomic DNA library was constructed from WMR 29 plants, using the *Escherichia coli* DH10B strain and the pIndigoBAC5 vector. The WMR 29 library represents about seven genome equivalents (33 000 clones) and has an average insert size of 100 kb. The BAC library was screened with L273 and P851 markers flanking *Pm-w^WMR 29^*. Six 100–145 kb BAC clones containing *Pm-w^WMR 29^* were found. A 33 kb subclone was selected, that comprised both flanking markers. The C2 subclone bearing the *Pm-w^WMR 29^* locus was sequenced by shotgun sequencing. Sequency assembly was performed using the Phred and Phrap software package [20]. Structural annotation was conducted using AUGUSTUS (https://bioinf.uni-greifswald.de/augustus/) and FGENESH gene prediction (http://www.softberry.com) and predicted genes were functionally annotated by InterProScan (https://www.ebi.ac.uk/interpro). The 33 kb genomic sequences were exported to the CLC Main Workbench (https://resources.qiagenbioinformatics.com) for expert manual annotation of *Vat-1^WMR 29^* by homology with *Vat-1^PI 161 375^*.

### Detecting the number of *Vat* homologs present in the cluster of different melon accessions and the number of R65aa in their LRR2 domain

Three pairs of previously described primers [13] were used for PCR (Table S1b). The Z649F/R primers were located in introns on either side of exon 2, spanning R65aa motifs in the LRR2 part, and amplified the majority of *Vat* homologs containing 2, 3, 4, or 5 R65aa motifs. Band numbers observed via gel electrophoresis for each accession corresponded to the minimal number of *Vat* homologs present in the cluster; band sizes allowed us to infer the number of R65aa motifs in these *Vat* homologs, at least for homologs with more than one R65aa. Because of their divergence from other *Vat* sequences, Z6097F/Z6095R-specific primers located in exon 2 were designed to detect *Vat* homologs with a single R65aa, producing a 467-bp amplicon in WMR 29. Similarly, the Z5474F/R located at the end of exon 1 was designed to partly amplify pseudo*-Vat-Rev* homologs, producing a 941-bp amplicon in WMR 29.

### Sequencing of *Vat* homologs in different melon accessions

DNA sequences of *Vat* homologs were recovered from 21 melon accessions (Table S4). Sequence amplification was obtained by long-range PCR with different primer pairs (Z717F/R, Z6473F/Z717R, Z5263F/Z5184R, Z5469F/R) located in the 5′ and 3′ UTR regions (Table S1b), using the ExTaq Hot start enzyme (Takara BIO Inc, Japan, www.takara-bio.com) and under the thermal cycling conditions (94°C for 4 minutes, 35 cycles at [94°C for 30 seconds; 59°C for 30 seconds; 72°C for 4 minutes 30 seconds] and 72°C for 10 minutes). PCR products were cloned using the pGEM®-T Easy Vector System (Promega, Madison, WI, USA) and plasmid extraction was performed using the QIAprep Spin Miniprep Kit (Qiagen, Germany) according to the manufacturer's protocols. One to two clones per *Vat* homolog were sequenced. Nucleic and protein sequence identities were obtained using Clustal Omega sequence alignment tools (https://www.ebi.ac.uk/Tools/msa/clustalo/).

### Transcription analysis and producing CDS

Total RNA extraction, first-strand cDNA synthesis, and RT-PCR were performed as described previously [13]. For *Vat-1^WMR 29^* full cDNA was amplified by long-range PCR with the Z717F/R primers respectively located in the 5′ and 3′regions of the gene (Table S1b), using the same conditions as above. The final RT-PCR product (4968 bp) was cloned in the pGEM®-T Easy Vector System (www.promega.com) and plasmid extraction was carried out using the QIAprep Spin-Miniprep Kit (QIAGEN, www.qiagen.com). Full cDNA was sequenced using SANGER technology (Genoscreen, www.genoscreen.fr) with a set of primers and manually assembled or with CAP3 (https://doua.prabi.fr/software/cap3). For other *Vat* homologs present in WMR 29 and in all other genotypes studied, specific LR-PCR primers were designed (Table S1b) and partial cDNAs spanning all exon junctions, were amplified by RT-PCR and directly sequenced (without cloning) using SANGER technology. Their expression was confirmed by RT-PCR (Z5071F/Z5069R, Z5951F/R, Z5895F/R, Z5471F/R) (Table S1b).

### Phylogenetic reconstruction

All evolutionary analyses were conducted in MEGA X [27]. Vat homolog proteins coded by 23 *Vat* homolog CDS with four R65aa from melon lines and from a CDS with one R65aa from *C. sativus* cv 9930 (retrieved from [13]) were aligned by parts ([CC_NBS_LLR1]-[LRR2]-[LRR3-C-Term] to respect the *Vat* gene structure. The evolutionary history was inferred by using the Maximum Likelihood method and Jones et al. w/freq. Model in MEGA X. Initial tree(s) for the heuristic search were obtained automatically by applying Neighbor-Join and BioNJ algorithms to a matrix of pairwise distances estimated using the JTT model, and then selecting the topology with superior log likelihood value. A discrete Gamma distribution was used to model evolutionary rate differences among sites (five categories (+*G*, parameter = 0.4111)). The tree is drawn to scale, with branch lengths measured in the number of substitutions per site. There were a total of 1648 positions in the final dataset.

Vat homolog proteins coded by twelve *Vat* homolog CDS with five R65aa from melon lines and from the CDS with one R65aa from *C. sativus* cv 9930 were aligned and analyzed as above (+*G*, parameter = 0.3599). There were a total of 1705 positions in the final dataset.

The time trees were computed using 6.9 to 10.2 Mya divergence time constraint between *C. melo* and *C. sativus*. Védrantais was used in calibration of both trees. The Tao method was used to set minimum and maximum time boundaries on nodes for which calibration densities were provided.

### Definition of *Vat-1*^*PI 161 375*^ and *Pm-w*^*WMR 29*^ specific markers

Multiple sequence alignment (https://www.ebi.ac.uk/Tools/msa/clustalo/) was performed with genomic and CDS sequences of *Vat* homologs obtained from PI 161375 and WMR 29 and a specific primers pair was designed (https://bioinfo.ut.ee/primer3-0.4.0/) to amplify *Pm-w^WMR 29^* (Z5071F/Z5069R; Table S1b). To amplify *Vat-1^PI 161375^*, the marker (Z1431F/R) previously described was used. The frequency of both specific markers was investigated by PCR on 678 melon accessions from 29 countries including wild and domesticated melons, selected from the collection maintained by the INRAE Centre for Vegetable Germplasm in Avignon [39]. Five to 10 seedlings of each accession were grown in a glasshouse. Leaf samples from 2-week-old seedlings were pooled and DNA was extracted as previously described [17].

### Complementation analysis

For complementation experiments, an 11-kb insert of melon genomic DNA, including the *Vat-1^WMR 29^* sequence and 2.2 and 2.4 kb from the 5′ and 3′ regions, respectively, was amplified by long-range PCR (Z2412F/R; Table S1b) and cloned into the pBIN 19 binary vector using the In-Fusion™ Advantage-PCR-Cloning Kit (https://www.takarabio.com). The resulting plasmid contained the selectable marker neomycin phosphotransferase gene (*NptII)* conferring kanamycin resistance. After transfer in DH10Bα *E. coli*, plasmid extraction was performed using the QIAprep Spin-Miniprep Kit and *Agrobacterium tumefaciens* transformation was obtained by electroporation into two C58 strains containing different helper plasmids (PGV2260 or Pch32). Melon transformation was performed as previously described [12] from young leaf explants of the susceptible Védrantais line. Integration of the entire T-DNA binary plasmid into the transgenic plants was determined by PCR with primers (SPE115F/R) specific to the *NptII* gene and primers (Z649F/R) amplifying a 1472 bp product corresponding to the second exon of the *Vat-1^WMR 29^* gene (Table S1b).

Segregation of the T-DNA binary plasmid in T1 progenies was studied to determine whether a single insertion of the transgene had occurred (Table S5).

### *Vat-1* ^*WMR 29*^ expression analysis in transgenic plants by Q-RT-PCR

The four transgenic melon lines (PmW3, PmW4, Pm7, PmW18) and the non-transgenic resistant control (WMR 29) underwent *Vat-1^WMR 29^* expression analysis. RNA extraction, reverse-transcription, candidate reference gene selection and primer design were conducted according to the MIQE guidelines [10]. Specific Q299F and Q299R markers located in *Vat-1^WMR 29^* exon 3 and amplifying a 212-bp amplicon were designed (Table S1b). Three stable reference genes, i.e. *TIP41*, *actin,* and *TUA* [3] were selected using GENORM software to build an accurate normalization factor for measuring the *Vat-1^WMR 29^* gene expression by qPCR using the SYBR Green Chemistry method. The data were analyzed using the R package RqPCR analysis script [24]. Further details on the experimental design are given in the Supplemental methods.

### Powdery mildew resistance assay

Two monoconidial isolates of *P. xanthii*, Sm3 (race 1) and 00Sm39 (race 3) were used for powdery mildew resistance tests and a set of four melon reference genotypes (WMR 29, Edisto 47, PMR 45, PI 124112) was used to confirm the race of each fungal strain [36]. Four to five weeks post-sowing, leaf disks (20 mm of diameter) were taken from expanded melon leaves and placed face upwards on agar medium (4 g∙L^−1^) in plastic boxes containing 54 discs), with two discs of the four reference genotypes. Each box was placed under a settling tower and inoculation was performed by blowing conidia present on an infected *Lagenaria siceraria* cotyledon. The boxes were then maintained in a growth chamber at 24/18°C under a 16-hour photoperiod at 40 μmol∙s^−1^∙m^−2^. Mycelium colonization and sporulation intensity were estimated post inoculation (DPI) using visual notation under a binocular magnifier on a 0 to 9.

A first series of four experiments were carried out (one by Pm-W transgenic line). Thirty-two leaf discs of Védrantais, WMR29, and a transgenic line were inoculated and scored ten days post inoculation. Four Steel-Dwass-Critchlow-Fligner / bilatéral tests (Addinsoft® XLStat software v20.13.4.06) were carried to compare Védrantais, transgenic line and WMR29 scores.

A second series of two experiments were carried out (one by PM race) with two transgenic lines, Védrantais and WMR29. Fifty leaf discs per of each transgenic line and eighty discs from Védrantais and WMR29 were inoculated and scored daily from 6 to 14 days. The areas under the curve were calculate for each disc and ANOVA and Bonferoni pair test (*p* < 0.05) (Addinsoft® XLStat software v20.13.4.06) were carried out to compare the four genotypes for areas under the curve.

### Aphid resistance assay

Resistance tests with aphids were performed on 10 to 20 plants per lines, as previously described [8] using the NM1 and C9 *A. gossypii* clones. To assess *A. gossypii* ability to colonize melon plants, 10 adult aphids were deposited on 10 (for the reference lines Védrantais and PI 161375) or 15 plants (for the PmW4 and PmW7 transgenic lines). Seven days later, the adults were then counted, and the density of nymphs was estimated on a scale from 0 to 6. The aphid colonization parameter was calculated as [density of nymphs + ln (number of adults +0.01)].

Resistance to virus when inoculated by aphids was assessed with the NM1 or C9 *A. gossypii* Aphids from mass rearing were transferred to CMV (isolate I17F) infected leaves of Védrantais for 10 minutes virus acquisition and 10 viruliferous aphids were then deposited on plants for 15 minutes virus inoculation. Plants were scored resistant or susceptible 15 days later.

## Supplementary Material

Web_Material_uhad256
