# Supplementary material for: A highly diversified NLR cluster in melon contains homologs that confer powdery mildew and aphid resistance
Source: Hortic Res. 2023 Dec 13;11(1):uhad256. doi: 10.1093/hr/uhad256 (PMC10807702; doi:10.1093/hr/uhad256)
Supplement: Web_Material_uhad256 [file web_material_uhad256.zip › 3-Additional supporting information R2bis.docx]

**ADDITIONAL SUPPORTING INFORMATION**

**Table S1. Primer sequences used in this study**

1. **PCR-based markers linked to the *Pm-w* locus**. F: forward and R: reverse.

| Primer name | Primer Sequence (5’-3’) |
| --- | --- |
| M1 | F: GTGTATTTTGTGGGTGAACCAAAG /////////////////////// R: GAAATTGGAGCGAGTATCTTCTCC |
| M4 | F: GGGCTGACAGTTAGAGTATCCAC ///////////////////////// R: TAATAACATGAAACACTCTGTCAATTT |
| M5 | F: TTAACGCTCTTTGTTCTAAGCTTTTGTA///////////////// R: CAGCCATTGTATGATGCAAATGACCAA |
| M8 | F: CCGACGCATCTCCCGACGCGTTGTTG /////////////////// R: TCGTGAAGGGTTTTGGAGAGTGAGAAA |
| L246 ^x^ | F: AATG[A/G]ACAACCACATAAACAATGAAAC //////// R: GCAATCAAACAACAATCAATATCATCG |
| L273 ^x^ | F: GGAGAGAGAATCCGGGACTAAGTGACT ////////////// R: TAACCACCTTTTCCGATCAAATTTTCTAC |
| R273 | F: TTGTTACGATCATGAAACACATTGC ///////////////////// R: TGATAATACTGCATTTACTAGGAGTG |
| V432 ^x^ | F: TTAGAGTGGCAAAGGGAAGATGGG ///////////////////// R: AACTTCTCCAACTC[-/C]CTCCACTGC |
| V681 | F: GGAATCTTGTTGAGGCCGAGAGGG ////////////////////// R: GTTGTATATGGCTTCCCTGTAGCC |
| V1684 ^x^ | F: CAACAGGCTCAACAGTGTATTC[A/G]G ///////////////// R: GAAGAAGGTGACGAGAGAGATGCC |
| P851^x^ | F: TAATAATGCCTACACACACATATCC ///////////////////// R: GTAAGAGTACGAATTGTTCACAACC |
| GRP805 | F: ATCCCCTGTTTCCTTCAACAACCC //////////////////////// R: AACCCCCAAGAAGAAGAACAACCC |
| L154 | F: GCATCATGAAGTTGGAATGACGAATGGA //////////// R: AAATCGTCTGGCCCGTGACTTTTGTTG |

^x^ SNPs between [WMR 29 / PI 161375]

**(b) Primer sequences designed on *Pm-w* and *Vat*-homologs sequences, location and amplicon lengths**

| Primer name | Primer Sequence (5’to3’) | Location | Amplicon  length (bp) |
| --- | --- | --- | --- |
| Z2412 | F: CTTCTAACTCTTCTCTCTTCCTCGCAAA  R: GATTGTTTCAGCAAGCTAAGAGACCAAC | *Pm-w^WMR 29^* promotor  *Pm-w ^WMR 29^* terminator | *Pm-w^WMR 29^* : 10624 |
| Z5071  Z5069 | F: CCTTAAAGTCTTAGAGATTCGAGA  R: GCATGAACAAATTCTTATTTCCTCC | *Pm-w ^WMR 29^* specific markers (exon 2) | *Pm-w^WMR 29^* : 337 |
| Q299 | F: GCAACTTGAACATTTGAGAATACAC  R: GACAGTCCCAAATGTATACTTTCCT | *Pm-w ^WMR 29^* specific markers (exon 3) | *Pm-w^WMR 29^* : 212 |
| Z717* | F: CTCTGCACTGTCTCTTCTCCATTTC  R: CAAGA[G/T]TCTGACCTTTTCCTTGTGG | Common LR-PCR markers (5’-3’ UTR) | *Pm-w^WMR 29^* *:* 6441  *Vat-3rep^WMR 29^*: 6091 |
| Z6473  Z717* | F : TGGTTCAGTCCCTGCGGGTT  R: CAAGA[G/T]TCTGACCTTTTCCTTGTGG | *Vat-4rep^WMR 29^* LR-PCR markers  (5’-3’ UTR) | *Vat-4rep^WMR 29^*: 6679 |
| Z5263  Z5184 | F: ATGTTCCTCACGGGGGTTTATGCCAAT  R: CTTGGGGATGAAATGATATAAAGTG | *Vat-1rep^WMR 29^*  LR-PCR markers  (5’-3’ UTR) | *Vat-1rep^WMR 29^*: 11000 |
| Z5469 | F: GGTTTCAAGAATCTGACCTTCC  R: ATGATTATGGTTCATTCTTTTTGGG | *Vat-Rev^WMR 29^*  LR-PCR markers  (5’-3’ UTR) | *Vat-Rev^WMR 29^*: 5412 |
| Z649 | F: TGTCACAAACTGAACTTTTAAGGA  R: CTGTTCAACTAACAGAACCAATTC | Common *Vat* markers (intron 1 – intron 2) | *Pm-w^WMR 29^* : 1472  *Vat-x^WMR 29^* with 4 R65aa: 1274  *Vat-y^WMR 29^* with 3 R65aa: 1086 |
| Z6097  Z6095 | F: TGAAAATTAGGAGCGCAACGAA  R: CTTTTTCTTCCATATAACCTCCACA | *Vat-1rep* specific markers (exon 2) | *Vat-1rep^WMR 29^*: 467 |
| Z5474 | F: ATGTGGTTGGTTGCCTTCTC  R: CGTTTACGGGGATGTGATTT | *Vat-Rev* specific marker (exon 1 - intron 1) | *Vat-Rev^WMR 29^*: 941 |
| Z5751 | F: GGAATCAAAGCAATGGGAGA  R: GGAACAAGAAGTAATGCTTAAATTGC | RT-PCR : *Vat-4rep^WMR 29^*  (exon 2 – exon 3) | *Vat-4rep^WMR 29^*: 616 |
| Z5951 | F: CAAATTAAAGTATACTTTTGGACCCACA  R: CTTTTGCATTCATTTATTGTCAAATCTTG | RT-PCR : *Vat-3rep^WMR 29^*  (exon 1 – exon 3) | *Vat-3rep^WMR 29^:* |
| Z5895 | F: GCCAATTGACACAACTCAAAGTGTTA  R: CATATCATCTTCAAATTCGTTGCGC | RT-PCR : *Vat-1rep^WMR 29^*  (exon 1 – exon 2) | *Vat-1rep^WMR 29^*: 1042 |
| Z5471 | F: TTGAAGCGATGGAAGGATTC  R: GGGAAGAAAAAGTTGGCAGA | RT-PCR : *Vat-Rev^WMR 29^*  (exon 2 – exon 3) | *Vat-Rev^WMR 29^*: 526 |
| Z1431 | F: ATGCAAAGAGTTTGAAGATG  R: GAAGATTACATAACATCAACGA | *Vat-1^PI161375^* specific markers (exon 2) | *Vat-1^PI161375^:* 858 |
| SPE115 | F: CTTCAGCAATATCACGGGTAGC  R: CTCCTGCCG AGAAAGTATCC | *NpTII* | 400 |
| SPE82 | F: CGATGGCCCACTACGTGAAC  R: TCGCCTTGCAGCACATCC | pBin19 left T-DNA border | 257 |

^x^ SNPs between [WMR 29 / PI 161375]

**(c) Primer sequences and amplicon lengths for 4 candidate reference genes and the *Pm-w* gene.**

Amplification efficiency (*E*) and coefficient of correlation (*R^2^*) of primers pairs were calculated with the MxPro qPCR software

| Gene | Melon unigene^1^ | Primer sequence | Amplicon length (bp) | *E*  *(1+eff)* | *R^2^* |
| --- | --- | --- | --- | --- | --- |
| *Profilin* | MU43545^1^ | F: ATTATCTTATTGATCAGGGCCTCTAAA^2^  R: TCGTATAAGCAATGAGAGAAATCTGG | 102 | 2.000 | 1.000 |
| *Actin* | MU43164^1^ | F: ATGGAAGCTGCAGGAATCCAC  R: CCACCACTGAGGACGATGTTTC | 101 | 1.957 | 1.000 |
| *TUA* | MU45968^1^ | F: ACTACCAGCCACCCACTGTT  R: ATGCGAGAGAAGACCTCAGC | 106 | 1.980 | 0.998 |
| *TIP41* | MU47705^1^ | F: GCCAAGTGGTTGGTTTCTTC  R: CGTAGCCTCTCTCCAGCAAC | 145 | 2.029 | 0.999 |
| *Pm-W* | Q299 | F: GCAACTTGAACATTTGAGAATACAC  R: GACAGTCCCAAATGTATACTTTCCT | 212 | 1.955 | 1.000 |

^1^ available at <http://icugi.org>

² from Anstead et al. (2010) *Entomologia Experimentalis et Applicata* **134**, 170–181

**Table S2. Frequency and geographical distribution of the *Pm-w^WMR 29^* marker in melon and relationship with powdery mildew resistance**

Screening of a collection of 678 melon accessions from different botanical groups and worldwide origin: 73 accessions amplified the *Pm-w^WMR 29^* specific marker (Z5071F/Z5069R). In grey, 15 accessions, which also amplified the *Vat-1^PI161375^* specific marker (Z1431F/R).

Powdery mildew phenotyping: (R) resistant, (I) intermediate, (S) susceptible, (nt) not tested

| **Accessions** | **Botanical Group^1^** | **Origin Country** | **Geographic Region** | ***P. xanthii* race 1 (Sm3)** | ***P. xanthii* race 3 (00Sm39)** |
| --- | --- | --- | --- | --- | --- |
| EDISTO 47 | Cantalupensis | USA | Northern America | **R^2^** | **R^2^** |
| WMR 29 | Cantalupensis | USA | Northern America | **R** | **R** |
| SEMINOLE | Cantalupensis | USA | Northern America | **R** | - |
| CAIPIRA | Cantalupensis | Brazil | South America | **R** | **I** |
| CAIPIRA 224 | Cantalupensis | Brazil | South America | **R** | **I** |
| CASCA DE CARVALHO | Unknown | Brazil | South America | S | S |
| CONCHAS | Cantalupensis | Brazil | South America | **R** | **R** |
| GAUCHO COMPRIDO | Cantalupensis | Brazil | South America | **R** | **I** |
| GAUCHO FELTRIN | Cantalupensis | Brazil | South America | S | S |
| GAUCHO ISLA | Cantalupensis | Brazil | South America | **R** | **I** |
| GAUCHO REDONDO | Cantalupensis | Brazil | South America | **R** | **I** |
| IVAI | Cantalupensis | Brazil | South America | **R** | **I** |
| FEGOUSS 1 | Flexuosus | Morocco | Northern Africa | **I** | **I** |
| PI 234607 | Chandalak | South Africa | Southern Africa | **R^3^** | - |
| MR-1 | Momordica | India | Southern Asia | **R^4^** | **R^4^** |
| PI 124112 | Unknown | India | Southern Asia | **R^5^** | **R^5^** |
| ANSO 77 | Ibericus | Spain | Southern Europe | **R** | **R** |
| INVERNIZO 8427 | Ibericus | Spain | Southern Europe | **R** | **I** |
| MALAGA SE-2811 | Unknown | Spain | Southern Europe | **R** | **I** |
| NEGRO | Ibericus | Spain | Southern Europe | **R^6^** | - |
| 40099 | Cantalupensis | USA | Northern America | - | - |
| CAMPO | Cantalupensis | USA | Northern America | - | - |
| JACUMBA | Cantalupensis | USA | Northern America | - | - |
| MAINSTREAM | Cantalupensis | USA | Northern America | - | - |
| VA 435 | Cantalupensis | USA | Northern America | - | - |
| BOLA DE NEVE | Unknown | Brazil | South America | - | - |
| PI 293350 | Unknown | Peru | South America | - | - |
| CUM 412 | Unknown | Tunisia | Northern Africa | - | - |
| FAKOUS Marocco | Flexuosus | Morocco | Northern Africa | - | - |
| FQUS | Flexuosus | Tunisia | Northern Africa | - | - |
| PI 525143 | Unknown | Egypt | Northern Africa | - | - |
| SWEET MELON 72.7 | Unknown | Egypt | Northern Africa | - | - |
| ALIMIN 1 | Flexuosus | Sudan | Eastern Africa | - | - |
| GADAMBALIA 2 | Flexuosus | Sudan | Eastern Africa | - | - |
| HSD 2445 | Kachri | Sudan | Eastern Africa | - | - |
| HSD 2445-005 | Kachri | Sudan | Eastern Africa | - | - |
| MAURICE | Momordica | Maurice Island | Eastern Africa | - | - |
| MEDANI 70 | Unknown | Sudan | Eastern Africa | - | - |
| VOATANGO | Momordica | Madagascar | Eastern Africa | - | - |
| CUM 146 | Agrestis | Afghanistan | Central Asia | - | - |
| BEIJING 3 | Unknown | China | Eastern Asia | - | - |
| LANZHOU 15 | Makuwa | China | Eastern Asia | - | - |
| RED COTTON ROSE MELON | Ameri | China | Eastern Asia | - | - |
| TAINAN 8 | Inodorus | China | Eastern Asia | - | - |
| XICHANG 2 | Makuwa | China | Eastern Asia | - | - |
| ARYA 1 | Unknown | India | Southern Asia | - | - |
| INDE 2 | Unknown | India | Southern Asia | - | - |
| IVT 1976 | Unknown | India | Southern Asia | - | - |
| KEKIRI | Acidulus | Sri Lanka | Southern Asia | - | - |
| PI 124440 | Chandalak | India | Southern Asia | - | - |
| PI 163208 | Chandalak | Pakistan | Southern Asia | - | - |
| PI 164343 | Momordica | India | Southern Asia | - | - |
| PI 164856 | Chandalak | India | Southern Asia | - | - |
| PI 179914 | Unknown | India | Southern Asia | - | - |
| PI 180280 | Unknown | India | Southern Asia | - | - |
| PI 182950 | Unknown | India | Southern Asia | - | - |
| ALATUN | Ameri | Turkey | Western Asia | - | - |
| QARRËS I BODINAK (CUM 459) | Cantalupensis | Israel | Western Asia | - | - |
| DEIR EZZOR | Chandalak | Syria | Western Asia | - | - |
| EINDOR 1 | Unknown | Israel | Western Asia | - | - |
| HEMED | Cantalupensis | Israel | Western Asia | - | - |
| KHATONI | Ameri | Iran | Western Asia | - | - |
| NOY ISRAEL | Cantalupensis | Israel | Western Asia | - | - |
| QUEEN'S HONEY | Unknown | Israel | Western Asia | - | - |
| YOKNEAM 56 | Ameri | Israel | Western Asia | - | - |
| CHARENTAIS LAVERGNE | Cantalupensis | France | Western Europe | - | - |
| OLIVE D'HIVER | Ibericus | France | Western Europe | - | - |
| AL 18/143 | Unknown | Albania | Southern Europe | - | - |
| AL 18/144 | Unknown | Albania | Southern Europe | - | - |
| BULGARIE 7 | Cantalupensis | Bulgaria | Southern Europe | - | - |
| AMARILLO CANARIO | Ibericus | Spain | Southern Europe | - | - |
| AMARILLO CANARIO LISO | Unknown | Spain | Southern Europe | - | - |
| MALAGA AN-C-48 | Unknown | Spain | Southern Europe | - | - |

**-** : not tested

1. Botanical group following Pitrat, M. (2016) Melon genetic resources: phenotypic diversity and horticultural taxonomy. In *Genetics and Genomics of Cucurbitaceae*, (Grumet, R., Katzir, N. and Garcia-Mas, J. eds). Springer, pp 25-60
2. Ning et al. (2014) Inheritances and location of powdery mildew resistance gene in melon Edisto47. Euphytica 195, 345–353.

Hosoya et al., (2000) Impact of resistant melon cultivars on *Sphaerotheca fuliginea* Plant Breed, 119 (2000), pp. 286-288

1. McCreight et al. (2006) Melon-powdery mildew interactions reveal variation in melon cultigens and *Podosphaera xanthii* races 1 and 2 J Am Soc Hortic Sci, 131:59-65

Cui et al., (2022-a) Breeding melon (*Cucumis melo*) with resistance to powdery mildew and downy mildew. *Horticultural Plant Journal* **8**,545-561.

1. Li et al. (2017) Mapping of powdery mildew resistance genes in melon (*Cucumis melo* L.) by bulked segregant analysis Sci Hortic, 220 (2017), pp. 160-167
2. Epinat et al. (1992) Genetic analysis of resistance of five melon lines to powdery mildews Euphytica, 65 (1992), pp. 135-144

Perchepied et al. (2005) The powdery mildew fungus *Podosphaera xanthiii*, a constant threat to cucurbits: pathogen profile. Mol Plant Pathol, 10: 153-160

1. McCreight et al. (2006) Melon-powdery mildew interactions reveal variation inmelon cultigens and Podosphera xanthii races 1 and 2. J Amer Soc Hort Sci 131, 59-65.

**Table S3. Frequency and geographical distribution of the *Vat^PI 161375^* marker in melon and relationship with resistance to CMV inoculated by aphids**

1. Screening of a collection of 678 melon accessions from different botanical group and worldwide origin: 92 accessions amplified the *Vat* specific marker (Z1431F/R). In grey 15 accessions which also amplified the *Pm-w* specific marker (Z5071F/Z5069R).
2. Phenotyping for CMV transmission by two *Aphis gossypii* clones: (R) resistant, (I) intermediate, (S) susceptible.

| **Accessions** | **Botanical Group^1^** | **Origin Country** | **Geographic Region** | **CMV transmission by *A. gossypii*** | |  |
| --- | --- | --- | --- | --- | --- | --- |
|  |  |  |  | **NM1** | **C9** | |
| AR HALE'S BEST JUMBO | Cantalupensis | USA | Northern America | R | R | |
| MELONCILLO | Chito | Colombia | South America | R | R | |
| BOLA DE NEVE | Unknown | Brazil | South America | R | - | |
| CAIPIRA | Cantalupensis | Brazil | South America | R | - | |
| GAUCHO COMPRIDO | Cantalupensis | Brazil | South America | R | - | |
| GAUCHO FELTRIN | Cantalupensis | Brazil | South America | R | - | |
| GAUCHO ISLA | Cantalupensis | Brazil | South America | R | - | |
| CAIPIRA 224 | Cantalupensis | Brazil | South America | R | - | |
| CASCA DE CARVALHO | Unknown | Brazil | South America | R | - | |
| IVAI | Cantalupensis | Brazil | South America | R | - | |
| VOATANGO | Momordica | Madagascar | Eastern Africa | R | - | |
| MAURICE | Momordica | Maurice Island | Eastern Africa | R | - | |
| PI 482398 | Kachri | Zimbabwe | Eastern Africa | R | R | |
| PI 482420 | Acidulus | Zimbabwe | Eastern Africa | R | R | |
| CHINA 51 | Cantalupensis | China | Eastern Asia | R | R | |
| HYOUGO AO SHIMA URI | Conomon | Japan | Eastern Asia | R | - | |
| K 5442 | Makuwa | China | Eastern Asia | R | R | |
| KURONA URI | Makuwa | Japan | Eastern Asia | R | - | |
| LANZHOU 1 | Chinensis | China | Eastern Asia | I | R | |
| MIEL BLANC | Chinensis | China | Eastern Asia | R | R | |
| PI 161375 | Chinensis | Corea | Eastern Asia | R | R | |
| PI 255478 | Makuwa | Corea | Eastern Asia | R | R | |
| PI 266935 | Conomon | Japan | Eastern Asia | R | R | |
| SHIRO URI OKAYAMA | Conomon | Japan | Eastern Asia | R | R | |
| TIANSHUAI | Makuwa | China | Eastern Asia | R | - | |
| YULONG WHITE MELON | Makuwa | China | Eastern Asia | R | - | |
| KANRO MAKUWA 1 | Makuwa | Japan | Eastern Asia | R | R | |
| GINSEN MAKUWA | Makuwa | Japan | Eastern Asia | R | R | |
| SHIROKAWA NASHI MAKUWA | Makuwa | Japan | Eastern Asia | R | R | |
| CHENGGAM | Makuwa | Corea | Eastern Asia | R | R | |
| IC-267384 | Momordica | India | Southern Asia | I | - | |
| PI 164320 | Acidulus | India | Southern Asia | I | I | |
| RA CHIBBAR | Kachri | India | Southern Asia | I | R | |
| ARYA 2 | Flexuosus | India | Southern Asia | R | - | |
| DURGAPURA MADHU | Unknown | India | Southern Asia | R | R | |
| PI 123501 | Unknown | India | Southern Asia | R | - | |
| PI 164323 | Acidulus | India | Southern Asia | R | I | |
| PI 183311 | Kachri | India | Southern Asia | I | I | |
| PI 414723 | Momordica | India | Southern Asia | R | R | |
| IC-267363 | Momordica | India | Southern Asia | R | - | |
| SVI 0023 | Acidulus | India | Southern Asia | S | S | |
| WM 7 | Kachri | India | Southern Asia | S | - | |
| GAPAN | Unknown | Philippines | Southeastern Asia | R | - | |
| SAN ILDEFONSO | Unknown | Philippines | Southeastern Asia | R | R | |
| CHARENTAIS LAVERGNE | Cantalupensis | France | Western Europe | R | - | |
| CHARENTAIS Vat R | Cantalupensis | France | Western Europe | R | R | |
| MARGOT | Cantalupensis | France | Western Europe | R | R | |
| VIRGOS | Cantalupensis | France | Western Europe | R | R | |
| BAZA | Unknown | Spain | Southern Europe | S | - | |
| CUM 60 | Chito | Unknown | Unknown | S | S | |
| SVI 0105 | Kachri | Unknown | Unknown | R | R | |
| V001 | Cantalupensis | Unknown | Unknown | I | - | |
| AR PMR 5 (AR5) | Cantalupensis | USA | Northern America | - | - | |
| MELON CHIEN | Kachri | Guadeloupe | South America | - | - | |
| CONCHAS | Cantalupensis | Brazil | South America | - | - | |
| GAUCHO REDONDO | Cantalupenssi | Brazil | South America | - | - | |
| CUM 412 | Unknown | Tunisia | Northern Africa | - | - | |
| BOU RICHAH (CUM 196) | Ibericus | Tunisia | Northern Africa | - | - | |
| CUM 414 | Unknown | Tunisia | Northern Africa | - | - | |
| HSD 195 | Kachri | Sudan | Eastern Africa | - | - | |
| TIBISH 95-1 | Tibish | Sudan | Eastern Africa | - | - | |
| CUM 212 | Makuwa | China | Eastern Asia | - | - | |
| FUER | Makuwa | China | Eastern Asia | - | - | |
| HONG CHENG DONGLING | Makuwa | China | Eastern Asia | - | - | |
| LANZHOU 6 | Makuwa | China | Eastern Asia | - | - | |
| LONGTIAN | Makuwa | China | Eastern Asia | - | - | |
| MAKUWA 85.1601 | Conomon | Japan | Eastern Asia | - | - | |
| PI 093799 | Makuwa ? | China | Eastern Asia | - | - | |
| PI 093800 | Makuwa | China | Eastern Asia | - | - | |
| PI 136173 | Unknown | China | Eastern Asia | - | - | |
| PI 157080 | Makuwa | China | Eastern Asia | - | - | |
| TAINAN 7 | Unknown | China | Eastern Asia | - | - | |
| URUMQI 3 | Makuwa | China | Eastern Asia | - | - | |
| XIANGSUAI | Makuwa | China | Eastern Asia | - | - | |
| YANTAI 1 | Makuwa | China | Eastern Asia | - | - | |
| YANTAI 2 | Makuwa | China | Eastern Asia | - | - | |
| ARYA 1 | Flexuosus | India | Southern Asia | - | - | |
| INDE-5 | Flexuosus | India | Southern Asia | - | - | |
| PI 123823 | Momordica | India | Southern Asia | - | - | |
| PI 164487 | Unknown | India | Southern Asia | - | - | |
| PI 269474 | Unknown | Pakistan | Southern Asia | - | - | |
| SVI 0021 | Acidulus | India | Southern Asia | - | - | |
| SVI 0026 | Acidulus | India | Southern Asia | - | - | |
| WM 24 | Agrestis | India | Southern Asia | - | - | |
| WM 35 | Kachri | India | Southern Asia | - | - | |
| CANARI HATIF | Ibericus | France | Western Europe | - | - | |
| PASTIS 2 | Unknown | France | Western Europe | - | - | |
| PASTIS 3 | Unknown | France | Western Europe | - | - | |
| ZUNESCO 1 | Cantalupensis | France | Western Europe | - | - | |
| ISO ms-2 | Cantalupensis | Unknown | Unknown | - | - | |
| ISO ms-3 | Cantalupensis | Unknown | Unknown | - | - | |
| IVT 1816 | Kachri | Unknown | Unknown | - | - | |

^1^ Botanical group following Pitrat, M. (2016) Melon genetic resources: phenotypic diversity and horticultural taxonomy. In *Genetics and Genomics of Cucurbitaceae*, (Grumet, R., Katzir, N. and Garcia-Mas, J. eds). Springer, pp 25-60.

- : not tested

**Table S4. Comparison of *Vat*-homologs with four and five R65aa obtained from 26 melon lines and phenotypic responses to resistance tests**

| **Melon lines** | **Vat-x** | ***Vat-1^PI 161375^*** | | **CMV inoculataed by aphids^(3)^** | |  | **Vat-x** | ***Pm-w^WMR 29^*** | | ***P. xanthii* ^(5)^ race** | |
| --- | --- | --- | --- | --- | --- | --- | --- | --- | --- | --- | --- |
|  | **4 R65aa^(1)^** | **marker ^(2)^** | **Id. Prot** | **NM1** | **C9** |  | **5 R65aa^(1)^** | **marker ^(2)^** | **Id. Prot** | **1** | **3** |
| **Gaucho Isla** | *Vat-x^GauchoIsla^* | + | amplicon LRR2 100% | R | R |  | *Vat-y^GauchoIsla^* | + | amplicon LRR2 100% | nt | nt |
| **Voatango** | *Vat-x^Voatango^* | + | amplicon LRR2 100% | R | R |  | *Vat-y^Voatango^* | + | amplicon LRR2 100% | nt | nt |
| **PI 161375** | *Vat-1^PI 161375^* | + | 100.00 | R | R |  |  | - |  | S | nt |
| **PI 164323** | *Vat-x^PI 164323^* | + | 100.00 | R | I |  |  | - |  | S | I |
| **AM51** | *Vat-x^AM51^* | + | 99.93 | R | R |  |  | - |  | I | I |
| **San Ildefonso** | *Vat-x^San Ildefonso^* | + | 100.00 | R | R |  |  | - |  | nt | nt |
| **Ra chibbar** | *Vat-x^Rachib.^* | + | 100.00 | I | R |  |  | - |  | nt | nt |
| **Lanzhou_1** | *Vat-x^Lanzhou1^* | + | 100.00 | I | R |  |  | - |  | nt | nt |
| **PI 482398** | *Vat-x^PI 482398^* | + | 99.93 | R | R |  |  | - |  | nt | nt |
| **PI 414723** | *Vat-1^PI 414723^* | + | 99.93 | R | R |  | *Vat-4^PI 414723^* | - | 93.15 | R | I |
|  | *Vat-3^PI 414723^* |  | 87.12 |  |  |  | *Vat-5^PI 414723^* | - | 91.78 |  |  |
| **CUM60** | *Vat-x^Cum 60^* | + | 99.93 | S | S |  |  | - |  | nt | nt |
| **90625** | *Vat-x^90625^* | **-** | 92.50 | R | S |  | *Vat-y^90625^* | - | 90.54 | R | R |
| **WMR 29** | *Vat-x^WMR 29^* | **-** | 86.85 | S | S |  | *Pm-w^WMR 29^* | + | 100.00 | R | R |
| **Anso 77** | *Vat-1^Anso 77^* | **-** | 92.37 | R | S |  | *Vat-3^Anso 77^* | + | 100.00 | R | R |
| **Invernizo-8427** | *Vat-x^Invernizo^* | **-** | ns | R | S |  | *Vat-y^Invernizo^* | + | 100.00 | R | I |
| **PI 124112** |  | **-** |  | S | S |  | *Vat-x^PI 124112^* | + | 100.00 | R | R |
| **Edisto 47** |  | **-** |  | S | S |  | *Vat-x^Edisto 47^* | + | 100.00 | R | R |
| **PI 164723** | *Vat-x^PI 164723^* | **-** | 92.23 | R | S |  |  | - |  | R | R |
| **PI 224770** | *Vat-x^PI 224770^* | **-** | 92.01 | R | S |  |  | - |  | R | S |
| **Smith Perfect** | *Vat-x^SmithP.^* | **-** | 86.11 | S | R |  |  | - |  | S | S |
| **Canton** | *Vat-x^Canton^* | **-** | 86.11 | S | R |  |  | - |  | nt | nt |
| **PI 282448** |  | **-** |  | S | S |  | *Vat-x^PI 282448^* | - | 91.22 | S | S |
| **Cum 64** | *Vat-x^Cum64^* | **-** | 86.71 | **S** | nt |  | *Vat-y^Cum64^* | - | 87.43 | S | S |
| **Védrantais** | *Vat-3^Ved^* | **-** | 87.12 | S | S |  | *Vat-1^Ved^* | - | 87.88 | S | S |
| **Charentais mono** | *Vat-3^CharM.^* | **-** | 86.85 |  |  |  | *Vat-1^CharM^* | - | 87.94 |  |  |
| Charentais T |  | **-** |  | S | S |  |  | - |  | nt | nt |
| **DHL92** | *Vat-3^DHL92^* | **-** | 86.85 |  |  |  |  | - |  |  |  |
| Piel de sapo T111 |  | **-** |  | S | S |  |  | - |  | nt | nt |

1. The *Vat-x-*homolog genes with four or five R65aa detected by PCR with Z649F/R primers located in the LRR2 domain (Table S1b)

^(2)^ PCR amplification with Z1431F/R specific markers of *Vat-1^PI 161375^*

^(3)^ CMV-resistance triggered by three *Aphis gossypii* clones (NM1and C9)

1. PCR amplification with Z5071F/Z5069R specific markers of *Pm-w^WMR 29^*
2. Powdery mildew resistance assays conducted on melon lines with *Podosphaera xanthii* race 1 (Sm3) and race 3 (00Sm39)

R: resistant; S: susceptible; I: intermediate

ns: not sequenced;

nt: not tested

**Figure S1.** **Evaluation of resistance of two *Vat-1^WMR 29^*-Védrantais transgenic lines to aphids and** **CMV inoculated by aphids**

**(a) Assessment of the resistance to *Aphis gossypii* colonization.**

Test carried on according to (Boissot et al. 2016) with *A. gossypii* NM1 clone on *Vat-1^WMR 29^-*PmW4 and -PmW7 transgenic lines compared to the reference lines Védrantais (susceptible) and PI 161375 (resistant)

**(b) Assessment of the resistance to virus when inoculated by *Aphis gossypii***

Test carried on according to (Boissot et al. 2016) with CMV (isolate I17F) inoculated by *A. gossypii* (NM1 clone) on the *Vat-1^WMR 29^* PmW4 transgenic line compared to the reference lines Védrantais (S: susceptible) and Margot (R: resistant).

**(a)**

**(b)**

| Lines | Number of susceptible plants /number of plants tested | Phenotype |
| --- | --- | --- |
| Védrantais | 19/20 | S |
| PmW4 | 20/20 | S |
| Margot | 0/20 | R |

**Figure S2. Assessment of the resistance of two *Vat-1^PI 161375^*-Védrantais** **transgenic lines to *Podosphaera xanthii* race 1 (a) and race 3 (b)**

Mycelium colonization and sporulation intensity (0 to 7 scale) on the two transgenic lines *Vat-1^PI 161375^-*TR3 and *Vat-1^PI 161375^-*TR4 (Dogimont et al. 2014) compared to 5 reference lines at 8 days after inoculation. PMR45 was used as resistance control for race 1 and susceptible for race 3.

**
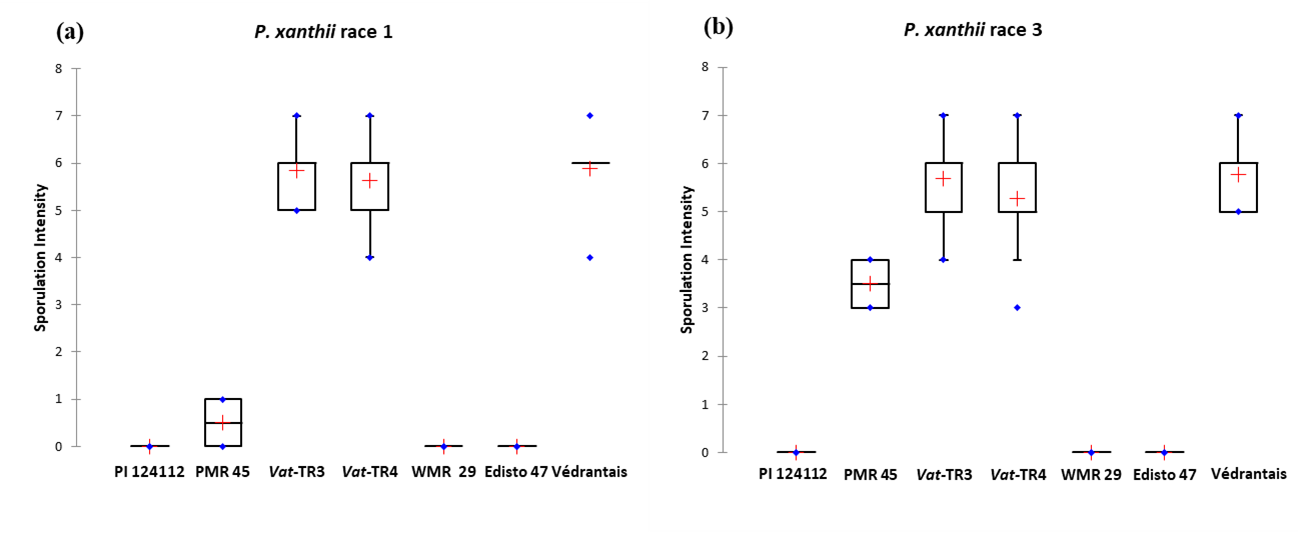
**

**Figure S3.** Time tree divergence of Vat-homologs with four (a) and five R65aa (b)

Analysis using the RelTime method with phylogenetic trees whose branch lengths were calculated using the Maximum Likelihood (ML) method and the Jones et al. w/freq. substitution model. The time trees were computed using 2 calibration constraints (outgroup min and max time divergence 6.9 and 10.2My according to divergence between *C. melo* and *C. sativus*)


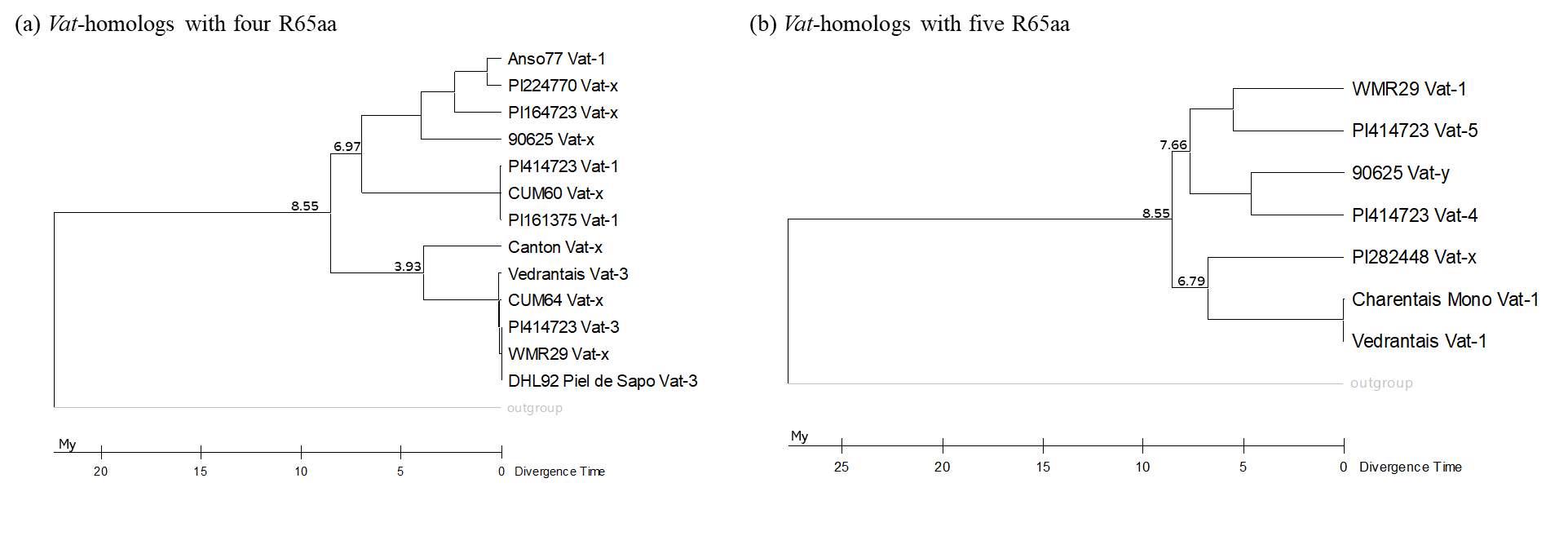


**SUPPLEMENTAL METHODS**

***Vat-1 ^WMR 29^* expression analysis in transgenic plants by qRT-PCR**

Experimental design, RNA extraction, reverse-transcription, candidate reference gene selection and primer design were conducted according to the MIQE guidelines ([Bustin et al., 2009](#_ENREF_10)), as previously reported (Dogimont et al., 2014). Specific markers Q299F and Q299R located in *Vat-1* ***^WMR 29^*** exon 3 and amplifying a 212 bp amplicon size were designed (Table S1b). The four transgenic melon lines (Pmw-TR3, Pmw-TR4, Pmw-TR7, Pmw-TR18) and the non-transgenic resistant control WMR 29 were assessed. Ten plants per genotype or transgenic line were cultivated in controlled conditions at 22-24°C and 60% relative humidity. 15 days after sowing a leaf disk was cut from each plant for total RNA extraction (RNeasy Plant Mini Kit Qiagen). RNA integrity was evaluated by agarose gel electrophoresis and purity was assessed by absorption ratio (Nanodrop ND-1000 spectrophotometer). After DNase treatment (RQ1, Promega) absence of DNA contamination in each RNA sample was controlled by a qPCR reaction (NoRT). 1.5 µg of DNase-treated RNA was primed with 2.5µM of oligo-d(T)21 and reverse-transcription was realized using 1 unit of SuperScript III (InVitrogen). The qPCR experiment comprised five biological samples per genotype, with five sample plants, and three technical replicates per sample. From each sample, 5µl was pooled to prepare a five-point five-fold serial dilution and the remaining was diluted 10-fold before being subjected to qPCR. The PCR amplification efficiency of four reference genes primers pairs were evaluated from the slope obtained with the serial five-point dilutions (efficiency comprised between 95 and 102% with a coefficient of correlation superior to 0.999). Primers sequences, amplicon lengths and amplification efficiency were given in (Table S1c). According to the GENORM software three stable reference genes, *TIP41*, *actin* and *TUA* (Anstead et al., 2010) were selected for building an accurate normalization factor to measure the *Vat-1 ^WMR 29^* gene expression by qPCR using the SYBR green chemistry. The quantitative real-time PCR experiments were carried out in a final volume of 20 µl, containing 10 µl of Mesagreen qPCR Master Mix Plus for SYBR Assay Low Rox (Eurogentec), 300 nM of each primer and 2 µl of cDNA. The data were analyzed as described in (Vandesompele et al., 2002) using the R package RqPCR analysis script ([Hilliou and Tran, 2013](#_ENREF_35)).
