# Supplementary material for: A highly diversified NLR cluster in melon contains homologs that confer powdery mildew and aphid resistance
Source: Hortic Res. 2023 Dec 13;11(1):uhad256. doi: 10.1093/hr/uhad256 (PMC10807702; doi:10.1093/hr/uhad256)
Supplement: Web_Material_uhad256 [file web_material_uhad256.zip › DataS3_4R65aa_CSativus_R2bis.fasta.rtf]

#CC-NBS-LLR1
#LRR2
#LRR3-N-term


>C_Sativus_9930_CDS_out_cluster
MDILVSVTAKIAEYTVVPVGRQLGYVIHIHANFQKLKTQVEKLKDTRESVQQNIYTARRN
AEDIKPAVEKWLKNVDDFVRESDKILANEGGHGRLCSTNLVQRHKLSRKASKMAYEVNEM
KNEGEGFNTVSYKNAIPSVDCSLQKVSDFLDLDSRKLTAEQIMDALSDDNVHRIGVYGMG
GVGKTMLVKEILRKIVESK-SFDEVVTSTISQTPDFKSIQGQLADKLGLKFERETIEGRA
PILRKRLKMERRILVVLDDIWEYIDLETIGIPSVEDHTGCKILFTSRNKHLISNQMCANQ
IFEIKVLGENESWNLFKAMAGKIVEASDLKPIAIQVVRECAGLPIAITTVAKALRNKPSD
IWNDALDQLKSVDVFMTNIGEMDKKVYLSLKLSYDCLGYEEVKLLFLLCSMFPEDFSIDM
EELHVYAMGMGFLHGVDTVVKGRRRIKKLVDDLISSSLLQQYSEYGYNYVKMHDMVRDVA
IFIASKNDHIRTLSYVKRLDEEWKEERLLGNHTVVSIHGLHYPLPKLMLPKVQLLRLDG-
-QWLNNTYVSVVQTFFEEMKELKGLVLEKVNISLLQRPFDLYFLANIRVLRLRGCE-LGS
IDMIGELKRLEILDLSGSNIIQIPTTMGQLTQLKVLNLSNCFNKLEIIPPNILSKLTKLE
ELRLGTFGSWEGEEWYEGRKNASLSELRFLPHLFDLDLTIQDEKIMPKHLFSAEELNLEN
FHITIGCKRERVKNYDGIIKMNYSRILEVKMESEMCLDDWIKFLLKRSEEVHLEGSICSK
VLNSELLDANGFLH--------LKNLWIFYNSDIQHFIHEKNKPLRKCLSKLEFLYLKNL
ENLESVIHGYNNGESPLNNLKNVIVWNCNKLKTLFLNCMLDDVLNLEEIEINYCKKMEVM
ITVKENEETTNHVEFTHLKSLCLWTLPQLHKFCSKVSNTINTCESFFSEEVSLPNLEKLK
IWCTKDLKKIWSNNVLIPNSFSKLKEIDIYSCNNLQKALFSPNMMSILTCLKVLRIEDCK
LLEGIFEVQEPISVVE--------------------------------------------
------------------------------------------------------------
------------------------------------------------------------
---------------------------------TSPIALQTLSELKLYKLPNLEYVWSKD
SCELQSLVNIKRLTMDECPRLRREYSVKILKQLEALSIDIKQLMEVIGKKKSTDYNRLES
KQLETSSSKVE-VLQLGDGSELFPKLKTLKLYGFVEDNSTHLPMEIVQNLYQFEK-FELE
GAFIEEILPSNILIPMKKQYNARRSKTSQRSWVLSKLPKLRHL-GSECSQKNNDSILQDL
TSLSISECGGLSS--LVSSSVSFTNLTFLKLNKCDGLTHLLNPSMATTLVQLKQLRIGEC
KRMSRIIEGGSSGEEDGNGEIIVFNNLQFLIITSCSNLTSFYRGRCIIQFPCLKHVSLEK
CPKMKSFSFGIVSTSHSKYEKCFFKE----------------------------------
------------------------------
>AM51_Vat-x
MDILISVTAKIAEYTVEPVGRQLGYVFFIRSNFQKLKTQVEKLKITRESVQHKIHSARRN
AEDIKPAVEEWLKKVDDFVRESDEILANEGGHGGLCSTYLVQRHKLSRKASKMVDEVLEM
KNEGESFDMVSYKSVIPSVDCSLPKVPDFLDFESRKSIMEQIMDALSDGNVHRIGVYGMG
GVGKTMLVKDILRKIVESKKPFDEVVTSTISQTPDFRSIQGQLADKLGLKFEQETIEGRA
TILRKRLKMERSILVVLDDVWEYIDLETIGIPSVEDHTGCKILFTTRIKHLISNQMCANK
IFEIKVLGKDESWNLFKAMAGDIVDASDLKPIAIRIVRECAGLPIAITTVAKALRNKPSD
IWNDALDQLKTVDVGMANIGEMEKKVYLSLKLSYDCLGYEEVKLLFLLCSMFPEDFSIDV
EGLHVYAMGMGFLHGVDTVVKGRRRIKKLVDDLISSSLLQQYSEYGCNYVKMHDMVRDVA
LLIASKNEHVRTLSYVKRSNEEWEEEKLLGNHTAVFIDGLHYPLPKLTLPKVQLLRLVAK
YCWEHNKRVSVVETFFEEMKELKGLVVENVNISLMQRPSDVYSLANIRVLRLERCQLLGS
IDWIGELKKLEILDFSESNITQIPTTMSQLTQLKVLNLSSCE-QLEVIPPNILSKLTKLE
ELDLETFDGWEGEEWYEGRKNASLSELKCLRHLYALNLTIQDEEIMPENLFLVGKLKLQK
FNICIGCESKLKYTFAYK--NRIKNFIGIKMESGRCLDDWIKNLLKRSDNVLLEGSVCSK
VLHSELVSLPNLEKLEIVNAKSLKMIW---------------------------------
------------------------------------------------------------
------------------------------------------------------------
-----------SNNVPILNSFSKLEEIKIYSCNNLQKVLFPPNMMDILTCLKVLEIKNCD
LLEGIFEAQEPISVVESNNLPILNSFSKLEEIRIWSCNNLQKVLFPSNMMGILPCLKVLD
IRGCELLEGIFEVQEPISVVESNSVPILNSFSKLEKIRIWSCNNLQKILFPSNMMGILTC
LKVLEIRDCELLEGIFEVQEPISVVESNNLPILNSFSKLEEIRIGSCNNLQKVLFPPNMM
GILTCLKVLEIRHCNLLEGIFEVQEPISIVEAS--PILLQNLSSLMLCNLPNLEYVWSKN
PYELLSLENIKSLTIDKCPRLRREYSVKILKQLEDVSIDIKQLMKVIEKEKSAHHNMLES
KQWETSSSSKDGVLRLGDGSKLFPNLKSLKLYGFVDYNSTHLPMEMLQILFQLVV-FELE
GAFLEEIFPSNILIP---------SYMVLRRLALSKLPKLKHLWSEECSQNNITSVLQHL
ISLRISECGRLSS--LLSSIVCFTNLKHLRVYKCDGLTHLLNPSVATTLVQLQSLTIEEC
KRMSSVIEGGST-EEDGNDEMVVFNNLQHLYIFNCSNLTSFYCGRCIIKFPCLRQVDIWN
CSEMKVFSLGIVSTPRLKYENFSLKNDYDDERCHPKYPKDMLVEDMNVITREYWEDNVDT
GIPNLFAEQSLEENRSENSSSSKNNVEKE-
>PI414723_Vat-1
MDILISVTAKIAEYTVEPVGRQLGYVFFIRSNFQKLKTQVEKLKITRESVQHKIHSARRN
AEDIKPAVEEWLKKVDDFVRESDEILANEGGHGGLCSTYLVQRHKLSRKASKMVDEVLEM
KNEGESFDMVSYKSVIPSVDCSLPKVPDFLDFESRKSIMEQIMDALSDGNVHRIGVYGMG
GVGKTMLVKDILRKIVESKKPFDEVVTSTISQTPDFRSIQGQLADKLGLKFEQETIEGRA
TILRKRLKMERSILVVLDDVWEYIDLETIGIPSVEDHTGCKILFTTRIKHLISNQMCANK
IFEIKVLGKDESWNLFKAMAGDIVDASDLKPIAIRIVRECAGLPIAITTVAKALRNKPSD
IWNDALDQLKTVDVGMANIGEMEKKVYLSLKLSYDCLGYEEVKLLFLLCSMFPEDFSIDV
EGLHVYAMGMGFLHGVDTVVKGRRRIKKLVDDLISSSLLQQYSEYGCNYVKMHDMVRDVA
LLIASKNEHVRTLSYVKRSNEEWEEEKLLGNHTAVFIDGLHYPLPKLTLPKVQLLRLVAK
YCWEHNKRVSVVETFFEEMKELKGLVVENVNISLMQRPSDVYSLANIRVLRLERCQLLGS
IDWIGELKKLEILDFSESNITQIPTTMSQLTQLKVLNLSSCE-QLEVIPPNILSKLTKLE
ELDLETFDGWEGEEWYEGRKNASLSELKCLRHLYALNLTIQDEEIMPENLFLVGKLKLQK
FNICIGCESKLKYTFAYK--NRIKNFIGIKMESGRCLDDWIKNLLKRSDNVLLEGSVCSK
VLHSELVSLPNLEKLEIVNAKSLKMIW---------------------------------
------------------------------------------------------------
------------------------------------------------------------
-----------SNNVPILNSFSKLEEIKIYSCNNLQKVLFPPNMMDILTCLKVLEIKNCD
LLEGIFEAQEPISVVESNNLPILNSFSKLEEIRIWSCNNLQKVLFPSNMMGILPCLKVLD
IRGCELLEGIFEVQEPISVVESNSVPILNSFSKLEKIRIWSCNNLQKILFPSNMMGILTC
LKVLEIRDCELLEGIFEVQEPISVVESNNLPILNSFSKLEEIRIGSCNNLQKVLFPPNMM
GILTCLKVLEIRHCNLLEGIFEVQEPISIVEAS--PILLQNLSSLMLCNLPNLEYVWSKN
PYELLSLENIKSLTIDKCPRLRREYSVKILKQLEDVSIDIKQLMKVIEKEKSAHHNMLES
KQWETSSSSKDGVLRLGDGSKLFPNLKSLKLYGFVDYNSTHLPMEMLQILFQLVV-FELE
GAFLEEIFPSNILIP---------SYMVLRRLALSKLPKLKHLWSEECSQNNITSVLQHL
ISLRISECGRLSS--LLSSIVCFTNLKHLRVYKCDGLTHLLNPSVATTLVQLQSLTIEEC
KRMSSVIEGGST-EEDGNDEMVVFNNLQHLYIFNCSNLTSFYCGRCIIKFPCLRQVDIWN
CSEMKVFSLGIVSTPRLKYENFSLKNDYDDERCHPKYPKDMLVEDMNVITREYWEDNVDT
GIPNLFAEQSLEENRSENSSSSKNNVEKE*
>PI482398_Vat-x
MDILISVTAKIAEYTVEPVGRQLGYVFFIRSNFQKLKTQVEKLKITRESVQHKIHSARRN
AEDIKPAVEEWLKKVDDFVRESDEILANEGGHGGLCSTYLVQRHKLSRKASKMVDEVLEM
KNEGESFDMVSYKSVIPSVDCSLPKVPDFLDFESRKSIMEQIMDALSDGNVHRIGVYGMG
GVGKTMLVKDILRKIVESKKPFDEVVTSTISQTPDFRSIQGQLADKLGLKFEQETIEGRA
TILRKRLKMERSILVVLDDVWEYIDLETIGIPSVEDHTGCKILFTTRIKHLISNQMCANK
IFEIKVLGKDESWNLFKAMAGDIVDASDLKPIAIRIVRECAGLPIAITTVAKALRNKPSD
IWNDALDQLKTVDVGMANIGEMEKKVYLSLKLSYDCLGYEEVKLLFLLCSMFPEDFSIDV
EGLHVYAMGMGFLHGVDTVVKGRRRIKKLVDDLISSSLLQQYSEYGCNYVKMHDMVRDVA
LLIASKNEHVRTLSYVKRSNEEWEEEKLLGNHTAVFIDGLHYPLPKLTLPKVQLLRLVAK
YCWEHNKRVSVVETFFEEMKELKGLVVENVNISLMQRPSDVYSLANIRVLRLERCQLLGS
IDWIGELKKLEILDFSESNITQIPTTMSQLTQLKVLNLSSCE-QLEVIPPNILSKLTKLE
ELDLETFDGWEGEEWYEGRKNASLSELKCLRHLYALNLTIQDEEIMPENLFLVGKLKLQK
FNICIGCESKLKYTFAYK--NRIKNFIGIKMESGRCLDDWIKNLLKRSDNVLLEGSVCSK
VLHSELVSLPNLEKLEIVNAKSLKMIW---------------------------------
------------------------------------------------------------
------------------------------------------------------------
-----------SNNVPILNSFSKLEEIKIYSCNNLQKVLFPPNMMDILTCLKVLEIKNCD
LLEGIFEAQEPISVVESNNLPILNSFSKLEEIRIWSCNNLQKVLFPSNMMGILPCLKVLD
IRGCELLEGIFEVQEPISVVESNSVPILNSFSKLEKIRIWSCNNLQKILFPSNMMGILTC
LKVLEIRDCELLEGIFEVQEPISVVESNNLPILNSFSKLEEIRIGSCNNLQKVLFPPNMM
GILTCLKVLEIRHCNLLEGIFEVQEPISIVEAS--PILLQNLSSLMLCNLPNLEYVWSKN
PYELLSLENIKSLTIDKCPRLRREYSVKILKQLEDVSIDIKQLMKVIEKEKSAHHNMLES
KQWETSSSSKDGVLRLGDGSKLFPNLKSLKLYGFVDYNSTHLPMEMLQILFQLVV-FELE
GAFLEEIFPSNILIP---------SYMVLRRLALSKLPKLKHLWSEECSQNNITSVLQHL
ISLRISECGRLSS--LLSSIVCFTNLKHLRVYKCDGLTHLLNPSVATTLVQLQSLTIEEC
KRMSSVIEGGST-EEDGNDEMVVFNNLQHLYIFNCSNLTSFYCGRCIIKFPCLRQVDIWN
CSEMKVFSLGIVSTPRLKYENFSLKNDYDDERCHPKYPKDMLVEDMNVITREYWEDNVDT
GIPNLFAEQSLEENRSENSSSSKNNVEKE-
>PI164323_Vat-x
MDILISVTAKIAEYTVEPVGRQLGYVFFIRSNFQKLKTQVEKLKITRESVQHKIHSARRN
AEDIKPAVEEWLKKVDDFVRESDEILANEGGHGGLCSTYLVQRHKLSRKASKMVDEVLEM
KNEGESFDMVSYKSVIPSVDCSLPKVPDFLDFESRKSIMEQIMDALSDGNVHRIGVYGMG
GVGKTMLVKDILRKIVESKKPFDEVVTSTISQTPDFRSIQGQLADKLGLKFEQETIEGRA
TILRKRLKMERSILVVLDDVWEYIDLETIGIPSVEDHTGCKILFTTRIKHLISNQMCANK
IFEIKVLGKDESWNLFKAMAGDIVDASDLKPIAIRIVRECAGLPIAITTVAKALRNKPSD
IWNDALDQLKTVDVGMANIGEMEKKVYLSLKLSYDCLGYEEVKLLFLLCSMFPEDFSIDV
EGLHVYAMGMGFLHGVDTVVKGRRRIKKLVDDLISSSLLQQYSEYGCNYVKMHDMVRDVA
LLIASKNEHVRTLSYVKRSNEEWEEEKLLGNHTAVFIDGLHYPLPKLTLPKVQLLRLVAK
YCWEHNKRVSVVETFFEEMKELKGLVVENVNISLMQRPSDVYSLANIRVLRLERCQLLGS
IDWIGELKKLEILDFSESNITQIPTTMSQLTQLKVLNLSSCE-QLEVIPPNILSKLTKLE
ELDLETFDGWEGEEWYEGRKNASLSELKCLRHLYALNLTIQDEEIMPENLFLVGKLKLQK
FNICIGCESKLKYTFAYK--NRIKNFIGIKMESGRCLDDWIKNLLKRSDNVLLEGSVCSK
VLHSELVSLPNLEKLEIVNAKSLKMIW---------------------------------
------------------------------------------------------------
------------------------------------------------------------
-----------SNNVPILNSFSKLEEIKIYSCNNLQKVLFPPNMMDILTCLKVLEIKNCD
LLEGIFEAQEPISVVESNNLPILNSFSKLEEIRIWSCNNLQKVLFPSNMMGILPCLKVLD
IRGCELLEGIFEVQEPISVVESNSVPILNSFSKLEKIRIWSCNNLQKILFPSNMMGILTC
LKVLEIRDCELLEGIFEVQEPISVVESNNLPILNSFSKLEEIRIGSCNNLQKVLFPPNMM
GILTCLKVLEIRHCNLLEGIFEVQEPISIVEAS--PILLQNLSSLMLCNLPNLEYVWSKN
PYELLSLENIKSLTIDKCPRLRREYSVKILKQLEDVSIDIKQLMKVIEKEKSAHHNMLES
KQWETSSSSKDGVLRLGDGSKLFPNLKSLKLYGFVDYNSTHLPMEMLQILFQLVV-FELE
GAFLEEIFPSNILIP---------SYMVLRRLALSKLPKLKHLWSEECSQNNITSVLQHL
ISLRISECGRLSS--LLSSIVCFTNLKHLRVYKCDGLTHLLNPSVATTLVQLESLTIEEC
KRMSSVIEGGST-EEDGNDEMVVFNNLQHLYIFNCSNLTSFYCGRCIIKFPCLRQVDIWN
CSEMKVFSLGIVSTPRLKYENFSLKNDYDDERCHPKYPKDMLVEDMNVITREYWEDNVDT
GIPNLFAEQSLEENRSENSSSSKNNVEKE*
>CUM60_Vat-x
MDILISVTAKIAEYTVEPVGRQLGYVFFIRSNFQKLKTQVEKLKITRESVQHKIHSARRN
AEDIKPAVEEWLKKVDDFVRESDEILANEGGHGGLCSTYLVQRHKLSRKASKMVDEVLEM
KNEGESFDMVSYKSVIPSVDCSLPKVPDFLDFESRKSIMEQIMDALSDGNVHRIGVYGMG
GVGKTMLVKDILRKIVESKKPFDEVVTSTISQTPDFRSIQGQLADKLGLKFEQETIEGRA
TILRKRLKMERSILVVLDDVWEYIDLETIGIPSVEDHTGCKILFTTRIKHLISNQMCANK
IFEIKVLGKDESWNLFKAMAGDIVDASDLKPIAIRIVRECAGLPIAITTVAKALRNKPSD
IWNDALDQLKTVDVGMANIGEMEKKVYLSLKLSYDCLGYEEVKLLFLLCSMFPEDFSIDV
EGLHVYAMGMGFLHGVDTVVKGRRRIKKLVDDLISSSLLQQYSEYGCNYVKMHDMVRDVA
LLIASKNEHVRTLSYVKRSNEEWEEEKLLGNHTAVFIDGLHYPLPKLTLPKVQLLRLVAK
YCWEHNKRVSVVETFFEEMKELKGLVVENVNISLMQRPSDVYSLANIRVLRLERCQLLGS
IDWIDELKKLEILDFSESNITQIPTTMSQLTQLKVLNLSSCE-QLEVIPPNILSKLTKLE
ELDLETFDGWEGEEWYEGRKNASLSELKCLRHLYALNLTIQDEEIMPENLFLVGKLKLQK
FNICIGCESKLKYTFAYK--NRIKNFIGIKMESGRCLDDWIKNLLKRSDNVLLEGSVCSK
VLHSELVSLPNLEKLEIVNAKSLKMIW---------------------------------
------------------------------------------------------------
------------------------------------------------------------
-----------SNNVPILNSFSKLEEIKIYSCNNLQKVLFPPNMMDILTCLKVLEIKNCD
LLEGIFEAQEPISVVESNNLPILNSFSKLEEIRIWSCNNLQKVLFPSNMMGILPCLKVLD
IRGCELLEGIFEVQEPISVVESNSVPILNSFSKLEKIRIWSCNNLQKILFPSNMMGILTC
LKVLEIRDCELLEGIFEVQEPISVVESNNLPILNSFSKLEEIRIGSCNNLQKVLFPPNMM
GILTCLKVLEIRHCNLLEGIFEVQEPISIVEAS--PILLQNLSSLMLCNLPNLEYVWSKN
PYELLSLENIKSLTIDKCPRLRREYSVKILKQLEDVSIDIKQLMKVIEKEKSAHHNMLES
KQWETSSSSKDGVLRLGDGSKLFPNLKSLKLYGFVDYNSTHLPMEMLQILFQLVV-FELE
GAFLEEIFPSNILIP---------SYMVLRRLALSKLPKLKHLWSEECSQNNITSVLQHL
ISLRISECGRLSS--LLSSIVCFTNLKHLRVYKCDGLTHLLNPSVATTLVQLESLTIEEC
KRMSSVIEGGST-EEDGNDEMVVFNNLQHLYIFNCSNLTSFYCGRCIIKFPCLRQVDIWN
CSEMKVFSLGIVSTPRLKYENFSLKNDYDDERCHPKYPKDMLVEDMNVITREYWEDNVDT
GIPNLFAEQSLEENRSENSSSSKNNVEKE-
>PI161375_Vat-1
MDILISVTAKIAEYTVEPVGRQLGYVFFIRSNFQKLKTQVEKLKITRESVQHKIHSARRN
AEDIKPAVEEWLKKVDDFVRESDEILANEGGHGGLCSTYLVQRHKLSRKASKMVDEVLEM
KNEGESFDMVSYKSVIPSVDCSLPKVPDFLDFESRKSIMEQIMDALSDGNVHRIGVYGMG
GVGKTMLVKDILRKIVESKKPFDEVVTSTISQTPDFRSIQGQLADKLGLKFEQETIEGRA
TILRKRLKMERSILVVLDDVWEYIDLETIGIPSVEDHTGCKILFTTRIKHLISNQMCANK
IFEIKVLGKDESWNLFKAMAGDIVDASDLKPIAIRIVRECAGLPIAITTVAKALRNKPSD
IWNDALDQLKTVDVGMANIGEMEKKVYLSLKLSYDCLGYEEVKLLFLLCSMFPEDFSIDV
EGLHVYAMGMGFLHGVDTVVKGRRRIKKLVDDLISSSLLQQYSEYGCNYVKMHDMVRDVA
LLIASKNEHVRTLSYVKRSNEEWEEEKLLGNHTAVFIDGLHYPLPKLTLPKVQLLRLVAK
YCWEHNKRVSVVETFFEEMKELKGLVVENVNISLMQRPSDVYSLANIRVLRLERCQLLGS
IDWIGELKKLEILDFSESNITQIPTTMSQLTQLKVLNLSSCE-QLEVIPPNILSKLTKLE
ELDLETFDGWEGEEWYEGRKNASLSELKCLRHLYALNLTIQDEEIMPENLFLVGKLKLQK
FNICIGCESKLKYTFAYK--NRIKNFIGIKMESGRCLDDWIKNLLKRSDNVLLEGSVCSK
VLHSELVSLPNLEKLEIVNAKSLKMIW---------------------------------
------------------------------------------------------------
------------------------------------------------------------
-----------SNNVPILNSFSKLEEIKIYSCNNLQKVLFPPNMMDILTCLKVLEIKNCD
LLEGIFEAQEPISVVESNNLPILNSFSKLEEIRIWSCNNLQKVLFPSNMMGILPCLKVLD
IRGCELLEGIFEVQEPISVVESNSVPILNSFSKLEKIRIWSCNNLQKILFPSNMMGILTC
LKVLEIRDCELLEGIFEVQEPISVVESNNLPILNSFSKLEEIRIGSCNNLQKVLFPPNMM
GILTCLKVLEIRHCNLLEGIFEVQEPISIVEAS--PILLQNLSSLMLCNLPNLEYVWSKN
PYELLSLENIKSLTIDKCPRLRREYSVKILKQLEDVSIDIKQLMKVIEKEKSAHHNMLES
KQWETSSSSKDGVLRLGDGSKLFPNLKSLKLYGFVDYNSTHLPMEMLQILFQLVV-FELE
GAFLEEIFPSNILIP---------SYMVLRRLALSKLPKLKHLWSEECSQNNITSVLQHL
ISLRISECGRLSS--LLSSIVCFTNLKHLRVYKCDGLTHLLNPSVATTLVQLESLTIEEC
KRMSSVIEGGST-EEDGNDEMVVFNNLQHLYIFNCSNLTSFYCGRCIIKFPCLRQVDIWN
CSEMKVFSLGIVSTPRLKYENFSLKNDYDDERCHPKYPKDMLVEDMNVITREYWEDNVDT
GIPNLFAEQSLEENRSENSSSSKNNVEKE-
>Lanzhou1_Vat-x
MDILISVTAKIAEYTVEPVGRQLGYVFFIRSNFQKLKTQVEKLKITRESVQHKIHSARRN
AEDIKPAVEEWLKKVDDFVRESDEILANEGGHGGLCSTYLVQRHKLSRKASKMVDEVLEM
KNEGESFDMVSYKSVIPSVDCSLPKVPDFLDFESRKSIMEQIMDALSDGNVHRIGVYGMG
GVGKTMLVKDILRKIVESKKPFDEVVTSTISQTPDFRSIQGQLADKLGLKFEQETIEGRA
TILRKRLKMERSILVVLDDVWEYIDLETIGIPSVEDHTGCKILFTTRIKHLISNQMCANK
IFEIKVLGKDESWNLFKAMAGDIVDASDLKPIAIRIVRECAGLPIAITTVAKALRNKPSD
IWNDALDQLKTVDVGMANIGEMEKKVYLSLKLSYDCLGYEEVKLLFLLCSMFPEDFSIDV
EGLHVYAMGMGFLHGVDTVVKGRRRIKKLVDDLISSSLLQQYSEYGCNYVKMHDMVRDVA
LLIASKNEHVRTLSYVKRSNEEWEEEKLLGNHTAVFIDGLHYPLPKLTLPKVQLLRLVAK
YCWEHNKRVSVVETFFEEMKELKGLVVENVNISLMQRPSDVYSLANIRVLRLERCQLLGS
IDWIGELKKLEILDFSESNITQIPTTMSQLTQLKVLNLSSCE-QLEVIPPNILSKLTKLE
ELDLETFDGWEGEEWYEGRKNASLSELKCLRHLYALNLTIQDEEIMPENLFLVGKLKLQK
FNICIGCESKLKYTFAYK--NRIKNFIGIKMESGRCLDDWIKNLLKRSDNVLLEGSVCSK
VLHSELVSLPNLEKLEIVNAKSLKMIW---------------------------------
------------------------------------------------------------
------------------------------------------------------------
-----------SNNVPILNSFSKLEEIKIYSCNNLQKVLFPPNMMDILTCLKVLEIKNCD
LLEGIFEAQEPISVVESNNLPILNSFSKLEEIRIWSCNNLQKVLFPSNMMGILPCLKVLD
IRGCELLEGIFEVQEPISVVESNSVPILNSFSKLEKIRIWSCNNLQKILFPSNMMGILTC
LKVLEIRDCELLEGIFEVQEPISVVESNNLPILNSFSKLEEIRIGSCNNLQKVLFPPNMM
GILTCLKVLEIRHCNLLEGIFEVQEPISIVEAS--PILLQNLSSLMLCNLPNLEYVWSKN
PYELLSLENIKSLTIDKCPRLRREYSVKILKQLEDVSIDIKQLMKVIEKEKSAHHNMLES
KQWETSSSSKDGVLRLGDGSKLFPNLKSLKLYGFVDYNSTHLPMEMLQILFQLVV-FELE
GAFLEEIFPSNILIP---------SYMVLRRLALSKLPKLKHLWSEECSQNNITSVLQHL
ISLRISECGRLSS--LLSSIVCFTNLKHLRVYKCDGLTHLLNPSVATTLVQLESLTIEEC
KRMSSVIEGGST-EEDGNDEMVVFNNLQHLYIFNCSNLTSFYCGRCIIKFPCLRQVDIWN
CSEMKVFSLGIVSTPRLKYENFSLKNDYDDERCHPKYPKDMLVEDMNVITREYWEDNVDT
GIPNLFAEQSLEENRSENSSSSKNNVEKE-
>Rachibbar_Vat-x
MDILISVTAKIAEYTVEPVGRQLGYVFFIRSNFQKLKTQVEKLKITRESVQHKIHSARRN
AEDIKPAVEEWLKKVDDFVRESDEILANEGGHGGLCSTYLVQRHKLSRKASKMVDEVLEM
KNEGESFDMVSYKSVIPSVDCSLPKVPDFLDFESRKSIMEQIMDALSDGNVHRIGVYGMG
GVGKTMLVKDILRKIVESKKPFDEVVTSTISQTPDFRSIQGQLADKLGLKFEQETIEGRA
TILRKRLKMERSILVVLDDVWEYIDLETIGIPSVEDHTGCKILFTTRIKHLISNQMCANK
IFEIKVLGKDESWNLFKAMAGDIVDASDLKPIAIRIVRECAGLPIAITTVAKALRNKPSD
IWNDALDQLKTVDVGMANIGEMEKKVYLSLKLSYDCLGYEEVKLLFLLCSMFPEDFSIDV
EGLHVYAMGMGFLHGVDTVVKGRRRIKKLVDDLISSSLLQQYSEYGCNYVKMHDMVRDVA
LLIASKNEHVRTLSYVKRSNEEWEEEKLLGNHTAVFIDGLHYPLPKLTLPKVQLLRLVAK
YCWEHNKRVSVVETFFEEMKELKGLVVENVNISLMQRPSDVYSLANIRVLRLERCQLLGS
IDWIGELKKLEILDFSESNITQIPTTMSQLTQLKVLNLSSCE-QLEVIPPNILSKLTKLE
ELDLETFDGWEGEEWYEGRKNASLSELKCLRHLYALNLTIQDEEIMPENLFLVGKLKLQK
FNICIGCESKLKYTFAYK--NRIKNFIGIKMESGRCLDDWIKNLLKRSDNVLLEGSVCSK
VLHSELVSLPNLEKLEIVNAKSLKMIW---------------------------------
------------------------------------------------------------
------------------------------------------------------------
-----------SNNVPILNSFSKLEEIKIYSCNNLQKVLFPPNMMDILTCLKVLEIKNCD
LLEGIFEAQEPISVVESNNLPILNSFSKLEEIRIWSCNNLQKVLFPSNMMGILPCLKVLD
IRGCELLEGIFEVQEPISVVESNSVPILNSFSKLEKIRIWSCNNLQKILFPSNMMGILTC
LKVLEIRDCELLEGIFEVQEPISVVESNNLPILNSFSKLEEIRIGSCNNLQKVLFPPNMM
GILTCLKVLEIRHCNLLEGIFEVQEPISIVEAS--PILLQNLSSLMLCNLPNLEYVWSKN
PYELLSLENIKSLTIDKCPRLRREYSVKILKQLEDVSIDIKQLMKVIEKEKSAHHNMLES
KQWETSSSSKDGVLRLGDGSKLFPNLKSLKLYGFVDYNSTHLPMEMLQILFQLVV-FELE
GAFLEEIFPSNILIP---------SYMVLRRLALSKLPKLKHLWSEECSQNNITSVLQHL
ISLRISECGRLSS--LLSSIVCFTNLKHLRVYKCDGLTHLLNPSVATTLVQLESLTIEEC
KRMSSVIEGGST-EEDGNDEMVVFNNLQHLYIFNCSNLTSFYCGRCIIKFPCLRQVDIWN
CSEMKVFSLGIVSTPRLKYENFSLKNDYDDERCHPKYPKDMLVEDMNVITREYWEDNVDT
GIPNLFAEQSLEENRSENSSSSKNNVEKE*
>SanIldefonso_Vat-x
MDILISVTAKIAEYTVEPVGRQLGYVFFIRSNFQKLKTQVEKLKITRESVQHKIHSARRN
AEDIKPAVEEWLKKVDDFVRESDEILANEGGHGGLCSTYLVQRHKLSRKASKMVDEVLEM
KNEGESFDMVSYKSVIPSVDCSLPKVPDFLDFESRKSIMEQIMDALSDGNVHRIGVYGMG
GVGKTMLVKDILRKIVESKKPFDEVVTSTISQTPDFRSIQGQLADKLGLKFEQETIEGRA
TILRKRLKMERSILVVLDDVWEYIDLETIGIPSVEDHTGCKILFTTRIKHLISNQMCANK
IFEIKVLGKDESWNLFKAMAGDIVDASDLKPIAIRIVRECAGLPIAITTVAKALRNKPSD
IWNDALDQLKTVDVGMANIGEMEKKVYLSLKLSYDCLGYEEVKLLFLLCSMFPEDFSIDV
EGLHVYAMGMGFLHGVDTVVKGRRRIKKLVDDLISSSLLQQYSEYGCNYVKMHDMVRDVA
LLIASKNEHVRTLSYVKRSNEEWEEEKLLGNHTAVFIDGLHYPLPKLTLPKVQLLRLVAK
YCWEHNKRVSVVETFFEEMKELKGLVVENVNISLMQRPSDVYSLANIRVLRLERCQLLGS
IDWIGELKKLEILDFSESNITQIPTTMSQLTQLKVLNLSSCE-QLEVIPPNILSKLTKLE
ELDLETFDGWEGEEWYEGRKNASLSELKCLRHLYALNLTIQDEEIMPENLFLVGKLKLQK
FNICIGCESKLKYTFAYK--NRIKNFIGIKMESGRCLDDWIKNLLKRSDNVLLEGSVCSK
VLHSELVSLPNLEKLEIVNAKSLKMIW---------------------------------
------------------------------------------------------------
------------------------------------------------------------
-----------SNNVPILNSFSKLEEIKIYSCNNLQKVLFPPNMMDILTCLKVLEIKNCD
LLEGIFEAQEPISVVESNNLPILNSFSKLEEIRIWSCNNLQKVLFPSNMMGILPCLKVLD
IRGCELLEGIFEVQEPISVVESNSVPILNSFSKLEKIRIWSCNNLQKILFPSNMMGILTC
LKVLEIRDCELLEGIFEVQEPISVVESNNLPILNSFSKLEEIRIGSCNNLQKVLFPPNMM
GILTCLKVLEIRHCNLLEGIFEVQEPISIVEAS--PILLQNLSSLMLCNLPNLEYVWSKN
PYELLSLENIKSLTIDKCPRLRREYSVKILKQLEDVSIDIKQLMKVIEKEKSAHHNMLES
KQWETSSSSKDGVLRLGDGSKLFPNLKSLKLYGFVDYNSTHLPMEMLQILFQLVV-FELE
GAFLEEIFPSNILIP---------SYMVLRRLALSKLPKLKHLWSEECSQNNITSVLQHL
ISLRISECGRLSS--LLSSIVCFTNLKHLRVYKCDGLTHLLNPSVATTLVQLESLTIEEC
KRMSSVIEGGST-EEDGNDEMVVFNNLQHLYIFNCSNLTSFYCGRCIIKFPCLRQVDIWN
CSEMKVFSLGIVSTPRLKYENFSLKNDYDDERCHPKYPKDMLVEDMNVITREYWEDNVDT
GIPNLFAEQSLEENRSENSSSSKNNVEKE-
>90625_Vat-x
MDILISVIAKIAEYTVEPVGRQLGYVFFIRSNFQKLKTQVEKLKITRESVQHKIHSARRN
AEDIKPAVEEWLKKVDDFVRESDEILANEGGHGGLCSTYLVQRHKLSRKASKMVDEVLEM
KNEGESFDMVSYKSVIPSVDCSLPKVPDFLDFESRKSIMEQIMDALSDGNVHRIGVYGMG
GVGKTMLVKDILRKIVESKKPFDEVVTSTISQTPDFRSIQGQLADTLGLKLEQETIEGRA
PILRKRLKMERSILVVLDDVWENIDLETIGIPSVEDHTGCKILFTARNKHLISNQMCANK
IFEIKVLGEDESWNLFKTMAGETVEASDLKPIAIQIARECAGLPIAITTVAKALRNKPSE
IWNDALNQIKSVDVGMANIGEMEKKVYLSLKLSYDCLGYEEVKLLFLLCSMFPEDFPIDV
EELHVYAMGMGFLHGVDTVVKGRCRIKKLVDDLISSSLLQQYSEYGCNYVKMHDMVRDVA
LLIASKNEHVRTLSYVKRSNEEWEEEKLLGNHTAVFIDGLHYPLPKLTLPKVQLLRLVAK
YCWEHNKCVSVVETFFEEMKELKGLVVENVNISLMQRPSDVYSLANIRVLRLERCQLLGS
IDWIGELKKLEILDFSESNITQIPTTMSQLTQLKVLNLSSCE-QLEVIPPNILSKLTKLE
ELDLETFDGWEGEEWYEGRKNASLSELKCLRHLYALSLTIQDEEIMPENLFLVGKLKLQK
FNICIGCESKLKYTFAYKNKNRIKNFIGIKMESGRCLDDWIKNLLKRSDNVLLEGSVCSK
VLHSELVSLPNLEKLKIVNAESLKMIW---------------------------------
------------------------------------------------------------
------------------------------------------------------------
-----------SNNVPILNSFSKLEEIRIRSCNNLQKVLFPPNMMGILTCLKDLEIEDCE
LLEGIFEVQEPISVLESNNLPILNSFSKLEKIIIASCNNLQKVLFPSNMMDILTCLKVLE
IRRCELLEGIFEVQEPISVVESNNVPILNSFSKLEKIRIWSCNNLQKVLFPSNMMDILTC
LKVLEIRRCELLEAIFEVQEPISVVESNNVPILNSFSKLEEIRIWSCNNLQKVLFHPNMM
GILTCLKVLVIRDCKLLEGIFEVQEPISVVEAS--PIVLQNLSRLKLYNLPNLEYLWSKN
PCELLSLENIKNLTIEECPRLRREYSVKILKPLEDVSIDIKQLMKVIEKEKSADHNMLES
KQWETSSSSKDGVLRLGDGSKLSPYLKSLKLYGFVDYNSTHLPMEMLQILFQLED-FELE
GAFIEEIFPSNILIS---------SSMDLQSLTLSKLPKLKHLWSEECSQNNITSVLQHL
FFLRISDCGRLSSLTLVSSLVCFTNLKDLHVNKCHRLTHLLNPSVATTLVQLEGLTVEEC
KRMSSVIEGGST-EEDGNDEIIVFNKLRNLTITSCSNLTSFYCGRCIIKFPRLRQVHIRD
CPEMKVFSIGIVSTPRLKYENFSLKNDYGDGQCHPKYPKDMLVEDMNVITREYWEDNVDT
RIPNLFAEQSLEENQYESFSSSNNNVEKE-
>PI164723_Vat-x
MDILISVTAKIAEYTVEPVGRQLGYVFFIRSNFQKLKTQVEKLKITRESVQHKIHSARRN
AEDIKPAVEEWLKKVDDFVRESDEILANEGGHGGLCSTYLVQRHKLSRKASKMVDEVLEM
KNEGESFDMVSYKSVIPSVDCSLPKVPDFLDFESRKSIMEQIMDALSDGNVHRIGVYGMG
GVGKTMLVKDILRKIVESKKPFDEVVTSTISQTPDFRSIQGQLADTLGLKFEQETIEGRA
PILRKRLKMERSILVVLDDVWENIDLETIGIPSVEDHTGCKILFTTRNKHLISNQMCANK
IFEIKVLGEDESWNLFKTMAGETVEASDLKPIAIQIVRECAGLPIAITTVAKALRNKPSD
IWNDALNQLKSVDVGMANIGEMERKVYLPLKLSYDCLGYEEVKLLFLLCSMFPEDFPIDV
EELHVYAMGMGFLHGVDTVEKGRCRIKKLVDDLISSSLLQQYSEYGCNYVKMHDMVRDVA
LLIASKNEHVRTLSYVKRSNEEWEEEKLLGNHTAVFIDGLHYPLPKLTLPKVQLLRLVAL
YCWEHNKCVSVVETFFEEMKELKGLVVENVNISLMQRPSDLYSLANIRVLRLERCQLLGS
IDWIGELKKLEILDFSESNITQIPTTMSQLTQLKVLNLSSCE-QLEVIPPNILSKLTKLE
ELNLETFDGWEGEEWYEGRKNASLSELKCLRHLYALNLTIQDEEIMPENLFLVGKLKLQK
FNIRIGCQSKLKYTLPYQNKNRIKNFIGIKMESGRCLDDWIKNLLKRSDNVLLEGSVCSK
VLHSELVSLPNLEKLEIANAESLKMIW---------------------------------
------------------------------------------------------------
------------------------------------------------------------
-----------SNNVPILNSFSKLEEIRIWSCNNLQKVLFHPNMMGILTCLKVLVIRDCK
LLEGIFEVQEPISVVESNNVPILNSFSKLEEIRIWSCNNLQKVLFPPNMMGILTCLKVLE
IIGCNLLEGIFEVQEPISVVESNNVPILNSFSKLEEIRIWSCNNLQKVLFPSNMMDVLTC
LKVLDIRDCELLEGIFEVQEPISVVESNNVPILNSFSKLEEIRIWSCNNLQKVLFPSNMM
DILTCLKVLDIRDCELLEGIFEVQEPISVVEAS--PIVLQNLSRLKLYNLPNLEYLWSKN
PCELLSLENIKNLTIEECPRLRREYSVKILKPLEDVSIDIKQLMKVIEKEKSADHNMLES
KQWETSSSSKDGVLRLGDGSKLFPYLKSLKLYGFVDYNSTHLPMEMLQILFQLED-FELE
GAFIEEIFPSNILIS---------SSMDLQSLTLSKLPKLKHLWSEECSQNNITSVLQHL
FFLRISDCGRLSSLTLVSSLVCFTNLKDLHVNKRHRLTHLLNPSVATTLVQLEGLTVEEC
KRMSSVIEGGST-EEDGNDEIIVFNKLRNLTITSCSNLTSFYCGRCIIKFPRLRQVHIRD
CPEMKVFSIGIVSTPRLKYENFSLKNDYGDGQCHPKYPKDMLVEDMNVITREYWEDNVDT
RIPNLFAEQSLEENQYENFSSSNNNVEKE-
>Anso77_Vat-1
MDILISVTAKIAEYTVEPVGRQLGYVFFIRSNFQKLKTQVEKLKITRESVQHKIHSARRN
AEDIKPAVEEWLKKVDDFVRESDEILANEGGHGGLCSTYLVQRHKLSRKASKMVDEVLEM
KNEGESFDMVSYKSVIPSVDCSLPKVPDFLDFESRKSIMEQIMDALSDGNVHRIGVYGMG
GVGKTMLVKDILRKIVESKKPFDEVVTSTISQTPDFRSIQGQLADTLGLKFEQETIEGRA
PILRKRLKMERSILVVLDDVWENIDLETIGIPSVEDHTGCKILFTTRNKHLISNQMCANK
IFEIKVLGEDESWNLFKTMAGETVEASDLKPIAIQIVRECAGLPIAITTVAKALRNKPSD
IWNDALNQLKSVDVGMANIGEMERKVYLPLKLSYDCLGYEEVKLLFLLCSMFPEDFPIDV
EELHVYAMGMGFLHGVDTVEKGRCRIKKLVDDLISSSLLQQYSEYGCNYVKMHDMVRDVA
LLIASKNEHVRTLSYVKRSNEEWEEEKLLGNHTAVFIDGLHYPLPKLTLPKVQLLRLVAL
YCWEHNKCVSVVETFFEEMKELKGLVVENVNISLMQRPSDLYSLANIRVLRLERCQLLGS
IDWIGELKKLEILDFSESNITQIPTTMSQLTQLKVLNLSSCE-QLEVIPPNILSKLTKLE
ELNLETFDGWEGEEWYEGRKNASLSELKCLRHLYALNLTIQDEEIMPENLFLVGKLKLQK
FNIRIGCQSKLKYTLPYQNKNRIKNFIGIKMESGRCLDDWIKNLLKRSDNVLLEGSVCSK
VLHSELVSLPNLEKLEIANAESLKMIW---------------------------------
------------------------------------------------------------
------------------------------------------------------------
-----------SNNVPILNSFSKLEEIRIWSCNNLQKVLFHPNMMGILTCLKVLVIRDCK
LLEGIFEVQEPISVVESNNVPILNSFSKLEEIRIWSCNNLQKVLFPPNMMGILTCLKVLE
IIGCNLLEGIFEVQEPISVVESNNVPILNSFSKLEEIRIWSCNNLQKVLFHPNMMGILTC
LKVLVIRNCKLLEGIFEVQEPISVVESNNIPILNSFSKLEEIRIWSCNNLQKVLFPPNMM
GILTCLKVLEIIGCNLLEGIFEVQEPISVVEAS--PIVLQNLSRLKLYNLPNLEYLWSKN
PCELLSLENIKNLTIEECPRLRREYSVKILKPLEDVSIDIKQLMKVIEKEKSADHNMLES
KQWETSSSSKDGVLRLGDGSKLFPYLKSLKLYGFVDYNSTHLPMEMLQILFQLED-FELE
GAFIEEIFPSNILIS---------SSMDLQSLTLSKLPKLKHLWSEECSQNNITSVLQHL
FFLRISDCGRLSSLTLVSSLVCFTNLKDLHVNKCHRLTHLLNPSVATTLVQLEGLTVEEC
KRMSSVIEGGST-EEDGNDEIIVFNKLRNLTITSCSNLTSFYCGRCIIKFPRLRQVHIRD
CPEMKVFSIGIVSTPRLKYENFSLKNDYGDGQCHPKYPKDMLVEDMNVITREYWEDNVDT
RIPNLFAEQSLEENQYENFSSSNNNVEKE-
>PI224770_Vat-x
MDILISVTAKIAEYTVEPVGRQLGYVFFIRSNFQKLKTQVEKLKITRESVQHKIHSARRN
AEDIKPAVEEWLKKVDDFVRESDEILANEGGHGGLCSTYLVQRHKLSRKASKMVDEVLEM
KNEGESFDMVSYKSVIPSVDCSLPKVPDFLDFESRKSIMEQIMDALSDGNVHRIGVYGMG
GVGKTMLVKDILRKIVESKKPFDEVVTSTISQTPDFRSIQGQLADTLGLKFEQETIEGRA
PILRKRLKMERSILVVLDDVWENIDLETIGIPSVEDHTGCKILFTTRNKHLISNQMCANK
IFEIKVLGEDESWNLFKTMAGETVEASDLKPIAIQIVRECAGLPIAITTVAKALRNKPSD
IWNDALNQLKSVDVGMANIGEMERKVYLPLKLSYDCLGYEEVKLLFLLCSMFPEDFPIDV
EELHVYAMGMGFLHGVDTVEKGRCRIKKLVDDLISSSLLQQYSEYGCNYVKMHDMVRDVA
LLIASKNEHVRTLSYVKRSNEEWEEEKLLGNHTAVFIDGLHYPLPKLTLPKVQLLRLVAL
YCWEHNKCVSVVETFFEEMKELKGLVVENVNISLMQRPSDLYSLANIRVLRLERCQLLGS
IDWIGELKKLEILDFSESNITQIPTTMSQLTQLKVLNLSSRE-QLEVIPPNILSKLTKLE
ELNLETFDGWEGEEWYEGRKNASLSELKCLRHLYALNLTIQDEEIMPENLFLVGKLKLQK
FNIRIGCQSKLKYTLPYQNKNRIKNFIGIKMESGRCLDDWIKNLLKRSDNVLLEGSVCSK
VLHSELVSLPNLEKLEIANAESLKMIW---------------------------------
------------------------------------------------------------
------------------------------------------------------------
-----------SNNVPILNSFSKLEEIRIWSCNNLQKVLFHPNMMGILTCLKVLVIRDCK
LLEGIFEVQEPISVVESNNVPILNSFSKLEEIRIWSCNNLQKVLFPPNMMGILTCLKVLE
IIGCNLLEGIFEVQEPISVVESNNVPILNSFSKLEEIRIWSCNNLQKVLFHPNMMGILTC
LKVLVIRNCKLLEGIFEVQEPISVVENNNIPILNSFSKLEEIRIWSCNNLQKVLFPPNMM
GILTCLKVLEIIGCNLLEGIFEVQEPISVVEAS--PIVLQNLSRLKLYNLPNLEYLWSKN
PCELLSLENIKNLTIEECPRLRREYSVKILKPLEDVSIDIKQLMKVIEKEKSADHNMLES
KQWETSSSSKDGVLRLGDGSKLFPYLKSLKLYGFVDYNSTHLPMEMLQILFQLED-FELE
GAFIEEIFPSNILIS---------SSMDLQSLTLSKLPKLKHLWSEECSQNNITSVLQHL
FFLRISDCGRLSSLTLVSSLVCFTNLKDLHVNKCHRLTHLLNPSVATTLVQLEGLTVEEC
KRMSSV---------------FLFQ-----------------------------------
-------------------------------------PNKLLL----------WEMHH--
------------------------------
>Canton_Vat-x
MDILISVTAKIAEYTVEPVLRQLRYVFFIRSNFRELKTQIEKLKITKESVEHNIHSARRN
AEDIKPAVEEWSKKVDDIVGKSEEILAYEGGHGRLCSTNLVQRHKLSRKASKMAYEVRAM
NTEGKSFDTVSYKIVIPSVGCSPTKVPDFLDFDSRKSIVKQIMDALSEDNVHRIGVHGMG
GVGKTMLVNEILRKIGESKKLFDEVVTSTISQTPDFKRIQGELADKLGLKFEQETIKGRA
SILEKRLKMERSILVVLDDVWENIDLKDIGIPSVEDHTGCKILFTTRNKHLISNQMCANK
IFEIKVLGEDESWNLFKTMAGEIVEASDLKPIAIQIVKECAGLPIAITTVAKALRNKSFD
IWNDALNQLKSVDVGMANIGEMEKEVYLPLKLSYDCLGYEEVKLLFLLCSMFPEDFPIDV
EELHVYAMGMGFLHGVDTVGKGRCRIKKLVDDLISSSLLQQYSKYGRNYVKMHDMVRDVA
LLFASQNDHIRILSYVKSLNEEWEEDRLSGNHTAVSIDGLHYPLPKLTLPKVQLLRLVG-
QSWEHKQYVSVVETLFEEMKELKGLVLENVNISLMQRPSDLYSLANIRVLRLQRC-LLGS
IDWIGELKKLEILDFSVSNITQIPTTMSQLTQLKVLNLSYCQ-QLKVIPPNILSKLTKLE
ELNLETFDRWEGEEWYEGRKNASPSELKCLRHLYALNLTIQDEEIMPKDLFLAEELKLQK
FNICIGPN-RLKYT--FGPTNIIKNFIAIKMESGRCSDDWIKNLLKRSNNVFLEGSICSK
VLHSELVSLPNLEKLKIVDAKSLKMIW---------------------------------
------------------------------------------------------------
------------------------------------------------------------
-----------SNNVPILNSFSKLEEIKICSCNNLQKVLFPPNMMGILTCLKVLEIENCE
LLEGIFEVQEPISVVESNNVPILNSFSKLEEIRICSCNNLQKVLFPPNMMGILTCLKFLE
IKNCELLEGIFEVQEPISVVESNNVPILNSFSKLEEIRICSCNNLQKVLFPPNMMGILTC
LKVLEIENCELLEGIFEVQEPISVVESNNVPILNSFSKLEEIRICSCNNVQKVLFPPNMM
GILTCLKFLEIKNCELLEGIFEVQEPISVVEAS--PIVLQNLIRLELYNLPNLEYVWSKN
PCELLSLENIKSLTIEECPRLRREYSVKILKPLQYVSIDIKQLMKVIEKEKSADHNMLES
KQWETSSSSKDGVLRLGDGSKLFPNLGGLKLYGFVDYNSTHLPMEMLQILFQLKDVFELE
GAFIEEIFPSNILIS---------SSMDLHVLILSKLPKLKHLWSEECSQNNITSVLQHL
TELYIFECGGLSS--LVSSLVCFTHLRYLHVNKCHRLTHLLNPSVATTLVQLVGLTVKEC
KRMSSVIEGGST-EEDGNDEMVVFNNLHDLDIVNCSNLTSFYCGRCIIKFPRLKTVFIQK
CPEMKVFSLGIVSTPRLKYKNFSLKNYYDDGQCHPKYPKDMLVEDMNVITREYWEDNVDT
RIPNLFAEQSLEENQYENSSSSNNDVEKE*
>Smith_Perfect_Vat-x
MDILISVTAKIAEYTVEPVLRQLRYVFFIRSNFRELKTQIEKLKITKESVEHNIHSARRN
AEDIKPAVEEWSKKVDDIVGKSEEILAYEGGHGRLCSTNLVQRHKLSRKASKMAYEVRAM
NTEGKSFDTVSYKIVIPSVGCSPTKVPDFLDFDSRKSIVKQIMDALSEDNVHRIGVHGMG
GVGKTMLVNEILRKIGESKKLFDEVVTSTISQTPDFKRIQGELADKLGLKFEQETIKGRA
SILEKRLKMERSILVVLDDVWENIDLKDIGIPSVEDHTGCKILFTTRNKHLISNQMCANK
IFEIKVLGEDESWNLFKTMAGEIVEASDLKPIAIQIVKECAGLPIAITTVAKALRNKSFD
IWNDALNQLKSVDVGMANIGEMEKEVYLPLKLSYDCLGYEEVKLLFLLCSMFPEDFPIDV
EELHVYAMGMGFLHGVDTVGKGRCRIKKLVDDLISSSLLQQYSKYGRNYVKMHDMVRDVA
LLFASQNDHIRILSYVKSLNEEWEEDRLSGNHTAVSIDGLHYPLPKLTLPKVQLLRLVG-
QSWEHKQYVSVVETLFEEMKELKGLVLENVNISLMQRPSDLYSLANIRVLRLQRC-LLGS
IDWIGELKKLEILDFSVSNITQIPTTMSQLTQLKVLNLSYCQ-QLKVIPPNILSKLTKLE
ELNLETFDRWEGEEWYEGRKNASPSELKCLRHLYALNLTIQDEEIMPKDLFLAEELKLQK
FNICIGPN-RLKYT--FGPTNIIKNFIAIKMESGRCSDDWIKNLLKRSNNVFLEGSICSK
VLHSELVSLPNLEKLKIVDAKSLKMIW---------------------------------
------------------------------------------------------------
------------------------------------------------------------
-----------SNNVPILNSFSKLEEIKICSCNNLQKVLFPPNMMGILTCLKVLEIENCE
LLEGIFEVQEPISVVESNNVPILNSFSKLEEIRICSCNNLQKVLFPPNMMGILTCLKFLE
IKNCELLEGIFEVQEPISVVESNNVPILNSFSKLEEIRICSCNNLQKVLFPPNMMGILTC
LKVLEIENCELLEGIFEVQEPISVVESNNVPILNSFSKLEEIRICSCNNVQKVLFPPNMM
GILTCLKFLEIKNCELLEGIFEVQEPISVVEAS--PIVLQNLIRLELYNLPNLEYVWSKN
PCELLSLENIKSLTIEECPRLRREYSVKILKPLQYVSIDIKQLMKVIEKEKSADHNMLES
KQWETSSSSKDGVLRLGDGSKLFPNLGGLKLYGFVDYNSTHLPMEMLQILFQLKDVFELE
GAFIEEIFPSNILIS---------SSMDLHVLILSKLPKLKHLWSEECSQNNITSVLQHL
TELYIFECGGLSS--LVSSLVCFTHLRYLHVNKCHRLTHLLNPSVATTLVQLVGLTVKEC
KRMSSVIEGGST-EEDGNDEMVVFNNLHDLDIVNCSNLTSFYCGRCIIKFPRLKTVFIQK
CPEMKVFSLGIVSTPRLKYKNFSLKNYYDDGQCHPKYPKDMLVEDMNVITREYWEDNVDT
RIPNLFAEQSLEENQYENSSSSNNDVEKE*
>WMR29_Vat-x
MDILISVTAKIAEYTVEPVLRQLRYVFFIRSNFRELKTQIEKLKITRESVLHNIHYARRN
AEDIKPAVEEWLKKVNDIVGKSEEILAYEGGHGKLCSTNLVQRHKLSRKASKMAYEVGEM
NTEGKSFDTVSYKIVIPSVGCSPTKVPDFLDFDSRKSIVKQIMDALSEDNVHRIGVHGMG
GVGKTMLVNEILRKIGESKKLFDEVVTSTISQTSDFKRIQGELADKLGLKFEQETIKGRA
SILEKRLKMERSILVVLDDVWENIDLKDIGIPSVEDHTGCKILFTTRNKDLISNQMCANK
IFEIKVLGEDESWNLFKTMAGEIVEARDLKPIAIQIVRECAGLPIAITTVAKALRNKPSD
IWNDALNQLKSVDVGIANIGEMERRVYLPLKLSYDYLGYEEVKLLFLLCSMFPEDFTIDE
EELHVYAIGMGFLHGVNTVEKVRCRIKKLVEDLISSSLLQQYSEYGCNYVKMHDMIRDVA
LSIASKNEHVRTLSYVKRSNEEWEEEKLSGNHTAVFIDGLHYPLPKLTLPKVQLLRLVG-
QSWEHK-FVSVVETLFEEMKELKGLVLENVNISLMQRPFDLYSLANIRVLLLQRCQLLGS
IDWIGELKKLEILDFSESNITQIPTTMSQLTQLKVLNLSSCE-ELEVIPPNILSKLTKLE
ELNLETFDRWEGEEWYEGRKNASLSELKCLRHLYALNLTIQDEEIMPKDLFLAEELKLQK
FNICIGCQ-S-MYT--FGPPNRIKNFIAMEMESGRCLDDWIKNLLKRSDNVCLKGSICSK
VLHSELVSLPNLEKLEIVNAESLKMIW---------------------------------
------------------------------------------------------------
------------------------------------------------------------
-----------SNNVPILNSFSKLEEIKICSCNNLQKVLFPSNMMDILTCLKVLDIRDCE
LLEGIFEVQEPISVVESNNVPILNSFSKLEEIRICSCNNLQKVLFPPNMMGILPCLEVLD
IRGCELLEGIFEVQEPISVVESNNVPILNSFSKLEEIRIWSCNNLQKVLFPSNMMDILTC
LKVLDIRDCELLEGIFEVQEPISVVESNNVPILNSFSKLEEIRIWSCNNLQKVLFPSNMM
DILTCLKVLDIRDCELLEGIFEVQEPISVVEAS--PIVLQNLSRLKLYNLPNLEYLWSKN
PCELLSLENIKILTIEECPRLRREYSVKI-KPLEDVSIDIKQLMKVIEKEKSADHNMLES
KQWETSSSSKDGVLRLGDGSKLFPNLKSLKLYGFVDYNSTHLPMEMLQILFQLED-FELE
GAFIEEIFPSNILIS---------SDMVLRRLRLYKLPKLKHLWSEECSQNNITSVLQHL
TDVSISECGGLSS--LVSSLVCFTNLKHLHVNKCHRLTHLLNPSVATTLVQLEGLTVKEC
KRMSSVIEGGST-EEDGNDEIIVFNNLRNLSITSCSNITSFYCGRCIIKFPCLEDVYIIE
CPEMKVFSLGTVSTPDLKYTNVYLINDYGYILYHPKYPKDMLVEDMNVIIREYWEDNVDT
RIPNLFAEQSLEENQYENSSSSNNNVEKE*
>DHL92_Piel de Sapo_Vat-3
MDILISVTAKIAEYTVEPVLRQLRYVFFIRSNFRELKTQIEKLKITRESVLHNIHYARRN
AEDIKPAVEEWLKKVNDIVGKSEEILAYEGGHGKLCSTNLVQRHKLSRKASKMAYEVGEM
NTEGKSFDTVSYKIVIPSVGCSPTKVPDFLDFDSRKSIVKQIMDALSEDNVHRIGVHGMG
GVGKTMLVNEILRKIGESKKLFDEVVTSTISQTSDFKRIQGELADKLGLKFEQETIKGRA
SILEKRLKMERSILVVLDDVWENIDLKDIGIPSVEDHTGCKILFTTRNKDLISNQMCANK
IFEIKVLGEDESWNLFKTMAGEIVEARDLKPIAIQIVRECAGLPIAITTVAKALRNKPSD
IWNDALNQLKSVDVGIANIGEMERRVYLPLKLSYDYLGYEEVKLLFLLCSMFPEDFTIDE
EELHVYAIGMGFLHGVNTVEKVRCRIKKLVEDLISSSLLQQYSEYGCNYVKMHDMIRDVA
LSIASKNEHVRTLSYVKRSNEEWEEEKLSGNHTAVFIDGLHYPLPKLTLPKVQLLRLVG-
QSWEHK-FVSVVETLFEEMKELKGLVLENVNISLMQRPFDLYSLANIRVLLLQRCQLLGS
IDWIGELKKLEILDFSESNITQIPTTMSQLTQLKVLNLSSCE-ELEVIPPNILSKLTKLE
ELNLETFDRWEGEEWYEGRKNASLSELKCLRHLYALNLTIQDEEIMPKDLFLAEELKLQK
FNICIGCQ-S-MYT--FGPPNRIKNFIAMEMESGRCLDDWIKNLLKRSDNVCLKGSICSK
VLHSELVSLPNLEKLEIVNAESLKMIW---------------------------------
------------------------------------------------------------
------------------------------------------------------------
-----------SNNVPILNSFSKLEEIKICSCNNLQKVLFPSNMMDILTCLKVLDIRDCE
LLEGIFEVQEPISVVESNNVPILNSFSKLEEIRICSCNNLQKVLFPPNMMGILPCLEVLD
IRGCELLEGIFEVQEPISVVESNNVPILNSFSKLEEIRIWSCNNLQKVLFPSNMMDILTC
LKVLDIRDCELLEGIFEVQEPISVVESNNVPILNSFSKLEEIRIWSCNNLQKVLFPSNMM
DILTCLKVLDIRDCELLEGIFEVQEPISVVEAS--PIVLQNLSRLKLYNLPNLEYLWSKN
PCELLSLENIKILTIEECPRLRREYSVKI-KPLEDVSIDIKQLMKVIEKEKSADHNMLES
KQWETSSSSKDGVLRLGDGSKLFPNLKSLKLYGFVDYNSTHLPMEMLQILFQLED-FELE
GAFIEEIFPSNILIS---------SDMVLRRLRLYKLPKLKHLWSEECSQNNITSVLQHL
TDVSISECGGLSS--LVSSLVCFTNLKHLHVNKCHRLTHLLNPSVATTLVQLEGLTVKEC
KRMSSVIEGGST-EEDGNDEIIVFNNLRNLSITSCSNITSFYCGRCIIKFPCLEDVYIIE
CPEMKVFSLGTVSTPDLKYTNVYLINDYGYILYHPKYPKDMLVEDMNVIIREYWEDNVDT
RIPNLFAEQSLEENQYENSSSSNNNVEKE*
>Charentais Mono_Vat-3
MDILISVTAKIAEYTVEPVLRQLRYVFFIRSNFRELKTQIEKLKITRESVLHNIHYARRN
AEDIKPAVEEWLKKVNDIVGKSEEILAYEGGHGKLCSTNLVQRHKLSRKASKMAYEVGEM
NTEGKSFDTVSYKIVIPSVGCSPTKVPDFLDFDSRKSIVKQIMDALSEDNVHRIGVHGMG
GVGKTMLVNEILRKIGESKKLFDEVVTSTISQTSDFKRIQGELADKLGLKFEQETIKGRA
SILEKRLKMERSILVVLDDVWENIDLKDIGIPSVEDHTGCKILFTTRNKDLISNQMCANK
IFEIKVLGEDESWNLFKTMAGEIVEARDLKPIAIQIVRECAGLPIAITTVAKALRNKPSD
IWNDALNQLKSVDVGIANIGEMERRVYLPLKLSYDYLGYEEVKLLFLLCSMFPEDFTIDE
EELHVYAIGMGFLHGVNTVEKVRCRIKKLVEDLISSSLLQQYSEYGCNYVKMHDMIRDVA
LSIASKNEHVRTLSYVKRSNEEWEEEKLSGNHTAVFIDGLHYPLPKLTLPKVQLLRLVG-
QSWEHK-FVSVVETLFEEMKELKGLVLENVNISLMQRPFDLYSLANIRVLLLQRCQLLGS
IDWIGELKKLEILDFSESNITQIPTTMSQLTQLKVLNLSSCE-ELEVIPPNILSKLTKLE
ELNLETFDRWEGEEWYEGRKNASLSELKCLRHLYALNLTIQDEEIMPKDLFLAEELKLQK
FNICIGCQ-S-MYT--FGPPNRIKNFIAMEMESGRCLDDWIKNLLKRSDNVCLKGSICSK
VLHSELVSLPNLEKLEIVNAESLKMIW---------------------------------
------------------------------------------------------------
------------------------------------------------------------
-----------SNNVPILNSFSKLEEIKICSCNNLQKVLFPSNMMDILTCLKVLDIRDCE
LLEGIFEVQEPISVVESNNVPILNSFSKLEEIRICSCNNLQKVLFPPNMMGILPCLEVLD
IRGCELLEGIFEVQEPISVVESNNVPILNSFSKLEEIRIWSCNNLQKVLFPSNMMDILTC
LKVLDIRDCELLEGIFEVQEPISVVESNNVPILNSFSKLEEIRIWSCNNLQKVLFPSNMM
DILTCLKVLDIRDCELLEGIFEVQEPISVVEAS--PIVLQNLSRLKLYNLPNLEYLWSKN
PCELLSLENIKILTIEECPRLRREYSVKI-KPLEDVSIDIKQLMKVIEKEKSADHNMLES
KQWETSSSSKDGVLRLGDGSKLFPNLKSLKLYGFVDYNSTHLPMEMLQILFQLED-FELE
GAFIEEIFPSNILIS---------SDMVLRRLRLYKLPKLKHLWSEECSQNNITSVLQHL
TDVSISECGGLSS--LVSSLVCFTNLKHLHVNKCHRLTHLLNPSVATTLVQLEGLTVKEC
KRMSSVIEGGST-EEDGNDEIIVFNNLRNLSITSCSNITSFYCGRCIIKFPCLEDVYIIE
CPEMKVFSLGTVSTPDLKYTNVYLINDYGYILYHPKYPKDMLVEDMNVIIREYWEDNVDT
RIPNLFAEQSLEENQYENSSSSNNNVEKE*
>PI414723_Vat-3
MDILISVTAKIAEYTVEPVLRQLRYVFFIRSNFRELKTQIEKLKITRESVLHNIHYARRN
AEDIKPAVEEWLKKVNDIVGKSEEILAYEGGHGKLCSTNLVQRHKLSRKASKMAYEVGEM
NTEGKSFDTVSYKIVIPSVGCSPTKVPDFLDFDSRKSIVKQIMDALSEDNVHRIGVHGMG
GVGKTMLVNEILRKIGESKKLFDEVVTSTISQTSDFKRIQGELADKLGLKFEQETIKGRA
SILEKRLKMERSILVVLDDVWENIDLKDIGIPSVEDHTGCKILFTTRNKDLISNQMCANK
IFEIKVLGEDESWNLFKTMAGEIVEARDLKPIAIQIVRECAGLPIAITTVAKALRNKPSD
IWNDALNQLKSVDVGIANIGEMERRVYLPLKLSYDYLGYEEVKLLFLLCSMFPEDFTIDE
EELHVYAIGMGFLHGVNTVEKVRCRIKKLVEDLISSSLLQQYSEYGCNYVKMHDMIRDVA
LSIASKNEHVRTLSYVKRSNEEWEEEKLSGNHTAVFIDGLHYPLPKLTLPKVQLLRLVG-
QSWEHK-FVSVVETLFEEMKELKGLVLENVNISLMQRPFDLYSLANIRVLLLQRCQLLGS
IDWIGELKKLEILDFSESNITQIPTTMSQLTQLKVLNLSSCE-ELEVIPPNILSKLTKLE
ELNLETFDRWEGEEWYEGRKNASLSELKCLRHLYALNLTIQDEEIMPKDLFLAEELKLQK
FNICIGCQ-S-MYT--FGPPNRIKNFIAMEMESGRCLDDWIKNLLKRSDNVCLKGSICSK
VLHSELVSLPNLEKLEIVNAESLKMIW---------------------------------
------------------------------------------------------------
------------------------------------------------------------
-----------SNNVPILNSFSKLEEIKICSCNNLQKVLFPSNMMDILTCLKVLDIRDCE
LLEGIFEVQEPISVVESNNVPILNSFSKLEEIRICSCNNLQKVLFPPNMMGILPCLEVLD
IRGCELLEGIFEVQEPISVVESNNVPILNSFSKLEEIRIWSCNNLQKVLFPSNMMDILTC
LKVLDIRDCELLEGIFEVQEPISVVESNNVPILNSFSKLEEIRIWSCNNLQKVLFPSNMM
DILTCLKVLDIRDCELLEGIFEVQEPISVVEAS--PIVLQNLSRLKLYNLPNLEYLWSKN
PCELLSLENIKILTIEECPRLRREYSVKI-KPLEDVSIDIKQLMKVIEKEKSADHNMLES
KQWETSSSSKDGVLRLGDGSKLFPNLKSLKLYGFVDYNSTHLPMEMLQILFQLED-FELE
GAFIEEIFPSNILIS---------SDMVLRRLRLYKLPKLKHLWSEECSQNNITSVLQHL
TDVSISECGGLSS--LVSSLVCFTNLKHLHVNKCHRLTHLLNPSVATTLVQLEGLTVKEC
KRMSSVIEGGST-EEDGNDEIIVFNNLRNLSITSCSNITSFYCGRCIIKFPCLEDVYIIE
CPEMKVFSLGTVSTPDLKYTNVYLINDYGYILYHPKYPKDMLVEDMNVIIREYWEDNVDT
RIPNLFAEQSLEENQYENSSSSNNNVEKE*
>Vedrantais_Vat-3
MDILISVTAKIAEYTVEPVLRQLRYVFFIRSNFRELKTQIEKLKITRESVLHNIHYARRN
AEDIKPAVEEWLKKVNDIVGKSEEILAYEGGHGKLCSTNLVQRHKLSRKASKMAYEVGEM
NTEGKSFDTVSYKIVIPSVGCSPTKVPDFLDFDSRKSIVKQIMDALSEDNVHRIGVHGMG
GVGKTMLVNEILRKIGESKKLFDEVVTSTISQTSDFKRIQGELADKLGLKFEQETIKGRA
SILEKRLKMERSILVVLDDVWENIDLKDIGIPSVEDHTGCKILFTTRNKDLISNQMCANK
IFEIKVLGEDESWNLFKTMAGEIVEARDLKPIAIQIVRECAGLPIAITTVAKALRNKPSD
IWNDALNQLKSVDVGIANIGEMERRVYLPLKLSYDYLGYEEVKLLFLLCSMFPEDFTIDE
EELHVYAIGMGFLHGVNTVEKVRCRIKKLVEDLISSSLLQQYSEYGCNYVKMHDMIRDVA
LSIASKNEHVRTLSYVKRSNEEWEEEKLSGNHTAVFIDGLHYPLPKLTLPKVQLLRLVG-
QSWEHK-FVSVVETLFEEMKELKGLVLENVNISLMQRPFDLYSLANIRVLLLQRCQLLGS
IDWIGELKKLEILDFSESNITQIPTTMSQLTQLKVLNLSSCE-ELEVIPPNILSKLTKLE
ELNLETFDRWEGEEWYEGRKNASLSELKCLRHLYALNLTIQDEEIMPKDLFLAEELKLQK
FNICIGCQ-S-MYT--FGPRNRIKNFIAMEMESGRCLDDWIKNLLKRSDNVCLKGSICSK
VLHSELVSLPNLEKLEIVNAESLKMIW---------------------------------
------------------------------------------------------------
------------------------------------------------------------
-----------SNNVPILNSFSKLEEIKICSCNNLQKVLFPSNMMDILTCLKVLDIRDCE
LLEGIFEVQEPISVVESNNVPILNSFSKLEEIRICSCNNLQKVLFPPNMMGILPCLEVLD
IRGCELLEGIFEVQEPISVVESNNVPILNSFSKLEEIRIWSCNNLQKVLFPSNMMDILTC
LKVLDIRDCELLEGIFEVQEPISVVESNNVPILNSFSKLEEIRIWSCNNLQKVLFPSNMM
DILTCLKVLDIRDCELLEGIFEVQEPISVVEAS--PIVLQNLSRLKLYNLPNLEYLWSKN
PCELLSLENIKILTIEECPRLRREYSVKI-KPLEDVSIDIKQLMKVIEKEKSADHNMLES
KQWETSSSSKDGVLRLGDGSKLFPNLKSLKLYGFVDYNSTHLPMEMLQILFQLED-FELE
GAFIEEIFPSNILIS---------SDMVLRRLRLYKLPKLKHLWSEECSQNNITSVLQHL
TDVSISECGGLSS--LVSSLVCFTNLKHLHVNKCHRLTHLLNPSVATTLVQLEGLTVKEC
KRMSSVIEGGST-EEDGNDEIIVFNNLRNLSITSCSNITSFYCGRCIIKFPCLEDVYIIE
CPEMKVFSLEP*------------------------------------------------
------------------------------
>CUM64_Vat-x
MDILISVTAKIAEYTVEPVLRQLRYVFFIRSNFRELKTQIEKLKITRESVLHNIHYARRN
AEDIKPAVEEWLKKVNDIVGKSEEILAYEGGHGKLCSTNLVQRHKLSRKASKMAYEVGEM
NTEGKSFDTVSYKIVIPSVGCSPTKVPDFLDFDSRKSIVKQIMDALSEDNVHRIGVHGMG
GVGKTMLVNEILRKIGESKKLFDEVVTSTISQTSDFKRIQGELADKLGLKFEQETIKGRA
SILEKRLKMERSILVVLDDVWENIDLKDIGIPSVEDHTGCKILFTTRNKDLISNQMCANK
IFEIKVLGEDESWNLFKTMAGEIVEARDLKPIAIQIVRECAGLPIAITTVAKALRNKPSD
IWNDALNQLKSVDVGIANIGEMERRVYLPLKLSYDYLGYEEVKLLFLLCSMFPEDFTIDE
EELHVYAIGMGFLHGVNTVEKVRCRIKKLVEDLISSSLLQQYSEYGCNYVKMHDMIRDVA
LSIASKNEHVRTLSYVKRSNEEWEEEKLSGNHTAVFIDGLHYPLPKLTLPKVQLLRLVGQ
-SWEH-KFVSVVETLFEEMKELKGLVLENVNISLMQRPFDLYSLANIRVLLLQRCQLLGS
IDWIGELKKLEILDFSESNITQIPTTMSQLTQLKVLNLSSC-EELEVIPPNILSKLTKLE
ELNLETFDRWEGEEWYEGRKNASLSELKCLRHLYALNLTIQDEEIMPKDLFLAEELKLQK
FNICIGCQS--MYTFGP--PNRIKNFIAMEMESGRCLDDWIKNLLKRSDNVCLKGSICSK
VLHSELVSLPNLEKLEIVNAESLKMIW---------------------------------
------------------------------------------------------------
------------------------------------------------------------
-----------SNNVPILNSFSKLEEIKICSCNNLQKVLFPSNMMDILTCLKVLDIRDCE
LLEGIFEVQEPISVVESNNVPILNSFSKLEEIRICSCNNLQKVLFPPNMMGILPCLEVLD
IRGCELLEGIFEVQEPISVVESNNVPILNSFSKLEEIRIWSCNNLQKVLFPSNMMDILTC
LKVLDIRDCELLEGIFEVQEPISVVESNNVPILNSFSKLEEIRIWSCNNLQKVLFPSNMM
DILTCLKVLDIRDCELLEGIFEVQEPISVVEAS--PIVLQNLSRLKLYNLPNLEYLWSKN
PCELLSLENIKILTIEECPRLRREYSVKI-KPLEDVSIDIKQLMKVIEKEKSADHNMLES
KQWETSSSSKDGVLRLGDGSKLFPNLKSLKLYGFVDYNSTHLPMEMLQILFQLED-FELE
GAFIEEIFPSNILIS---------SDMVLRRLRLYKLPKLKHLWSEECSQNNITSVLQHL
TDVSISECGGLSS--LVSSLVCFTNLKHLHVNKCHRLTHLLNPSVATTLVQLEGLTVKEC
KRMSSVIEGGST-EEDGNDEIIVFNNLRNLSITSCSNITSFYCGRCIIKFPCLEDVYIIE
CPEMKVFSLGTVSTPDLKYTNVYLINDYGYILYHPKYPKDMLVEDMNVIIREYWEDNVDT
RIPNLFAEQSLEENQYENSSSSNNNVEKE-
