# Supplementary material for: A highly diversified NLR cluster in melon contains homologs that confer powdery mildew and aphid resistance
Source: Hortic Res. 2023 Dec 13;11(1):uhad256. doi: 10.1093/hr/uhad256 (PMC10807702; doi:10.1093/hr/uhad256)
Supplement: Web_Material_uhad256 [file web_material_uhad256.zip › DataS4_5R65aa_CSativus_R2bis_fasta.rtf]

#CC-NBS-LLR1
#LRR2
#LRR3-N-term

>C_Sativus_9930_CDS_out_cluster
MDILVSVTAKIAEYTVVPVGRQLGYVIHIHANFQKLKTQVEKLKDTRESVQQNIYTARRN
AEDIKPAVEKWLKNVDDFVRESDKILANEGGHGRLCSTNLVQRHKLSRKASKMAYEVNEM
KNEGEGFNTVSYKNAIPSVDCSLQKVSDFLDLDSRKLTAEQIMDALSDDNVHRIGVYGMG
GVGKTMLVKEILRKIVESKS-FDEVVTSTISQTPDFKSIQGQLADKLGLKFERETIEGRA
PILRKRLKMERRILVVLDDIWEYIDLETIGIPSVEDHTGCKILFTSRNKHLISNQMCANQ
IFEIKVLGENESWNLFKAMAGKIVEASDLKPIAIQVVRECAGLPIAITTVAKALRNKPSD
IWNDALDQLKSVDVFMTNIGEMDKKVYLSLKLSYDCLGYEEVKLLFLLCSMFPEDFSIDM
EELHVYAMGMGFLHGVDTVVKGRRRIKKLVDDLISSSLLQQYSEYGYNYVKMHDMVRDVA
IFIASKNDHIRTLSYVKRLDEEWKEERLLGNHTVVSIHGLHYPLPKLMLPKVQLLRLDGQ
--WLNNTYVSVVQTFFEEMKELKGLVLEKVNISLLQRPFDLYFLANIRVLRLRGCEL-GS
IDMIGELKRLEILDLSGSNIIQIPTTMGQLTQLKVLNLSNCFNKLEIIPPNILSKLTKLE
ELRLGTFGSWEGEEWYEGRKNASLSELRFLPHLFDLDLTIQDEKIMPKHLFSAEELNLEN
FHITIGCKRERVKNYDGIIKMNYSRILEVKMESEMCLDDWIKFLLKRSEEVHLEGSICSK
VLNSELLDANGFLHLKNLWIFYNSDIQHFIHEKNKPLRKCLSKLEFLYLKNLENLESVIH
GYNNGESPLNNLKNVIVWNCNKLKTLFLNCMLDDVLNLEEIEINYCKKMEVMITVKENEE
TTNHVEFTHLKSLCLWTLPQLHKFCSKVSNTINTCESFFSEEVSLPNLEKLKIWCTKDLK
KIWSNNVLIPNSFSKLKEIDIYSCNNLQKALFSPNMMSILTCLKVLRIEDCKLLEGIFEVQEPISVVE----------------------------------------------------------------------------------------------------------------
------------------------------------------------------------
------------------------------------------------------------
------------------------------TSPIALQTLSELKLYKLPNLEYVWSKDSCE
LQSLVNIKRLTMDECPRLRREYSVKILKQLEALSIDIKQLMEVIGKKKSTDYNRLESKQL
ETSSSKVE-VLQLGDGSELFPKLKTLKLYGFVEDNSTHLPMEIVQNLYQFEKFELEGAFI
EEILPSNILIPMKKQYNARRSKTSQRSWVLSKLPKLRHL-GSECSQKNNDSILQDLTSLS
ISECGGLSS--LVSSSVSFTNLTFLKLNKCDGLTHLLNPSMATTLVQLKQLRIGECKRMS
RIIEGGSSGEEDGNGEIIVFNNLQFLIITSCSNLTSFYRGRCIIQFPCLKHVSLEKCPKM
KSFSFGIVSTSHSKYEKCFFKE--------------------------------------
-------------------------
>WMR29_Vat-1
MDILISVTAKIAEYTVEPVGRQLGYVFFIRSNFQKLKTQVEKLKITRESVQHKIHSARRN
AEDIKPAVEEWLKKVDDFVRESDEILANEGGHGGLCSTYLVQRHKLSRKASKMVDEVLEM
KNEGESFDMVSYKSVIPSVDCSLPKVPDFIDFESRKSIMEQIMDALSDGNVHRIGVYGMG
GVGKTMLVKDILRKIVESKKPFDEVVTSTISQTPDFRSIQGQLADTLGLKLEQETIEGRA
PILRKRLKMERSILVVLDDVWENIDLETIGIPSVEDHTGCKILFTTRNKHLISNQMCANK
IFEIKVLGEDESWNLFKTMAGETVEASDLKPIAIQIVRECAGLPIAITTVAKALRNKPSD
IWNDALNQLKSVDVGVANIGEMERKVYLPLKLSYDCLGYEEVKLLFLLCSMFPEDFTIDV
EELHVYAMGMGFLHGVDTVEKGRCRIKKLVDDLISSSLLQQYSEYRCNYVKMHDMVRDVA
LLIASQNDHIRILSYVKSLNEEWKEDRLSGNHTTVSIDGLHYPLPKLMLPKVQLLRLVGH
YCGGINNRVSVVETFFEEMKELKGLVLENVNISLMQRTSDLYSLANIRVLRLQRCQLLGS
IDWIGELKKLEILDFIGSNISQIPTTMSQLTQLKVLNLSFC-EQLEVIPPNILSKLTKLE
ELNLENFDGWEGEEWYEGRKNASLSELKCLRHLYALNLTIQDEEIMPKDLFLAEELKLQK
FNICIGYQSKLKYTSGP--TNRIKNFIAIKMESGRCLDNWIKNLLKRSDNVFLEGSVCSK
VLHSEL------------------------------------------------------
------------------------------------------------------------
------------------------------------------VTLPNLEKLEIVNAESLK
MIWSNNVPILNSFSKLEEIRIWSCNNLQKVFFLPNMMGILTCLKVLEIEHCELLEGIFEV
QEPISVVESNNVPILNSFSKLEEIRISSCNNLQKVLFPPNMMGILTCLKVLEIEHCELLE
GIFEVQEPISVVESNNVPILNSFSKLEEISICLCNNLQKVLFPPNMMGILTCLKVLEIRD
CELLEGIFEVQEPISVVESNNVPILNSFSKLEEIKISLCNNLQKVLFPPNMIGILTCLKV
LEIGNCELLEGIFEVQEPISVVESNNVPILNSFSKLEEIRICSCNNFQKVLFPANMMGIL
TCLKVLKIEHCELLEGIFEVQEPISVVEAS--PIVLQNLSRLELYNLPNLEYVWSKNPCE
LLSLENIKILTIDKCPRLRREYSVKILKPLEDVSIDIKQLMKVIEKEKSADHNMLESKQW
ETSSSSKDGVLRLGDGSKLFPNLKSLKLYGFVDYNSTHLPMEMLQILFQLEVFELEGAFL
EEIFPSNILIP---------SYMVLRRLALSKLPKLKHLWSEECSQNNITSVLQDLSFLR
ISECGRLSS--LLSSIVCFTNLKHLRVYKCDGLTHLLNPSVATTLVQLEHLRIHECKRMS
SVIEGGST-EEDGNDEIIVFNNLQHLSIFNCSNLTSFYCGRCIIKFPCLRKVYIWDCPEM
KVFSLGIVSTPRLKYKNFYLKNDYDDERCHPKYPKDMLVEDMNVITREYWEDNVDTRIPN
LFAEQSSEENQYENSSSSNNNVEKE
>Anso77_Vat-3
MDILISVTAKIAEYTVEPVGRQLGYVFFIRSNFQKLKTQVEKLKITRESVQHKIHSARRN
AEDIKPAVEEWLKKVDDFVRESDEILANEGGHGGLCSTYLVQRHKLSRKASKMVDEVLEM
KNEGESFDMVSYKSVIPSVDCSLPKVPDFIDFESRKSIMEQIMDALSDGNVHRIGVYGMG
GVGKTMLVKDILRKIVESKKPFDEVVTSTISQTPDFRSIQGQLADTLGLKLEQETIEGRA
PILRKRLKMERSILVVLDDVWENIDLETIGIPSVEDHTGCKILFTTRNKHLISNQMCANK
IFEIKVLGEDESWNLFKTMAGETVEASDLKPIAIQIVRECAGLPIAITTVAKALRNKPSD
IWNDALNQLKSVDVGVANIGEMERKVYLPLKLSYDCLGYEEVKLLFLLCSMFPEDFTIDV
EELHVYAMGMGFLHGVDTVEKGRCRIKKLVDDLISSSLLQQYSEYRCNYVKMHDMVRDVA
LLIASQNDHIRILSYVKSLNEEWKEDRLSGNHTTVSIDGLHYPLPKLMLPKVQLLRLVGH
YCGGINNRVSVVETFFEEMKELKGLVLENVNISLMQRTSDLYSLANIRVLRLQRCQLLGS
IDWIGELKKLEILDFIGSNISQIPTTMSQLTQLKVLNLSFC-EQLEVIPPNILSKLTKLE
ELNLENFDGWEGEEWYEGRKNASLSELKCLRHLYALNLTIQDEEIMPKDLFLAEELKLQK
FNICIGYQSKLKYTSGP--TNRIKNFIAIKMESGRCLDNWIKNLLKRSDNVFLEGSVCSK
VLHSEL------------------------------------------------------
------------------------------------------------------------
------------------------------------------VTLPNLEKLEIVNAESLK
MIWSNNVPILNSFSKLEEIRIWSCNNLQKVFFLPNMMGILTCLKVLEIEHCELLEGIFEV
QEPISVVESNNVPILNSFSKLEEIRISSCNNLQKVLFPPNMMGILTCLKVLEIEHCELLE
GIFEVQEPISVVESNNVPILNSFSKLEEISICLCNNLQKVLFPPNMMGILTCLKVLEIRD
CELLEGIFEVQEPISVVESNNVPILNSFSKLEEIKISLCNNLQKVLFPPNMIGILTCLKV
LEIGNCELLEGIFEVQEPISVVESNNVPILNSFSKLEEIRICSCNNFQKVLFPANMMGIL
TCLKVLKIEHCELLEGIFEVQEPISVVEAS--PIVLQNLSRLELYNLPNLEYVWSKNPCE
LLSLENIKILTIDKCPRLRREYSVKILKPLEDVSIDIKQLMKVIEKEKSADHNMLESKQW
ETSSSSKDGVLRLGDGSKLFPNLKSLKLYGFVDYNSTHLPMEMLQILFQLEVFELEGAFL
EEIFPSNILIP---------SYMVLRRLALSKLPKLKHLWSEECSQNNITSVLQDLSFLR
ISECGRLSS--LLSSIVCFTNLKHLRVYKCDGLTHLLNPSVATTLVQLEHLRIHECKRMS
SVIEGGST-EEDGNDEIIVFNNLQHLSIFNCSNLTSFYCGRCIIKFPCLRKVYIWDCPEM
KVFSLGIVSTPRLKYKNFYLKNDYDDERCHPKYPKDMLVEDMNVITREYWEDNVDTRIPN
LFAEQSSEENQYENSSSSNNNVEKE
>Edisto47_Vat-x
MDILISVTAKIAEYTVEPVGRQLGYVFFIRSNFQKLKTQVEKLKITRESVQHKIHSARRN
AEDIKPAVEEWLKKVDDFVRESDEILANEGGHGGLCSTYLVQRHKLSRKASKMVDEVLEM
KNEGESFDMVSYKSVIPSVDCSLPKVPDFIDFESRKSIMEQIMDALSDGNVHRIGVYGMG
GVGKTMLVKDILRKIVESKKPFDEVVTSTISQTPDFRSIQGQLADTLGLKLEQETIEGRA
PILRKRLKMERSILVVLDDVWENIDLETIGIPSVEDHTGCKILFTTRNKHLISNQMCANK
IFEIKVLGEDESWNLFKTMAGETVEASDLKPIAIQIVRECAGLPIAITTVAKALRNKPSD
IWNDALNQLKSVDVGVANIGEMERKVYLPLKLSYDCLGYEEVKLLFLLCSMFPEDFTIDV
EELHVYAMGMGFLHGVDTVEKGRCRIKKLVDDLISSSLLQQYSEYRCNYVKMHDMVRDVA
LLIASQNDHIRILSYVKSLNEEWKEDRLSGNHTTVSIDGLHYPLPKLMLPKVQLLRLVGH
YCGGINNRVSVVETFFEEMKELKGLVLENVNISLMQRTSDLYSLANIRVLRLQRCQLLGS
IDWIGELKKLEILDFIGSNISQIPTTMSQLTQLKVLNLSFC-EQLEVIPPNILSKLTKLE
ELNLENFDGWEGEEWYEGRKNASLSELKCLRHLYALNLTIQDEEIMPKDLFLAEELKLQK
FNICIGYQSKLKYTSGP--TNRIKNFIAIKMESGRCLDNWIKNLLKRSDNVFLEGSVCSK
VLHSEL------------------------------------------------------
------------------------------------------------------------
------------------------------------------VTLPNLEKLEIVNAESLK
MIWSNNVPILNSFSKLEEIRIWSCNNLQKVFFLPNMMGILTCLKVLEIEHCELLEGIFEV
QEPISVVESNNVPILNSFSKLEEIRISSCNNLQKVLFPPNMMGILTCLKVLEIEHCELLE
GIFEVQEPISVVESNNVPILNSFSKLEEISICLCNNLQKVLFPPNMMGILTCLKVLEIRD
CELLEGIFEVQEPISVVESNNVPILNSFSKLEEIKISLCNNLQKVLFPPNMIGILTCLKV
LEIGNCELLEGIFEVQEPISVVESNNVPILNSFSKLEEIRICSCNNFQKVLFPANMMGIL
TCLKVLKIEHCELLEGIFEVQEPISVVEAS--PIVLQNLSRLELYNLPNLEYVWSKNPCE
LLSLENIKILTIDKCPRLRREYSVKILKPLEDVSIDIKQLMKVIEKEKSADHNMLESKQW
ETSSSSKDGVLRLGDGSKLFPNLKSLKLYGFVDYNSTHLPMEMLQILFQLEVFELEGAFL
EEIFPSNILIP---------SYMVLRRLALSKLPKLKHLWSEECSQNNITSVLQDLSFLR
ISECGRLSS--LLSSIVCFTNLKHLRVYKCDGLTHLLNPSVATTLVQLEHLRIHECKRMS
SVIEGGST-EEDGNDEIIVFNNLQHLSIFNCSNLTSFYCGRCIIKFPCLRKVYIWDCPEM
KVFSLGIVSTPRLKYKNFYLKNDYDDERCHPKYPKDMLVEDMNVITREYWEDNVDTRIPN
LFAEQSSEENQYENSSSSNNNVEKE
>PI414723_Vat-5
MDILISVTAKIAEYTVEPVGRQLGYVFFIRSNFQKLKTQVEKLKITRESVQHKIHSARRN
AEDIKPAVEEWLKKVDDFVRESDEILANEGGHGGLCSTYLVQRHKLSRKASKMVDEVLEM
KNEGESFDMVSYKSVIPSVDCSLPKVPDFIDFESRKSIMEQIMDALSDGNVHRIGVYGMG
GVGKTMLVKDILRKIVESKKPFDEVVTSTISQTPDFRSIQGQLADTLGLKFEQETIEGRA
PILRKRLKMERSILVVLDDVWENIDLETIGIPSVEDHTGCKILFTTRNKHLISNQMCANK
IFEIKVLGEDESWNLFKTIAGETVEASDLKPIAIQIARECAGLPIAITTVAKALRNKPSE
IWNDALNQLKSVDVGMANIGEMERKVYLPLKMSYDCLGYEEVKLLFLLCSMFPEDFTIDV
EELHVYAMGMGFLHGVDTVEKGRCRIKKLVDDLISSSLLQQYSKYGCNYVKMHDMVRDVA
LLIASQNDHIRILSYVKSLNEEWKEDRLSGNHTTVSIDGLHYPLPKLTFPKVQLLRLVAQ
SWWEHNESVSVVETFFEEMKELKGLVLENVNISLMQRTSDLYSLANIRVLHLQRCQLLGS
IDWIGELKKLEILDFRGSNITQIPTTMSQLTQLKVLNLSSC-NQLKVIPPNILSKLTKLE
ELSLETFDRWEGEEWYEGRKNASLSELKCLRHLYALNLTIQDEEIMPKDLFLAEELKLQK
FNICIGYQSKLKYTFGP--TNRIKNFIAIKMESGRCLDNWIKNLLKRSDNVFLEGSICSK
VLHSEL------------------------------------------------------
------------------------------------------------------------
------------------------------------------VSLPNLEKLEIANAESLK
MIWSNNVPILNSFSKLEEIRIWSCNNLQKVLFPPNMMGILTCLKVLEIKGCNLLEGIFEV
QEPISVVESNNVPILNSFSKLEEIRIWSCNNLQKVLFPPNMMGILTCLKVLDIEDCELLE
GIFEVQEPISVVESNNVPILNSFSKLEKIRIWSCNNLQKVLFPPNMMGILTCLKVLDIED
CELLEGIFEVQEPISVVESNNLPILNSFSKLEEIRIWSCNNLQKVLFPPNMMGILTCLKV
LDIRYCGLLEGIFEVQEPISVVETNNVPILNSFSKLEEIRIWSCNNLQKVLFPPNMMGIL
TCLKVLEIIGCNLLEGIFEVQEPISVVEAS--PIVLQNLSRLKLYNLPNLEYLWSKNPCE
LLSLENIKILTIEECPRLRREYSVKILKPLEYVRIDIKQLMKVIEKEKSADHNMFESKQW
ETSSSSKDGVLRLGDGSKLFPNLKKLKLYGFVDYNSTHLPMEMLQILFQLKHFELEGAFI
EEVFPSNILIP---------SYMVLRRLTLSKLPKLKHLWSEECSQNNITSVLQHLTDVS
ISECGGLSS--LVSSLVCFTNLKDLHVNKCHRLTHLLNPSMATTLVQLEYLTIEECKRMS
SVIEGGST-EEDGNDEMVVFNNLQHLYIFNCSNLTSFYCGRCIIKFPCLRQVDIWNCSEM
KVFSLGIVSTPRLKYENFYLKNDYDDERCHPKYPKDMLVEDMNVITREYWEDNVDTRIPN
LFAEQSLEENRSENSSSSKNNVEKE
>90625_Vat-y
MDILISVIAKIAEYTVEPVGRQLGYVFFIRSNFQKLKTQVEKLKITRESVQHKIHSARRN
AEDIKPAVEEWLKKVDDFVRESDEILADEGGHGGLCSTYLVQRHKLSRKASKMVDEVLEM
KNEGESFDMVSYKSVIPSVDCSLPKVPDFLDFESRKSIMEQIMDALSDGNVHRIGVYGMG
GVGKTMLVKDILRKIVESKKPFDEVVTSTISQTPDFRSIQGQLADTLGLKFEQETIEGRA
PILRKRLKMERSILVVLDDVWENIDLETIGIPSVEDHTGCKILFTTRNKHLISNQMCANK
IFEIKVLGEDESWNLFKTMAGETVEASDLKPIAIQIARECAGLPIAITTVAKALRNKPSE
IWNDALNQIKSVDVGMANIGEMEKKVYLSLKLSYDCLGYEEVKLLFLLCSMFPEDFPIDV
EELHVYAMGMGFLHGVDTVVKGRCRIKKLVDDLISSSLLQQYSEYGCNYVKMHDMVRDVA
LLIASKNEHVRTLSYVKRSNEEWEEEKLLGNHTAVFIDGLHYPLPKLTLPKVQLLRLVAK
YCWEHSKRVSVVETFFEEMKELKGLVVENVNISLMQRPSDVYSLANIRVLRLERCQLLGS
IDWIGELKKLEILDFSESNIAQIPTTMSQLTQLKVLNLSSC-EQLEVIPPNILSKLTKLE
ELDLETFDGWEGEEWYEGRKNASLSELKCLRHLYALSLTIQDEEIMPENLFLVGKLKLQK
FNICIGCEGKLKYTFAYKNKNRIKNFIGIKMESGRCLDDWIKNLLKRSDNVLLEGSVCSK
VLHSEL------------------------------------------------------
------------------------------------------------------------
------------------------------------------VSLPNLEKLKIVNAESLK
MIWSNNVPILNSFSKLEEIRIRSCNNLQKVLFPPNMMGILTCLKDLEIEDCELLEGIFEV
QEPISVLESNNLPILNSFSKLEKIIIASCNNLQKVLFPSNMMDILTCLKVLEIRRCELLE
GIFEVQEPISVVESNNVPILNSFSKLEKIRIWSCNNLQKVLFPSNMMDILTCLKVLEIRR
CELLEGIFEVQEPISVVESNNVPILNSFSKLEKIIIASCNNLQKVLFPSNMMDILTCLKV
LEIRRCELLEGIFEVQEPISVVESNNLPILNSFSKLEEIRISSCNNLQKVLFPPNMMGIL
TCLKVLEIQYCELLEGIFEVQEPISVVEAS--PIVFQNLSRLQLYNLPNLEYVWSKNPCE
LLSLENIKILTIDKCPRLRREYSVKILKPLEDVSIDIKQLMKVIEKEKSADHNMLESKQW
ETSSSSKDGVLRLGDGSKLFPNLKKLKLYGFVDYNSTHLPMEMLQILFQLKHFELEGAFI
EEVFPSNILIP---------SYMVLRRLTLSKLPKLRHLWSEECSQNNITSVLQHLTDVS
ISECGGLSS--LVSSLVCFTNLKNLHVNKCHRLTHLLNPSVATTLVQLEGLTVEECKRMS
SVIEEGST-EEDGNDEMVVFNNLQHLYIFNCSNLTSFYCGRCIVKFPCLERVFIQNCPEM
KVFSLGIVSTPRLKYEKFTLMNDYDDKWCHPKYPKDMLVEDMNVITREYWEDNVDTGIPN
LFAEQSLEENRSENSSSSKNNVEKE
>PI414723_Vat-4
MDILISVTAKIAEYTVEPVGRQLGYVFFIRSNFQKLKTQVEKLKITRESVQHKIHSARRN
AEDIKPAVEEWLKKVDDFVRESDEILANEGGHGGLCSTYLVQRHKLSRKASKMVDEVLEM
KNEGESFDMVSYKSVIPSVDCSLPKVPDFIDFESRKSIMEQIMDALSDGNVHRIGVYGMG
GVGKTMLVKDILRKIVESKKPFDEVVTSTISQTPDFRSIQGQLADTLGLKLEQETIEGRA
PILRKRLKMERSILVVLDDVWENIDLETIGIPSVEDHTGCKILFTTRNKHLISNQMCANK
NFEIKVLGEDESWNLFKTMAGETVEASDLKPIAIQIARECAGLPIAITTVAKALRNKPSE
IWNDALNQIKSVDVGMANIGEMEKKVYLSLKLSYDCLGYEEVKLLFLLCSMFPEDFPIDV
EELHVYAMGMGFLHGVDTVVKGRCRIKKLVDDLISSSLLQQYSEYGCNYVKMHDMVRDVA
LLIASKNEHVRTLSYVKRSNEEWEEEKLLGNHTAVSIDGLHYPLPKLTLPKVQLLRLVAQ
YCWEHNKRVSVVETFFEEMKELKGLVVENVNISLMQRPSDLYSLANIRVLRLQRCQLLGS
IDWIGELKKLEILDFSGSNITQIPTTMSQLTQLKVLNLSSC-EQLEVIPPNILSKLTKLE
ELNLETFDGWEGEEWYEGRKNASLSELKCLRHLYALKLTIQDEEIMPKNLFLVGKLKLQK
FNICIGCQSKLKYTFAY--KNRIKNFIGIKMESGRCLDDWIKNLLNRSDNVLLEGSVCSK
VLHSEL------------------------------------------------------
------------------------------------------------------------
------------------------------------------VSLPNLEKLEIVNAKSLK
MIWSNNVPILNSFSKLEEIKIYSCNNLQKVLFPPNMMDILTCLKVLEIKNCDLLEGIFEA
QEPISVVESNNLPILNSFSKLEEIRIWSCNNLQKVLFPSNMMGILPCLKVLDIRGCELLE
GIFEVQEPISVVESNSVPILNSFSKLEKIRIWSCNNLQKILFPSNMMGILTCLKVLEIRD
CELLEGIFEVQEPISVVESNNLPILNSFSKLEEIRIWSCNNLQKVLFPPNMMGILTCLKV
LKIEHCELLEGIFEVQEPISVVETNNVPILNSFSKLEEIRIGSCNNLQKVLFPPNMMGIL
TCLKVLEIRHCNLLEGIFEVQEPISVVEAS--PIVLQNLSGLELYNLPNLEYVWSKNPCE
LLSLENIKFFTIDKCPRLRREYSVKILEPLEDVSIDIKQLMKVIEKEKSADHNMLESKQW
ETSSSSKDGVLRLGDGSKLFPNLKSLKLYGFVDYNSTHLPMEMLQILFQLVVFELEGAFI
EEIFPSNILIP---------SYMVLRRLSLSKLPKLKHLWSEECSQNNITSVLQHLFFLR
ISDCGRLSSLTLVSSLVCFTNLKDLWVDECDGLTHLLNPSMATTLVQLEYLTIEECKRMS
SVIEGGST-EEDGNDEMVVFNNLQHLYIFNCSNLTSFYCGRCIIKFPCLERVFIQNCPEM
KVFSLGIVSTPRLKYEKFTLMNDYDDKWCHPKYPKDMLVEDMNVITREYWEDNVDTRIPN
LFAEQSLEENRSENSSSSKNNVEKE
>Charentais_Mono_Vat-1
MDILISVTAKIAEYTVEPVLRQLRYVFFIRSNFCELKTQIEKLKITKESVEHNIHSARRN
AEDIKPAVEEWSKKVDDIVGKSEEILAYEGGHGRLCSTNLVQRHKLSRKASKMAYEVRAM
NTEGKSFDTVSYKIVIPSVGCSPTKVPDFLDFDSRKSIVKQIMDALSEDNVHRIGVHGMG
GVGKTMLVNEILRKIGESKKLFDEVVTSTISQTPDFKRIQGELADKLGLKFEQETIKGRA
SILEKRLKMERSILVVLDDVWENIDLKDIGIPSVEDHTGCKILFTTRNKDLISNQMCANK
IFEIKVLGEDESWNLFKTIAGEIVEARDLKPIAIQIVRECAGLPIAITTVAKALRNQSSD
IWNDALNQLKSVDVGIANIGEMERRVYLPLKLSYDYLGYEEVKLLFLLCSMFPEDFTIDE
EELHVYAIGMGFLHGVNTVEKVRCRIKKLVEDLISSSLLQQYSEYGRNYVKMHDMIRDVA
LSIASKNEHVRTLSYVKRSNEEWEEEKLSGNHTAVFIDGLHYPLPKLTLPKVQLLTLVGQ
-SWEI-KCVSVVETLFEEMKELKGLVLENVNISLMQRPSDLYSLANIRVLCLRRCQLLGS
IDWIGELKKLEILDFSGSNITQIPTTMSQLTQLKVLNLSSC-NQLKVIPPNILSKLTKLE
ELSLETFDRWEGEEWYEGRKNASLSELKCLRHLYALNLTIQDEEIMPKDLFLAEELKLQK
FNICIGCQS--MYTFGP--PNRIKNFIAMEMESGRCLDDWIKNLLKRSDNVCLKGSICSK
VLHSEL------------------------------------------------------
------------------------------------------------------------
------------------------------------------VSLPNLEKLEIVNAESLK
MIWSNNVPILNSFSKLEEITICSCNNLQKVLFPSNMMDILTCLKVLDIRDCELLEGIFEV
QEPISVVESNNVPILNSFSKLEEITIWSCNNLQKVLFPPNMMDILTCLKVLDIRDCELLE
GIFEVQEPISVVESNNVPILNSFSKLEEIRICSCNNLQKVLFPPNMMGILTCLKVLEIEH
CELLEGIFEVQEPISVVESNNVPILNSFSKLEEIRICSCNNLQKVLFPPNMMGILTCLKV
LEIEHCELLEGIFEVQEPISVVESNNVPILNSFSKLEEIRICSCNNLQKVLFPPNMMGIL
TCLKFLEIKNCELLEGIFEVQEPISVVEAS--PIVLQNLIRLELYNLPNLEYVWSKNPCE
LLSLENIKSLTIEECPRLRREYSVKILKPLQYVSIDIKQLMKVIEKEKSADHNMLESKQW
ETSSSSKDGVLRLGDGSKLFPNLKSLKLYGFVDYNSTHLPMEMLQILFQLKHFELEGAFI
EEIFPSNILIS---------SSMDLQSLTLYKLPKLKHLWSEECSRNNITSVLQHLIFLR
ISDCGRLSSLTLVSSLVCFTNLKSLAVYKCDRLTHLLNPSMATTLVQLQDLTINECKRMR
SVIEEGST-EEDGNDEMVVFNNLQDLYIFNCSNLTSFYCGRCIIKFPCLREVYIWDCPEM
KVFSLGIVSTPRLKYGNFYLKNDYDDERCHPKYPKDMLVEDMNVITREYWEDNVDTRIPN
LFAEQSLEENRSENSSSSKNNVEKE
>Vedrantais_Vat-1
MDILISVTAKIAEYTVEPVLRQLRYVFFIRSNFCELKTQIEKLKITKESVEHNIHSARRN
AEDIKPAVEEWSKKVDDIVGKSEEILAYEGGHGRLCSTNLVQRHKLSRKASKMAYEVRAM
NTEGKSFDTVSYKIVIPSVGCSPTKVPDFLDFDSRKSIVKQIMDALSEDNVHRIGVHGMG
GVGKTMLVNEILRKIGESKKLFDEVVTSTISQTPDFKRIQGELADKLGLKFEQETIKGRA
SILEKRLKMERSILVVLDDVWENIDLKDIGIPSVEDHTGCKILFTTRNKDLISNQMCANK
IFEIKVLGEDESWNLFKTIAGEIVEARDLKPIAIQIVRECAGLPIAITTVAKALRNQSSD
IWNDALNRLKSVDVGIANIGEMERRVYLPLKLSYDYLGYEEVKLLFLLCSMFPEDFTIDE
EELHVYAIGMGFLHGVNTVEKVRCRIKKLVEDLISSSLLQQYSEYGRNYVKMHDMIRDVA
LSIASKNEHVRTLSYVKRSNEEWEEEKLSGNHTAVFIDGLHYPLPKLTLPKVQLLTLVGQ
-SWEI-KCVSVVETLFEEMKELKGLVLENVNISLMQRPSDLYSLANIRVLCLRRCQLLGS
IDWIGELKKLEILDFSGSNITQIPTTMSQLTQLKVLNLSSC-NQLKVIPPNILSKLTKLE
ELSLETFDRWEGEEWYEGRKNASLSELKCLRHLYALNLTIQDEEIMPKDLFLAEELKLQK
FNICIGCQS--MYTFGP--PNRIKNFIAMEMESGRCLDDWIKNLLKRSDNVCLKGSICSK
VLHSEL------------------------------------------------------
------------------------------------------------------------
------------------------------------------VSLPNLEKLEIVNAESLK
MIWSNNVPILNSFSKLEEITICSCNNLQKVLFPSNMMDILTCLKVLDIRDCELLEGIFEV
QEPISVVESNNVPILNSFSKLEEITIWSCNNLQKVLFPPNMMDILTCLKVLDIRDCELLE
GIFEVQEPISVVESNNVPILNSFSKLEEIRICSCNNLQKVLFPPNMMGILTCLKVLEIEH
CELLEGIFEVQEPISVVESNNVPILNSFSKLEEIRICSCNNLQKVLFPPNMMGILTCLKV
LEIEHCELLEGIFEVQEPISVVESNNVPILNSFSKLEEIRICSCNNLQKVLFPPNMMGIL
TCLKFLEIKNCELLEGIFEVQEPISVVEAS--PIVLQNLIRLELYNLPNLEYVWSKNPCE
LLSLENIKSLTIEECPRLRREYSVKILKPLQYVSIDIKQLMKVIEKEKSADHNMLESKQW
ETSSSSKDGVLRLGDGSKLFPNLKSLKLYGFVDYNSTHLPMEMLQILFQLKHFELEGAFI
EEIFPSNILIS---------SSMDLQSLTLYKLPKLKHLWSEECSRNNITSVLQHLIFLR
ISDCGRLSSLTLVSSLVCFTNLKSLAVYKCDRLTHLLNPSMATTLVQLQDLTINECKRMR
SVIEEGST-EEDGNDEMVVFNNLQDLYIFNCSNLTSFYCGRCIIKFPCLREVYIWDCPEM
KVFSLGIVSTPRLKYGNFYLKNDYDDERCHPKYPKDMLVEDMNVITREYWEDNVDTRIPN
LFAEQSLEENRSENSSSSKNNVEKE
>Invernizo_Vat-y
MDILISVTAKIAEYTVEPVGRQLGYVFFIRSNFQKLKTQVEKLKITRESVQHKIHSARRN
AEDIKPAVEEWLKKVDDFVRESDEILANEGGHGGLCSTYLVQRHKLSRKASKMVDEVLEM
KNEGESFDMVSYKSVIPSVDCSLPKVPDFIDFESRKSIMEQIMDALSDGNVHRIGVYGMG
GVGKTMLVKDILRKIVESKKPFDEVVTSTISQTPDFRSIQGQLADTLGLKLEQETIEGRA
PILRKRLKMERSILVVLDDVWENIDLETIGIPSVEDHTGCKILFTTRNKHLISNQMCANK
IFEIKVLGEDESWNLFKTMAGETVEASDLKPIAIQIVRECAGLPIAITTVAKALRNKPSD
IWNDALNQLKSVDVGVANIGEMERKVYLPLKLSYDCLGYEEVKLLFLLCSMFPEDFTIDV
EELHVYAMGMGFLHGVDTVEKGRCRIKKLVDDLISSSLLQQYSEYRCNYVKMHDMVRDVA
LLIASQNDHIRILSYVKSLNEEWKEDRLSGNHTTVSIDGLHYPLPKLMLPKVQLLRLVGH
YCGGINNRVSVVETFFEEMKELKGLVLENVNISLMQRTSDLYSLANIRVLRLQRCQLLGS
IDWIGELKKLEILDFIGSNISQIPTTMSQLTQLKVLNLSFC-EQLEVIPPNILSKLTKLE
ELNLENFDGWEGEEWYEGRKNASLSELKCLRHLYALNLTIQDEEIMPKDLFLAEELKLQK
FNICIGYQSKLKYTSGP--TNRIKNFIAIKMESGRCLDNWIKNLLKRSDNVFLEGSVCSK
VLHSEL------------------------------------------------------
------------------------------------------------------------
------------------------------------------VTLPNLEKLEIVNAESLK
MIWSNNVPILNSFSKLEEIRIWSCNNLQKVFFLPNMMGILTCLKVLEIEHCELLEGIFEV
QEPISVVESNNVPILNSFSKLEEIRISSCNNLQKVLFPPNMMGILTCLKVLEIEHCELLE
GIFEVQEPISVVESNNVPILNSFSKLEEISICLCNNLQKVLFPPNMMGILTCLKVLEIRD
CELLEGIFEVQEPISVVESNNVPILNSFSKLEEIKISLCNNLQKVLFPPNMIGILTCLKV
LEIGNCELLEGIFEVQEPISVVESNNVPILNSFSKLEEIRICSCNNFQKVLFPANMMGIL
TCLKVLKIEHCELLEGIFEVQEPISVVEAS--PIVLQNLSRLELYNLPNLEYVWSKNPCE
LLSLENIKILTIDKCPRLRREYSVKILKPLEDVSIDIKQLMKVIEKEKSADHNMLESKQW
ETSSSSKDGVLRLGDGSKLFPNLKSLKLYGFVDYNSTHLPMEMLQILFQLEVFELEGAFL
EEIFPSNILIP---------SYMVLRRLALSKLPKLKHLWSEECSQNNITSVLQDLSFLR
ISECGRLSS--LLSSIVCFTNLKHLRVYKCDGLTHLLNPSVATTLVQLEHLRIHECKRMS
SVIEGGST-EEDGNDEIIVFNNLQHLSIFNCSNLTSFYCGRCIIKFPCLRKVYIWDCPEM
KVFSLGIVSTPRLKYKNFYLKNDYDDERCHPKYPKDMLVEDMNVITREYWEDNVDTRIPN
LFAEQSS------------------
>PI124112_Vat-x
MDILISVTAKIAEYTVEPVGRQLGYVFFIRSNFQKLKTQVEKLKITRESVQHKIHSARRN
AEDIKPAVEEWLKKVDDFVRESDEILANEGGHGGLCSTYLVQRHKLSRKASKMVDEVLEM
KNEGESFDMVSYKSVIPSVDCSLPKVPDFIDFESRKSIMEQIMDALSDGNVHRIGVYGMG
GVGKTMLVKDILRKIVESKKPFDEVVTSTISQTPDFRSIQGQLADTLGLKLEQETIEGRA
PILRKRLKMERSILVVLDDVWENIDLETIGIPSVEDHTGCKILFTTRNKHLISNQMCANK
IFEIKVLGEDESWNLFKTMAGETVEASDLKPIAIQIVRECAGLPIAITTVAKALRNKPSD
IWNDALNQLKSVDVGVANIGEMERKVYLPLKLSYDCLGYEEVKLLFLLCSMFPEDFTIDV
EELHVYAMGMGFLHGVDTVEKGRCRIKKLVDDLISSSLLQQYSEYRCNYVKMHDMVRDVA
LLIASQNDHIRILSYVKSLNEEWKEDRLSGNHTTVSIDGLHYPLPKLMLPKVQLLRLVGH
YCGGINNRVSVVETFFEEMKELKGLVLENVNISLMQRTSDLYSLANIRVLRLQRCQLLGS
IDWIGELKKLEILDFIGSNISQIPTTMSQLTQLKVLNLSFC-EQLEVIPPNILSKLTKLE
ELNLENFDGWEGEEWYEGRKNASLSELKCLRHLYALNLTIQDEEIMPKDLFLAEELKLQK
FNICIGYQSKLKYTSGP--TNRIKNFIAIKMESGRCLDNWIKNLLKRSDNVFLEGSVCSK
VLHSEL------------------------------------------------------
------------------------------------------------------------
------------------------------------------VTLPNLEKLEIVNAESLK
MIWSNNVPILNSFSKLEEIRIWSCNNLQKVFFLPNMMGILTCLKVLEIEHCELLEGIFEV
QEPISVVESNNVPILNSFSKLEEIRISSCNNLQKVLFPPNMMGILTCLKVLEIEHCELLE
GIFEVQEPISVVESNNVPILNSFSKLEEISICLCNNLQKVLFPPNMMGILTCLKVLEIRD
CELLEGIFEVQEPISVVESNNVPILNSFSKLEEIKISLCNNLQKVLFPPNMIGILTCLKV
LEIGNCELLEGIFEVQEPISVVESNNVPILNSFSKLEEIRICSCNNFQKVLFPANMMGIL
TCLKVLKIEHCELLEGIFEVQEPISVVEAS--PIVLQNLSRLELYNLPNLEYVWSKNPCE
LLSLENIKILTIDKCPRLRREYSVKILKPLEDVSIDIKQLMKVIEKEKSADHNMLESKQW
ETSSSSKDGVLRLGDGSKLFPNLKSLKLYGFVDYNSTHLPMEMLQILFQLEVFELEGAFL
EEIFPSNILIP---------SYMVLRRLALSKLPKLKHLWSEECSQNNITSVLQDLSFLR
ISECGRLSS--LLSSIVCFTNLKHLRVYKCDGLTHLLNPSVATTLVQLEHLRIHECKRMS
SVIEGGST-EEDGNDEIIVFNNLQHLSIFNCSNLTSFYCGRCIIKFPCLRKVYIWDCPEM
KVFSLGIVSTPRLKYKNFYLKNDYDDERCHPKYPKDMLVEDMNVITREYWEDNVDTRIPN
LFAEQSSEENQYENSSSSNNNVEKE
>CUM64_Vat-x
MDILISVTAKIAEYTVEPVLRQLRYVFFIRSNFCELKTQIEKLKITKESVEHNIHSARRN
AEDIKPAVEEWSKKVDDIVGKSEEILAYEGGHGRLCSTNLVQRHKLSRKASKMAYEVRAM
NTEGKSFDTVSYKIVIPSVGCSPTKVPDFLDFDSRKSIVKQIMDALSEDNVHRIGVHGMG
GVGKTMLVNEILRKIGESKKLFDEVVTSTISQTPDFKRIQGELADKLGLKFEQETIKGRA
SILEKRLKMERSILVVLDDVWENIDLKDIGIPSVEDHTGCKILFTTRNKDLISNQMCANK
IFEIKVLGEDESWNLFKTIAGEIVEARDLKPIAIQIVRECAGLPIAITTVAKALRNQSSD
IWNDALNQLKSVDVGIANIGEMERRVYLPLKLSYDYLGYEEVKLLFLLCSMFPEDFTIDE
EELHVYAIGMGFLHGVNTVEKVRCRIKKLVEDLISSSLLQQYSEYGRNYVKMHDMIRDVA
LSIASKNEHVRTLSYVKRSNEEWEEEKLSGNHTAVFIDGLHYPLPKLTLPKVQLLTLVGQ
-SWEI-KCVSVVETLFEEMKELKGLVLENVNISLMQRPSDLYSLANIRVLCLRRCQLLGS
IDWIGELKKLEILDFSGSNITQIPTTMSQLTQLKVLNLSSC-NQLKVIPPNILSKLTKLE
ELSLETFDRWEGEEWYEGRKNASLSELKCLRHLYALNLTIQDEEIMPKDLFLAEELKLQK
FNICIGCQS--MYTFGP--PNRIKNFIAMEMESGRCLDDWIKNLLKRSDNVCLKGSICSK
VLHSEL------------------------------------------------------
------------------------------------------------------------
------------------------------------------VSLPNLEKLEIVNAESLK
MIWSNNVPILNSFSKLEEITICSCNNLQKVLFPSNMMDILTCLKVLDIRDCELLEGIFEV
QEPISVVESNNVPILNSFSKLEEITIWSCNNLQKVLFPPNMMDILTCLKVLDIRDCELLE
GIFEVQEPISVVESNNVPILNSFSKLEEIRICSCNNLQKVLFPPNMMGILTCLKVLEIEH
CELLEGIFEVQEPISVVESNNVPILNSFSKLEEIRICSCNNLQKVLFPPNMMGILTCLKV
LEIEHCELLEGIFEVQEPISVVESNNVPILNSFSKLEEIRICSCNNLQKVLFPPNMMGIL
TCLKFLEIKNCELLEGIFEVQEPISVVEAS--PIVLQNLIRLELYNLPNLEYVWSKNPCE
LLSLENIKSLTIEECPRLRREYSVKILKPLQYVSIDIKQLMKVIEKEKSADHNMLESKQW
ETSSSSKDGVLRLGDGSKLFPNLKSLKLYGFVDYNSTHLPMEMLQILFQLKHFELEGAFI
EEIFPSNILIS---------SSMDLQSLTLYKLPKLKHLWSEECSRNNITSVLQHLIFLR
ISDCGRLSSLTLVSSLVCFTNLKSLAVYKCDRLTHLLNPSMATTLVQLQDLTINECKRMR
SVIEEGST-EEDGNDEMVVFNNLQDLYIFNCSNLTSFYCGRCIIKFPCLREVYIWDCPEM
KVFSLGIVSTPRLKYGNFYLKNDYDDERCHPKYPKDMLVEDMNVITREYWEDNVDTRIPN
LFAEQSLEENRSENSSSSKNNVEKE
>PI282448_Vat-x
MDILISVTAKIAEYTVEPVGRQLGYVFFIRSNFQKLKTQVEKLKITRESVQHKIHSARRN
AEDIKPAVEEWLKKVDDFVRESDEILANEGGHGGLCSTYLVQRHKLSRKASKMVDEVLEM
KNEGESFDMVSYKSVIPSVDCSLPKVPDFIDFESRKSIMEQIMDALSDGNVHRIGVYGMG
GVGKTMLVKDILRKIVESKKPFDEVVTSTISQTPDFRSIQGQLADKLGLKFEQETIEGRA
TILRKRLKMERSILVVLDDVWEYIDLETIGIPSVEDHTGCKILFTTRIKHLISNQMCANK
IFEIKVLGKDESWNLFKAMAGDIVDASDLKPIAIRIVRECAGLPIAITTVAKALRNKPSD
IWNDALDQLKTVDVGMANIGEMEKKVYLSLKLSYDCLGYEEVKLLFLLCSMFPEDFSIDV
EGLHVYAMGMGFLHGVDTVVKGRRRIKKLVDDLISSSLLQQYSEYGCNYVKMHDMVRDVA
LLIASKNEHVRTLSYVKRSNEEWEEEKLLGNHTAVFIDGLHYPLPKLTLPKVQLLRLVAK
YCWEHNKRVSVVETFFEEMKELKGLVVENVNISLMQRPSDVYSLANIRVLRLERCQLLGS
IDWIGELKKLEILDFSESNITQIPTTMSQLTQLKVLNLSFC-EQLEVIPPNILSKLTKLE
ELDLETFDGWEGEEWYEGRKNASLSELKCLRHLYALNLTIQDEEIMPKDLFLAEELKLQK
FNIFIGCQSKLKYTFES--TNRIKNFIAIKMESGRCLDDWIKNLLKRSDNVHLEGSICSK
FLHLEL------------------------------------------------------
------------------------------------------------------------
------------------------------------------VSLPNLETLEIVNAESLK
MIWSNNVPILNSFSKLEEIRIWSCNNLQKLLFPPNMMGILTCLKVLEIRHCKLLEVIFEV
QEPISVVESNNVPILNSFSKLEEIRIWSCNNLQKVLFPSNMMGILTCLKVLEIRHCKLLE
GIFEVQEPISVVESNNVPILNSFSKLKEITIWSCNNLQKVLFPSNMMGILTCLKVLEIRD
CKLLEGIFEVQEPISVVESNNVPILNSFSKLEEIRIWSCNNLQKVLFPPNMMGILTCLKV
LYIEDCELLEGIFEVQEPINVVESNNVPILNSFSKLEKIIIGSCNNLQKVLFSSNMMGIL
TCLKVLEIRHCNLLEGIFEVQEPISVVEAS--PIVLQNLSGLELYNLPNLEYVWSKNPCE
LLSLENIKFLTIDKCPRLRREYSVKILKPLEDVSIDIKQLMKVIEKEKSADHNMLESKQW
ETSSSSKDGVLRLGDGSKLFPNLKSLKLYGFVDYNSTHLPMEMLQILFQLKHFELEGAFI
EEIFPSNILIS---------SSMDLQSLTLYKLPKLKHLWSEECSRNNITSVLQHLIFLR
ISDCGRLSSLTLVSSLVCFTNLKSLAVYKCDRLTHLLNPSMATTLVQLQDLTINECKRMR
SVIEEGST-EEDGNDEMVVFNNLQDLYIFNCSNLTSFYCGRCIIKFPCLREVYIWDCPEM
KVFSLGIVSTPRLKYGNFYLKNDYDDERCHPKYPKDMLVEDMNVITREYWEDNVDTRIPN
LFAEQSLEENRSENSSSSKNNVEKE
